# Retrograde sulfur flow from glucosinolates to cysteine in *Arabidopsis thaliana*

**DOI:** 10.1073/pnas.2017890118

**Published:** 2021-05-25

**Authors:** Ryosuke Sugiyama, Rui Li, Ayuko Kuwahara, Ryo Nakabayashi, Naoyuki Sotta, Tetsuya Mori, Takehiro Ito, Naoko Ohkama-Ohtsu, Toru Fujiwara, Kazuki Saito, Ryohei Thomas Nakano, Paweł Bednarek, Masami Yokota Hirai

**Affiliations:** ^a^Metabolic Systems Research Team, RIKEN Center for Sustainable Resource Science, Yokohama 230-0045, Japan;; ^b^College of Life Sciences, Northeast Agricultural University, Harbin, 150030, China;; ^c^Metabolomics Research Group, RIKEN Center for Sustainable Resource Science, Yokohama 230-0045, Japan;; ^d^Department of Applied Biological Chemistry, Graduate School of Agricultural and Life Sciences, The University of Tokyo, Tokyo 113-8657, Japan;; ^e^Institute of Agriculture, Tokyo University of Agriculture and Technology, Tokyo 183-8509, Japan;; ^f^Institute of Global Innovation research, Tokyo University of Agriculture and Technology, Tokyo 183-8509, Japan;; ^g^Plant Molecular Science Center, Chiba University, Chiba 260-8675, Japan;; ^h^Department of Plant Microbe Interactions, Max Planck Institute for Plant Breeding Research, 50829 Cologne, Germany;; ^i^Institute of Bioorganic Chemistry, Polish Academy of Sciences, 61-704, Poznań, Poland

**Keywords:** specialized metabolism, stress response, glucosinolate, sulfur

## Abstract

Specialized (secondary) metabolic pathways in plants have long been considered one-way routes of leading primary metabolite precursors to bioactive end products. Conversely, endogenous degradation of such “end” products in plant tissues has been observed following environmental stimuli, including nutrition stress. Therefore, it is of general interest whether specialized metabolites can be reintegrated into primary metabolism to recover the invested resources, especially in the case of nitrogen- or sulfur-rich compounds. Here, we demonstrate that endogenous glucosinolates (GLs), a class of sulfur-rich plant metabolites, are exploited as a sulfur source by the reallocation of sulfur atoms to primary metabolites such as cysteine in *Arabidopsis thaliana*. Tracer experiments using ^34^S- or deuterium-labeled GLs depicted the catabolic processing of GL breakdown products in which sulfur is mobilized from the thioglucoside group in GL molecules, potentially accompanied by the release of the sulfate group. Moreover, we reveal that beta-glucosidases BGLU28 and BGLU30 are the major myrosinases that initiate sulfur reallocation by hydrolyzing particular GL species, conferring sulfur deficiency tolerance in *A. thaliana*, especially during early development. The results delineate the physiological function of GL as a sulfur reservoir, in addition to their well-known functions as defense chemicals. Overall, our findings demonstrate the bidirectional interaction between primary and specialized metabolism, which enhances our understanding of the underlying metabolic mechanisms via which plants adapt to their environments.

## Significance

Specialized (secondary) metabolites have been largely considered bioactive “end” products synthesized from primary metabolites. We report biochemical evidence of a retrograde flow of sulfur atoms from specialized metabolites (glucosinolates) to primary metabolites (cysteine) in *Arabidopsis thaliana*. The reaction begins with glucosinolate breakdown by specific beta-glucosidases, which facilitates sulfur deficiency tolerance, demonstrating a physiological advantage of utilizing specialized metabolites as nutrient reservoirs. Our findings address the breadth of turnover systems in nature and enhance our understanding of how plants coordinate primary and specialized metabolism under different environmental conditions.

Specialized (secondary) metabolites play critical roles in environmental adaptation. In plants, their concentrations in specific tissues/organelles are regulated using distinct strategies, for example, biosynthesis, modification for activation/inactivation, transport, and breakdown (1–4). Since such dynamic regulatory systems are crucial for the adaptation of their multiple biological/chemical activities in response to different stress factors (2, 3, 5), there is a burgeoning interest in how catabolic systems regulate or decrease endogenous compound levels, in addition to the associated biosynthetic pathways, which have been the primary focus of research in the field of study.

Regarding the catabolic breakdown of specialized metabolites, researchers have long speculated whether specialized metabolites can be reintegrated into primary metabolism. Rapid decreases in metabolite concentrations in plant tissues have been reported in a broad range of compounds following environmental stimuli, including nutrition stress (4). Such observations imply that essential elements in specialized metabolites could be recovered to exploit the invested resources (6, 7). For example, hydrogen cyanide from cyanogenic glycosides may be assimilated into asparagine (8–10), and a recent study demonstrated a retrograde carbon flow from a flavonoid compound to ubiquinone potentially via heme-dependent oxidation (11). Apart from these, however, genetic or biochemical evidence of such catabolic pathways has rarely been reported. In addition, several past studies put the hypothesis into question: for example, nicotine exogenously fed to *Nicotiana sylvestris* does not improve plant growth under nitrogen deficiency (12). Consequently, it remains unclear whether the endogenous storage of specialized metabolites confers physiological advantage as a nutrient reservoir, especially in the case of compounds rich in hetero atoms such as nitrogen and sulfur.

**Fig. 1.**
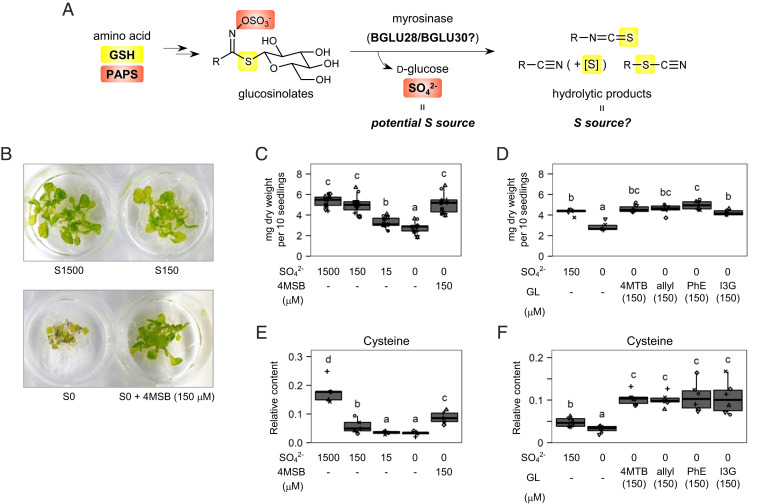
GLs exploited as a sulfur source in *Arabidopsis thaliana*. (*A*) Potential sulfur reallocation by GL–myrosinase system. Two sulfur atoms common in GL molecules are donated from GSH (yellow) and 3′-phosphoadenosine-5′-phosphosulfate (PAPS, orange) during biosynthesis. Hydrolysis of GLs by myrosinases, which may include BGLU28 and BGLU30, triggers generation of SO_4_^2-^, a major sulfur source in plants. (*B*–*F*) Exogenous GL as the sole sulfur source rescued the growth of Col-0 seedlings under sulfur-deficient conditions. (*B*) Images of 14-d-old seedlings cultured at S1500, S150, and S0 conditions (1,500, 150, and 0 μM SO_4_^2-^, respectively) or S0 with 150 μM 4MSB used in the following analysis. (*C* and *D*) Dry weights of the 14-d-old seedlings as the sum of 10 plants cultured at different sulfur concentrations with or without 4MSB (*C*, *n* = 11) or with different GL species supplied at 150 μM under S0 condition (*D*, *n* = 6). (*E* and *F*) Relative contents of Cys in plant tissues. *n* = 5 in *E*, and *n* = 6 in *F*. Point shapes indicate individual experimental batches. Letters indicate statistical significance corresponding to two-tailed *t* tests based on an LMM with batches as a random factor, followed by a correction for multiple comparisons controlling false discovery rate (*P* < 0.05). Abbreviations of GLs correspond to those listed in Table 1.

Glucosinolate (GL) is a class of sulfur- and nitrogen-containing specialized metabolite distributed in the order Brassicales. GLs have a complex catabolic system corresponding to their diverse physiological roles. GLs commonly have a central carbon that is bound to a sulfur atom of thioglucose, a sulfate (SO_4_^2-^) group through an aldoxime linkage, and an amino acid–derived side chain (Fig. 1*A*) (13). Hydrolysis of the thioglucosidic bond by a specific class of beta-glucosidases (BGLUs), called myrosinases, triggers the release of isothiocyanates (ITCs), as well as other volatiles such as nitriles in the presence of specifier proteins (Fig. 1*A*) (14). Such breakdown products function as defense chemicals against herbivores as well as broad biotic/abiotic stress factors, and exhibit bioactivities beneficial to humans, including anticancer properties; thus, GLs have attracted considerable attention from researchers (15, 16).

The potential GL function as a sulfur reservoir has long been explored. In addition to the sulfur-rich structures, 1) GLs account for a considerable proportion (up to 30%) of total sulfur concentrations in species of the family Brassicaceae, whose growth is influenced substantially by environmental sulfur levels (17); 2) sulfur deficiency down-regulates the expression of GL biosynthetic genes and actual GL concentrations (18, 19); and 3) lack of endogenous GLs causes severe growth defects under low sulfur availability in *Arabidopsis thaliana* (20). Moreover, nitrile formation under such conditions has attracted interest because homogenates of *A. thaliana* Columbia-0 at early developmental stages can produce simple nitriles via GL breakdown more dominantly than ITCs (21), and the expression of *NITRILE SPECIFIER PROTEIN 5* is up-regulated under sulfur deficiency (18). However, direct evidence that sulfur is recruited from GLs or their hydrolytic products, in addition to the underlying molecular mechanisms of GL turnover and the roles of specific myrosinases under low sulfur conditions, is still missing.

*BGLU*s potentially encoding myrosinases are distributed exclusively in Brassicales, including 22 out of 47 *BGLU*s in *A. thaliana* (22, 23). To date, seven *At*BGLUs have been reported to hydrolyze GLs with different physiological functions. THIOGLUCOSIDE GLUCOHYDROLASE 1 (TGG1/BGLU38) and TGG2/BGLU37 are abundant in leaves and are accumulated in cells separated from those accumulating GLs, whose predominantly studied function is chemical defense against herbivores (24). TGG4/BGLU34 and TGG5/BGLU35 in roots potentially have an additional function associated with auxin biosynthesis (25), whereas TGG6/BGLU36, which is inactive in some accessions, may have played a pollen defense role in ancestors (26). In addition, PEN2/BGLU26, localized at peroxisomal and mitochondrial membranes, is a key enzyme that triggers plant immune responses (27, 28). PYK10/BGLU23 is enriched in endoplasmic reticulum bodies and is essential for defense against herbivory, possibly coordinating with related homologs, BGLU18 and BGLU21 (22, 29, 30). Furthermore, expression levels of other *AtBGLU*s belonging to Brassicales-specific subclasses vary during developmental stages and under biotic or abiotic stress (22, 31), suggesting that a broad range of BGLUs function as myrosinases to confer the diverse GL functions. Notably, the potential roles of BGLU28 and BGLU30 in sulfur reallocation from GL molecules have been discussed based on their transcriptional up-regulation under sulfur deficiency (32, 33). A recent study on single- and double-knockout mutants of these *BGLU*s supported the hypothesis with respective metabolic phenotypes and reduced growth performance of mutants when compared with the wild type (WT) under such conditions (34). However, their enzymatic properties and actual contributions to GL catabolism are yet to be characterized.

## Results

We first assessed the potential role of exogenous GL as a sulfur source in *A. thaliana*. WT *A. thaliana* (Col-0 accession) seeds were cultured in nutrient solutions containing SO_4_^2-^ at varying concentrations (0, 15, 150, and 1,500 μM; hereafter referred to as S0, S15, S150, and S1500, respectively) or with 150 μM glucoraphanin (4-methylsulfinyl-*n*-butyl glucosinolate, 4MSB), one of the most abundant GLs in mature Col-0 plants (35). The S0 and S15 conditions resulted in severe growth defects in 14-d-old seedlings, whereas plants cultured under 150 μM 4MSB with S0 media exhibited biomass trends similar to those of plants cultured under S150 or S1,500 conditions (Fig. 1 *B* and *C*). The rescued growth phenotype was also observed in the presence of GL species possessing different types of side chains (Fig. 1*D* and Table 1), indicating that GLs supplied in the culture media were consumed as a sulfur source to maintain *A. thaliana* seedling growth. Notably, the concentrations of primary sulfur metabolites such as cysteine (Cys) and glutathione (GSH) in the seedlings cultured with external GLs tended to be higher than those cultured with an equimolar concentration of SO_4_^2-^ (Fig. 1 *E* and *F* and *SI Appendix*, Fig. S1). The results suggest that not only the sulfate group but also other sulfur atoms in GL molecules could be exploited as a sulfur source. Conversely, the side chains of applied GLs did not make a significant difference in the results despite the different number of sulfur atoms in their structures, which could be due to the excess GL supplements compared to the endogenous GL contents: 300 nmol was applied in the media containing 10 seeds while the total GL content in one Col-0 dry seed is ∼1.0 to 1.5 nmol (36).

**Table 1.** GL species analyzed in the present study

Abbreviation	Name	Common name
3MSP	3-Methylsulfinyl-*n*-propyl glucosinolate	Glucoiberin
4MSB	4-Methylsulfinyl-*n*-butyl glucosinolate	Glucoraphanin
5MSP	5-Methylsulfinyl-*n*-pentyl glucosinolate	Glucoalyssin
6MSH	6-Methylsulfinyl-*n*-hexyl glucosinolate	Glucohesperalin
7MSH	7-Methylsulfinyl-*n*-heptyl glucosinolate	Glucoibarin
8MSO	8-Methylsulfinyl-*n*-octyl glucosinolate	Glucohirsutin
3MTP	3-Methylthio-*n*-propyl glucosinolate	Glucoiberverin
4MTB	4-Methylthio-*n*-butyl glucosinolate	Glucoerucin
5MTP	5-Methylthio-*n*-pentyl glucosinolate	Glucoberteroin
6MTH	6-Methylthio-*n*-hexyl glucosinolate	Glucolesquerellin
7MTH	7-Methylthio-*n*-heptyl glucosinolate	
8MTO	8-Methylthio-*n*-octyl glucosinolate	
3OHP	3-Hydroxy-*n*-propyl glucosinolate	
4OHB	4-Hydroxy-*n*-butyl glucosinolate	
3OBzP	3-Benzoyloxy-*n*-propyl glucosinolate	Glucomalcomin
4OBzB	4-Benzoyloxy-*n*-butyl glucosinolate	
PhE	2-Phenylethyl glucosinolate	Gluconasturtiin
I3G	Indol-3-ylmethyl glucosinolate	Glucobrassicin
1MI3G	1-Methoxyindol-3-ylmethyl glucosinolate	Neoglucobrassicin
4MI3G	4-Methoxyindol-3-ylmethyl glucosinolate	4-Methoxyglucobrassicin
4OHI3G^a^	4-Hydroxyindol-3-ylmethyl glucosinolate	4-Hydroxyglucobrassicin
		
Allyl*^,^^†^	Allyl glucosinolate	Sinigrin
4MSB(en)*^,^^†^	4-Methylsulfinyl-3-butenyl glucosinolate	Glucoraphenin
Bn*^,^^†^	Benzyl glucosinolate	Glucotropaeolin

* Not tested in the metabolite analysis.

^†^ Not detected in Col-0.

**Fig. 2.**
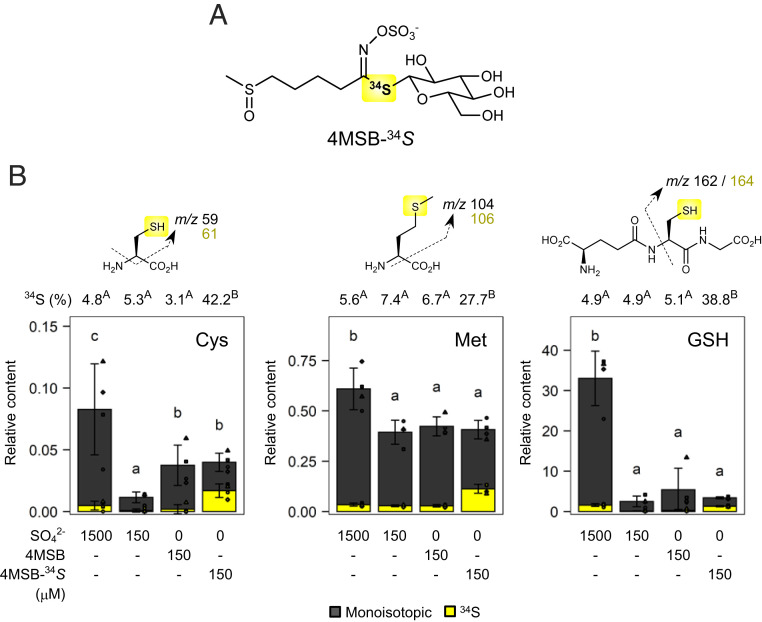
Sulfur reallocation from the thioglucoside group in GLs to primary metabolites. (*A*) Structure of 4MSB labeled with ^34^S at the thioglucosidic bond (4MSB-^34^*S*). (*B*) Relative contents of Cys, Met, and GSH in 14-d-old Col-0 seedlings cultured with SO_4_^2-^, 4MSB, or 4MSB-^34^*S* in the respective nutrient solution (*n* = 4). Dashed arrows indicate MS/MS fragment ions used for the calculation of each metabolite. Contents of monoisotopic compound and the ^34^S isotope are shown in dark gray and yellow, respectively, as a stacked bar chart. Mean abundance of the ^34^S isotope relative to the total content of each metabolite is shown above as numbers. Point shapes indicate individual experimental batches. Letters indicate statistical significance in total metabolite content (lowercase) and abundance of the ^34^S isotope (uppercase) corresponding to two-tailed *t* tests, as applied in Fig. 1 (*P* < 0.05). Error bars indicate SD.

To investigate whether the sulfur atom at the thioglucosidic bond also functions as a sulfur reservoir, an isotope-labeled 4MSB (4MSB-^34^*S*) was synthesized using thiourea-^34^*S* (Fig. 2*A* and *SI Appendix*, Scheme S1). When Col-0 seeds were cultured for 14 d under 150 μM 4MSB-^34^*S* as the sole sulfur source, Cys-^34^*S* accounted for 42% of the total Cys content in the tissue extracts (Fig. 2*B*). Similarly, the abundance of methionine-^34^*S* (Met-^34^*S*) and GSH-^34^*S* reached 28% and 39% of the total Met and GSH contents, respectively (Fig. 2*B*), indicating that the breakdown of external 4MSB reallocates the sulfur atom at the thioglucosidic bond to primary metabolism. If the exogenous 4MSB-^34^*S* was fully assimilated, it would provide up to 30 nmol of ^34^S to each plant. Based on the absolute Cys and GSH concentrations under the S1500 condition (*SI Appendix*, Table S1), Cys-^34^*S* and GSH-^34^*S* contents under 4MSB-^34^*S* feeding are estimated to be 0.076 and 0.150 nmol per seedling, respectively. It implies that ∼0.75% of the ^34^S supplied remained in the tissues as free Cys or GSH. Given Cys and GSH account for ∼0.7% of total sulfur content (excluding SO_4_^2-^) in Col-0 seedlings at sulfur-rich condition (34), ^34^S may be distributed to sulfur-containing biomolecules at a similar ratio.

We also assessed the transfer of ^34^S to other GL species which have a direct biosynthetic link to 4MSB (*SI Appendix*, Fig. S2*A*) (37). Only 4-methylthio-*n*-butyl glucosinolate (4MTB) accumulated the +2 Da isotope under 4MSB-^34^*S* feeding (*SI Appendix*, Fig. S2*B*), consistent with the previous findings that the enzymatic conversions from 4MSB to 4-hydroxy-*n*-butyl glucosinolate or 3-butenyl glucosinolate are unlikely to function in Col-0 seedlings (37). Therefore, it remains unclear whether direct modification of the 4MSB side chain can facilitate reallocation of the Met-derived sulfur atom. Overall, our results reveal that at least two sulfur atoms in GL molecules can be mobilized under sulfur deficiency; one would be released as inorganic SO_4_^2-^ and the other ends up in primary metabolism, potentially through GL hydrolytic products such as ITCs.

**Fig. 3.**
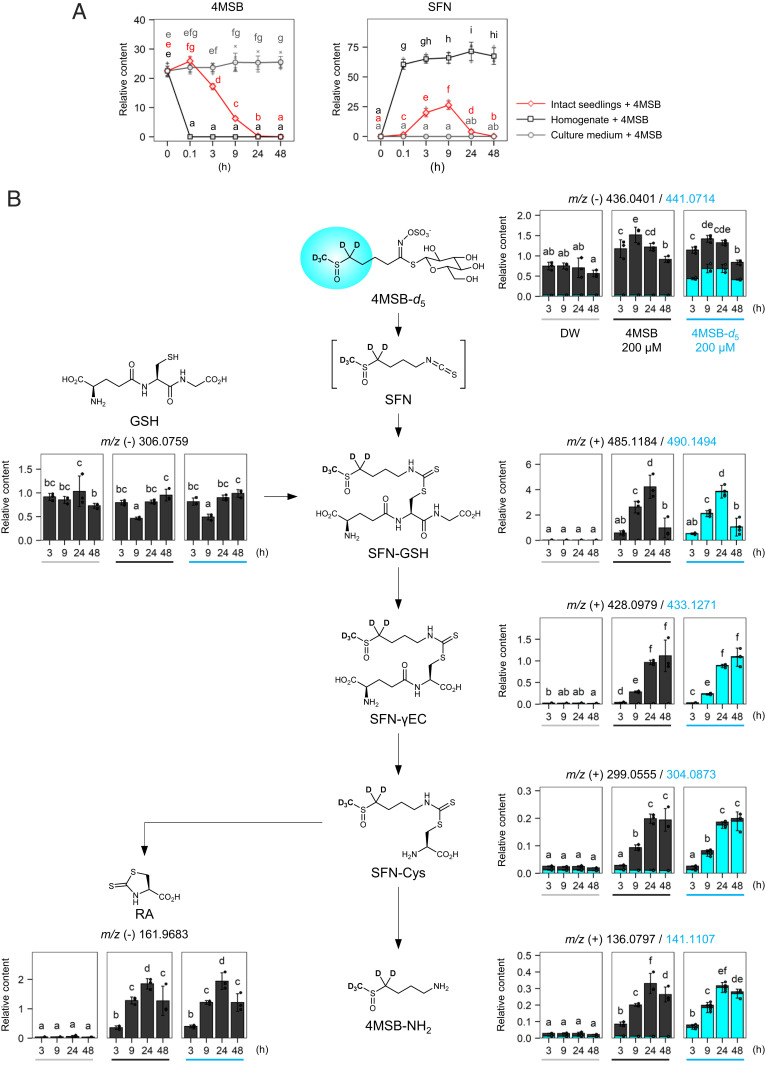
Processing of the GL breakdown products within plant tissues. (*A*) Relative contents of 4MSB (*Left*) and sulforaphane (SFN, *Right*) in S1500 culture media. 4MSB at 100 μM was incubated with intact or homogenized 8-d-old Col-0 seedlings or with S1500 medium only (*n* = 3). (*B*) Potential intermediates of the isothiocyanate processing detected in untargeted metabolomics coupled with deuterated 4MSB (4MSB-*d*_5_) treatment. Relative abundance of monoisotopic (dark gray) and +5 Da (blue) compounds in 8-d-old Col-0 seedlings treated with DW (solvent control) or 200 μM 4MSB or 4MSB-*d*_5_ are shown as stacked bar charts at different time points with their *m/z* values recorded in positive (+) or negative (−) ion modes (*n* = 3). SFN-GSH, SFN-γEC, and SFN-Cys, conjugates of SFN with GSH, with gamma-glutamylcysteine, and with Cys, respectively; 4MSB-NH_2_, 4-methylsulfinyl-*n*-butyl amine. Point shapes indicate individual experimental batches. Letters indicate statistical significance in total metabolite content corresponding to two-tailed *t* tests, as applied in Fig. 1 (*P* < 0.05). Error bars indicate SD.

Subsequently, we monitored the processing of the GL hydrolytic products in plant tissues to explore the metabolic pathway via which the sulfur atom in the thioglucoside group is reallocated to primary metabolism. We observed distinct responses of intact and homogenized seedlings to 4MSB treatment. When 100 μM 4MSB was added to 8-d-old seedlings cultured under the S1500 condition, complete consumption of 4MSB in the medium was observed within 24 h, accompanied by a transient increase in the corresponding ITC, sulforaphane (SFN, 4-methylsulfinyl-*n*-butyl isothiocyanate) (Fig. 3*A*). In contrast, homogenates prepared from the same number of seedlings with the culture media hydrolyzed 4MSB more rapidly, whereas SFN in the mixture remained stable for at least 48 h, indicating that only intact tissues could uptake or modify ITCs (Fig. 3*A*). Hence, we attempted to identify the intermediates produced from SFN in the intact plants using another isotopic 4MSB labeled with five deuterium atoms at the side chain (4MSB-*d*_5_) (Fig. 3*B* and *SI Appendix*, Scheme S2). Metabolite profiles of the 8-d-old seedlings treated with 200 μM 4MSB, 200 μM 4MSB-*d*_5_, or solvent control (deionized water [DW]) for 3, 9, 24, or 48 h were recorded using a liquid chromatography coupled with a high-resolution quadrupole time-of-flight mass spectrometry (LC–QTOF/MS) (38), which displayed 1,378 and 792 peaks in positive and negative ion modes, respectively (Dataset S1).

The data included more than 50 pairs of records with a mass difference corresponding to the deuterium label and a similar retention time, for example, *m/z* 485.1184 and 490.1494 at 2.741 min in the positive ion mode (*SI Appendix*, Fig. S3 and Table S2). Monoisotopic and labeled forms of such ion pairs were accumulated specifically in the plant samples treated with 4MSB and 4MSB-*d*_5_, respectively (Fig. 3*B*). Therefore, we performed tandem MS (MS/MS)-based structural analyses of such ion pairs and inferred the potential intermediate products to be 4-methylsulfinyl-*n*-butyl amine (4MSB-NH_2_, *m/z* 136.0797 and 141.1107 [M+H]^+^) and conjugates of SFN with GSH (SFN-GSH, *m/z* 485.1184 and 490.1494 [M+H]^+^), SFN with gamma-glutamylcysteine (SFN-γEC, *m/z* 428.0979 and 433.1271 [M+H]^+^), and SFN with Cys (SFN-Cys, *m/z* 299.0555 and 304.0873 [M+H]^+^) (Fig. 3*B* and *SI Appendix*, Fig. S4). In addition, a transient decrease in free GSH (*m/z* 306.0759 [M−H]^−^) at 9 h and time-dependent accumulation of raphanusamic acid (RA, *m/z* 161.9683 [M−H]^−^) were observed after both 4MSB treatments in the same manner regardless of the deuterium label (Fig. 3*B* and *SI Appendix*, Table S3). The retention time and MS/MS spectra of the products were consistent with those of the authentic standards (*SI Appendix*, Fig. S5). Accumulation of SFN-GSH was the maximum after 24 h, while SFN-γEC and SFN-Cys seemed to accumulate at later periods. Genetic and chemical inhibition of GSH biosynthesis in the *pad2-1* mutant (39) and by buthionine sulfoximine treatment caused significant delays in the accumulation of such intermediates, probably due to slower generation of SFN-GSH, confirming the GSH requirement in the pathway (*SI Appendix*, Fig. S6). The findings above, in combination with their chemical structures, suggest that the downstream pathway after 4MSB hydrolysis begins with the conjugation of SFN and GSH followed by stepwise cleavage of Gly and γ-Glu residues from SFN-GSH, after which SFN-Cys is finally cyclized to generate 4MSB-NH_2_ and RA (Fig. 3*B*).

The potential intermediates (SFN-GSH, SFN-γEC, SFN-Cys, and RA) were also detected in the 14-d-old seedlings grown with 4MSB-^34^*S* as the +2 and +4 Da isotopes, supporting the view that sulfur reallocation from GLs is achieved via this GSH-dependent pathway (*SI Appendix*, Fig. S7*A*). The +2 Da forms were exclusively labeled at the sulfur atom derived from the ITC group, while detection of the +4 Da forms suggested that Cys produced from a mobilized ^34^S is recruited to GSH biosynthesis to process the remaining SFN. Notably, the 14-d-old seedlings treated with 4MTB, allyl glucosinolate, or phenylethyl glucosinolate (PhE) accumulated the corresponding amine compounds as well as RA in the tissues, while such metabolites were not detected under treatment with indol-3-ylmethyl glucosinolate (I3G) (*SI Appendix*, Fig. S8). It implies that aliphatic and benzenic but not indolic GLs potentially follow a similar catabolic pathway in mobilizing sulfur atoms. Moreover, we detected only trace amounts of RA when almost all endogenous GLs were depleted in the *cyp79b2 cyp79b3 myb28 myb29* mutant (40) under S1500 and S150 conditions (*SI Appendix*, Fig. S7*B*). RA levels in *A. thaliana* seedlings are highly correlated with endogenous GL concentrations (41), further supporting the view that RA is synthesized predominantly from endogenous GLs even under normal growth conditions.

**Fig. 4.**
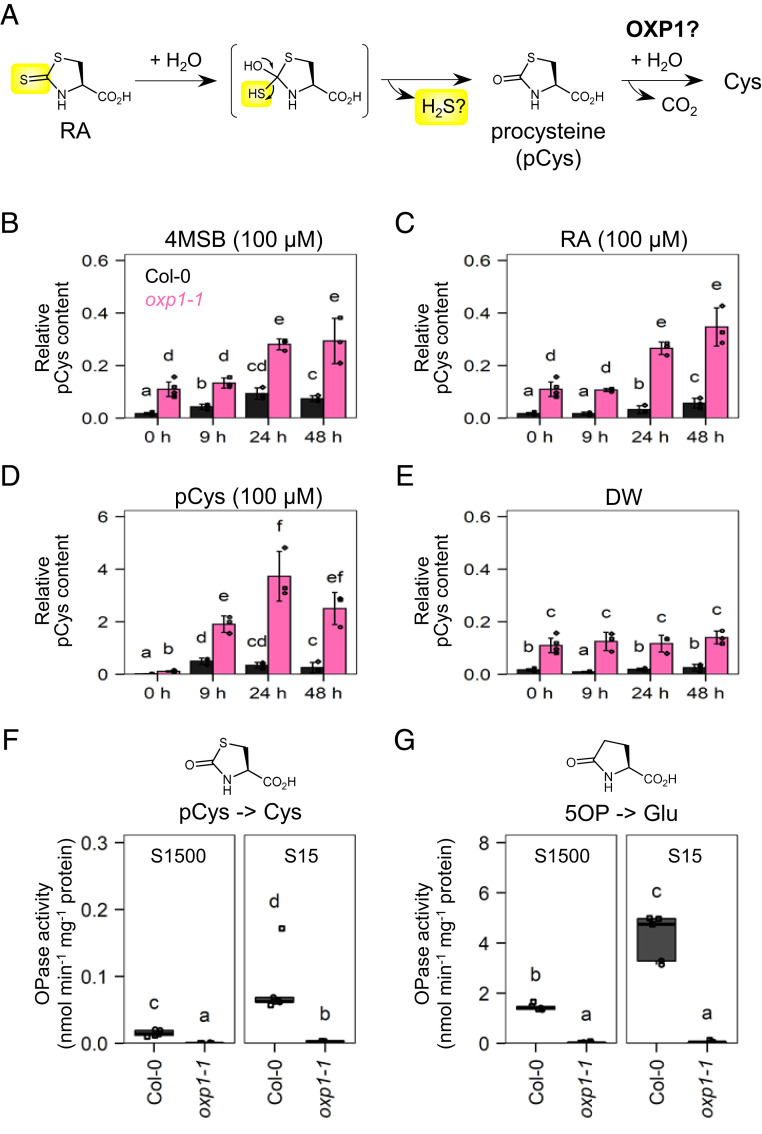
Regeneration of Cys from RA dependent on OXP1. (*A*) Potential biochemical reactions that regenerate Cys from RA coupled with release of the isothiocyanate-derived sulfur atom. pCys, procysteine. (*B*–*E*) Relative content of pCys in 8-d-old seedlings of Col-0 (dark gray) and the *oxp1-1* mutant (pink) treated with 100 μM 4MSB (*B*), RA (*C*), pCys (*D*), or DW (*E*) (solvent control) for 48 h (*n* = 3). (*F* and *G*) OPase activity in crude protein extracts from 14-d-old seedlings of Col-0 and *oxp1-1* grown at S1500 and S15 conditions (*n* = 5). (*F*) Cys production from pCys and (*G*) Glu production from 5OP. Point shapes indicate individual experimental batches. Letters indicate statistical significance corresponding to two-tailed *t* tests, as applied in Fig. 1 (*P* < 0.05). Error bars indicate SD.

Subsequently, we explored the reactions that further convert RA into primary metabolites such as Cys. The 1,3-thiazolidin-2-thione ring in RA has a potential to release the ITC-derived sulfur atom via a sulfur–oxygen exchange reaction (42), producing 1,3-thiazolidin-2-one-4-carboxylic acid (procysteine, hereafter referred to as pCys) (Fig. 4*A*). Indeed, the Col-0 seedlings accumulated pCys after 4MSB treatment in a time-dependent manner (Fig. 4*B*). pCys functions as a Cys precursor in vivo through a ring-open reaction catalyzed by 5-oxoprolinase (OPase), whose original function is Glu production from 5-oxoproline (5OP) (43). Using the *oxp1-1* mutant, which lacks the dominant OPase in *A. thaliana* (44), we evaluated pCys levels in the tissues treated with 4MSB, RA, or pCys. In addition to the higher background level (Fig. 4*E*), pCys accumulation in the mutant after such treatments was greater than that in the WT (Fig. 4 *B*–*D*). Protein extracts of the *oxp1-1* mutant incubated with pCys showed a significantly lower rate of Cys production than the WT as observed for the Glu production from 5OP, supporting that *At*OXP1 can hydrolyze pCys, as reported for OPases in other organisms (Fig. 4 *F* and *G*) (43, 45). Notably, extracts of the WT seedlings cultured at S15 condition exhibited increased OPase activity against both pCys and 5OP, which were 5.6- and 2.9-fold higher than those cultured at S1500, respectively (Fig. 4 *F* and *G*). It was less drastic or not observed in the *oxp1-1* mutant. Considering the high Cys production requirements under sulfur deficiency, the OXP1-dependent pCys hydrolysis potentially facilitates Cys regeneration from RA after GL breakdown, although the major OXP1 function is associated with GSH catabolism (see *Discussion*).

**Fig. 5.**
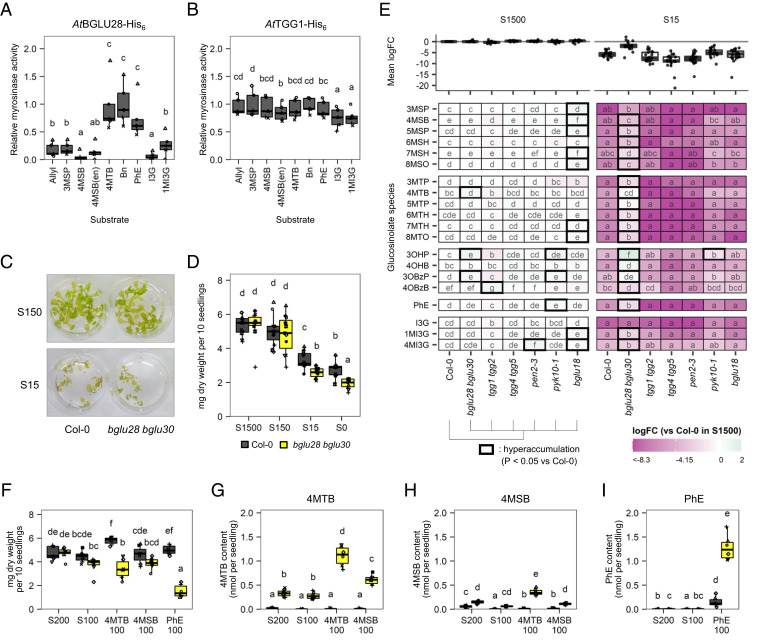
GL breakdown by BGLU28 and BGLU30 and its relevance in plant tolerance under sulfur deficiency. (*A* and *B*) Myrosinase activity in crude protein extracts from *N. benthamiana* leaves expressing *At*BGLU28-His_6_ (*A*) and *At*TGG1-His_6_ (*B*), based on the production of d-glucose in the presence of indicated GL species as a substrate (*n* = 5). Values were normalized to the mean activity of respective BGLU protein toward the GL substrate that showed the highest activity. (*C* and *D*) Growth of the *bglu28 bglu30* mutant at different sulfur concentrations. S1500, S150, S15, and S0 indicate 1,500, 150, 15, and 0 μM SO_4_^2-^, respectively. (*C*) Images of 14-d-old seedlings of WT Col-0 and *bglu28 bglu30* at S150 and S15 conditions. (*D*) Dry weights of the 14-d-old seedlings as the sum of 10 plants (*n* = 11). (*E*) Relative GL contents in 14-d-old seedlings of various *bglu* mutants grown at S1500 or S15 conditions (Col-0 and *bglu28 bglu30*, *n* = 7; *tgg1 tgg2*, *n* = 3; other *bglu* mutants, *n* = 4). The heatmap shows log_2_-scale fold changes (logFC) of each GL species relative to Col-0 in S1500. Mean logFC values of each GL species are shown as boxplots. Thick lines in the heatmap indicate significant GL accumulation compared to Col-0 within the respective growth condition. (*F*–*I*) Growth and GL contents of 14-d-old seedlings cultured with 100 μM 4MTB, 4MSB, or PhE as the sole sulfur source (*n* = 7). S200 and S100 indicate 200 and 100 μM SO_4_^2-^, respectively. (*F*) Dry weights as the sum of 10 plants. (*G*–*I*) Concentrations of 4MTB (*G*), 4MSB (*H*), and PhE (*I*) in plant tissues. Point shapes in *A*, *B*, *D*, and *F*–*I* indicate individual experimental batches. Letters indicate statistical significance corresponding to two-tailed *t* tests, as applied in Fig. 1 (*P* < 0.05). Abbreviations of GLs correspond to those listed in Table 1.

Lastly, we addressed the physiological advantages of accumulating endogenous GLs as a sulfur reservoir in *A. thaliana*. It has been suggested that BGLU28 and BGLU30 are involved in the GL degradation under sulfur deficiency (34); however, their enzymatic properties are yet to be characterized. Therefore, we first examined in vitro myrosinase activities of these BGLUs. Protein extracts of *Nicotiana benthamiana* leaves expressing *At*BGLU28 tagged with His_6_ at the *C* terminus exhibited higher rates of hydrolysis against 4MTB, benzyl glucosinolate, and PhE than those against other GLs (Fig. 5*A*). *At*BGLU30-His_6_ was not expressed under this condition, and the myrosinase activity of His_6_-GFP was at background levels, despite His_6_-GFP having the highest protein expression levels observed (*SI Appendix*, Fig. S9*A*). In contrast, the extract containing *At*TGG1-His_6_ hydrolyzed all the tested GLs at similar rates (Fig. 5*B* and *SI Appendix*, Fig. S9*B*), which is comparable with a previous finding that the leaf-abundant myrosinase could accept a broad set of substrates (46).

Physiological relevance of BGLU28 and BGLU30 in the sulfur reallocation from GLs is further elaborated. Since a recent study reported that these BGLUs play redundant roles under sulfur deficiency (34), plant growth and metabolic phenotypes of a *bglu28 bglu30* double-knockout mutant were monitored using different alleles (*SI Appendix*, Fig. S10). When the *bglu28 bglu30* seedlings were cultured under different sulfate concentrations for 14 d, their growth under S15 and S0 conditions were more severely inhibited than those of the WT (Fig. 5 *C* and *D*). The mean dry weight of the mutant seedlings at S0 was 1.93 ± 0.28 mg per 10 seedlings when compared with 2.78 ± 0.52 mg WT (Fig. 5*D*). We analyzed relative changes of 20 GL species in the seedling extracts using a high-sensitive targeted LC–MS/MS system (47). Col-0 seedlings at normal conditions mainly accumulate aliphatic GLs with a C4 or C8 side chain as well as indolic GLs (*SI Appendix*, Table S1) (41). At S15 and S0 conditions, all GL species in the WT were near the detection limit whereas 18 GLs in the *bglu28 bglu30* mutant exhibited higher abundance than those in the WT, presenting significantly altered GL profiles (Fig. 5*E* and *SI Appendix*, Fig. S11). Specifically, 4MTB and 5-methylthiopentyl glucosinolate were hardly degraded in the mutant even under S0 (*SI Appendix*, Fig. S11), which was comparable with the substrate specificity of BGLU28 toward 4MTB in vitro (Fig. 5*A*). These results above suggest that a moderate proportion of endogenous GLs is hydrolyzed by such BGLUs under sulfur deficiency, and the lower growth performance of the mutant is due to the limited access to endogenous GLs stored as a sulfur reservoir. Notably, the hyperaccumulation of GLs was not observed in other *bglu* mutants lacking known myrosinases (*tgg1 tgg2*, *tgg4 tgg5*, *pen2-3*, *pyk10-1*, and *bglu18*) under the S15 condition, indicating the specific functions of BGLU28 and BGLU30 in this phenotype (Fig. 5*E* and *SI Appendix*, Fig. S12).

The effects of the *BGLU28* and *BGLU30* mutations were further evaluated at later developmental stages using 4-wk-old plants grown in hydroponic culture (48). Although uncontrollable microenvironment under this growth condition resulted in a batch-to-batch variation among independent experiments, the *bglu28 bglu30* mutant cultured under 3 μM SO_4_^2-^ tended to exhibit poor growth performance and altered GL profiles in rosette leaves compared to the WT (*SI Appendix*, Figs. S13 and S14). However, the growth and metabolic changes in the mutant were not as striking as those observed in younger seedlings. Consequently, we hypothesized that this age-dependent difference in the *BGLU28* and *BGLU30* mutation effect is associated with different GL profiles between young seedlings and mature rosette leaves (*SI Appendix*, Table S1).

To validate this hypothesis, BGLU28 and BGLU30 utilization of GLs as sulfur sources was further evaluated in vivo by growing the seedlings with various GLs. The *bglu28 bglu30* mutant exhibited impaired growth performance when grown with 4MTB as the sole sulfur source, which was not observed in the WT (Figs. 1*D* and 5*F*). The 4MTB feeding resulted in hyperaccumulation of intact 4MTB in the mutant plant tissues, far beyond the endogenous 4MTB level in the WT under normal conditions (Fig. 5*G* and *SI Appendix*, Table S1). It implies that the mutant has significantly less access to 4MTB as a sulfur source. More drastic growth inhibition was observed when the mutant was fed with PhE (Fig. 5 *F* and *I*). Conversely, the WT and *bglu28 bglu30* mutant seedlings exhibited similar growth trends under 4MSB feeding (Fig. 5 *F* and *H*), suggesting that the moderate growth defect of the mutant fed by 4MTB is possibly because a part of the supplied 4MTB was oxidized into 4MSB (Fig. 5*H*), which can be catabolized with less influence from these BGLUs. Overall, our results demonstrate that BGLU28 (and BLU30) is a specialized myrosinase that is essential for plant tolerance of sulfur deficiency by hydrolyzing particular GL species, especially at early developmental stages; however, it is not the only BGLU responsible for sulfur reallocation from the endogenous GL pool.

## Discussion

**Fig. 6.**
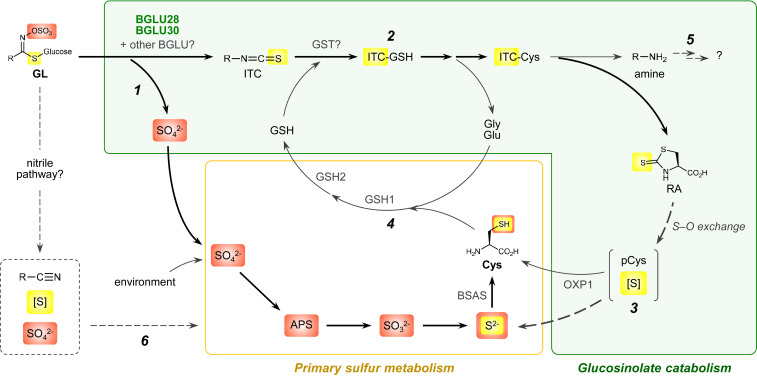
Schematic model of the sulfur reallocation pathway from GLs in *A. thaliana*. Sulfur atoms derived from sulfate and thioglucoside groups in the GL structure are highlighted in orange and yellow, respectively. Both sulfur atoms could ultimately be reintegrated into Cys biosynthesis as S^2-^. Sulfates produced by the GL hydrolysis can be directly incorporated into primary sulfur metabolism as substrates (*1*). The sulfur atom in the thioglucoside group is relayed to RA through GL catabolism including the conjugation of ITC with GSH (*2*), which is finally released from RA and then reintegrated into the primary sulfur metabolism (*3*). GSH and its amino acid components (Gly, Glu, and Cys) form a catabolic loop during ITC processing (*4*). Further processing of the amine product may also mobilize the Met-derived sulfur at the side chain of aliphatic GLs (*5*). Contribution of the nitrile pathway (*6*) remains unclear, despite its potential to release two sulfur atoms directly through the initial hydrolytic reactions. APS, adenosine-5′-phosphosulfate; BSAS, beta-substituted alanine synthase; GST, glutathione *S*-transferase.

In the present study, we depict the molecular pathway underlying mobilization of two sulfur atoms common in GL molecules through a GL breakdown process, which involves BGLU28 and BGLU30 myrosinases (Fig. 6). After hydrolysis of the thioglucosidic bond potentially coupled with SO_4_^2-^ release, the sulfur atom in the ITC group is transferred to RA via ITC–thiol conjugates and finally released from RA during Cys regeneration reactions partially dependent on OXP1. 4MSB-NH_2_ may be further modified to mobilize the Met-derived sulfur atom in the side chain. The sulfur reallocation from endogenous GL storage offers another physiological advantage in the accumulation of significant GL amounts in plant tissues, in addition to the classical chemical defense function upon tissue damage. Our results showing myrosinase activity of BGLU28, altered GL profiles in the *bglu28 bglu30* mutant at low sulfur conditions, and impaired availability of exogenous GLs as a sulfur source in the mutant support the view that BGLU28 (and possibly BGLU30) will mainly target methylthio-, hydroxy-, benzoyloxy-, and benzenic-type GLs (Fig. 5 and *SI Appendix*, Figs. S11–S14). Notably, Col-0 seeds accumulate such GL species at higher concentrations than other tissues at different developmental stages, and more than 80% of total GL content in the seeds consists of these GLs (35). Therefore, these BGLUs are potentially crucial for exploiting the GL pool in seeds as a sulfur reservoir during early development. Conversely, lower effect of the *BGLU28* and *BGLU30* mutations on the performance of sulfur-starved 4-wk-old plants can be explained by the fact that the major GL species in rosette leaves are methylsulfinyl-type ones (*SI Appendix*, Table S1) (35). In addition, considering major proportions of methylsulfinyl-type and indolic GLs disappeared under sulfur deficiency regardless of the genotypes tested (Fig. 5*E*), there may be other myrosinases responsible for hydrolysis of such GL species. Although transcriptomic analyses did not suggest any other *BGLU* up-regulated under sulfur deficiency (32, 33, 49), *BGLU28* to *BGLU32* in *A. thaliana* belong to the same *BGLU* subfamily, and their homologs are well conserved within the order Brassicales (22, 23), implying their function as myrosinase. Also, considering GL degradation upon sulfur deficiency is observed in a wide range of species in the Brassicaceae family (19), we predict that a similar sulfur reallocation system is conserved in the GL-producing plants.

The sulfur reallocation pathway presented here seems to be closely associated with aliphatic GLs, despite the fact that indolic GLs account for a major proportion of endogenous GLs in seedlings (41). Exogenous I3G could rescue the impaired plant growth at the S0 condition, whereas unlike the other GL classes, the corresponding amine and RA were not accumulated in the tissues (Fig. 1*D* and *SI Appendix*, Fig. S8). Given PEN2 and PYK10 myrosinases are closely linked to the metabolism of indolic GLs, which coordinates with the metabolism of a variety of indolic phytoalexins and plant immunity (27, 28, 50), the degradation of indolic GLs at low sulfur conditions may have other physiological roles than sulfur allocation. Nevertheless, the GSH-dependent breakdown of 4MSB that generates RA and amines resembles what is proposed in the PEN2-mediated breakdown of indolic GLs, whose intermediates are yet to be identified (27, 28). The results of our tracer experiments and the earlier studies potentially demonstrate a general principle based on which ITCs with diverse side chains are processed in planta in a manner dependent on conjugation with GSH. The distinct GL bioactivities may be controlled under a conserved catabolic system linked with diverse myrosinases. Notably, 4MSB fed into humans is also processed in a similar manner: SFN induces phase II antioxidant enzymes including glutathione *S*-transferases and the SFN-GSH conjugate is hydrolyzed stepwise, whereas the major end product excreted into urine is the conjugate with *N*-acetylcysteine, which is not detected in our metabolomics data (*SI Appendix*, Fig. S15 and Table S2) (51). Therefore, cyclization of ITC-Cys would occur exclusively in GL-producing plants and would be essential for the catabolic loop between GSH and amino acid components during ITC processing.

The potential relevance of OXP1 in GL catabolism is also of interest. In *A. thaliana*, sulfur starvation facilitates 5OP accumulation through GSH hydrolysis to recruit Cys, which is catalyzed by a low sulfur-inducible gamma-glutamylcyclotransferase (52). In such a context, the activation of OXP1 in plants grown at S15 would avoid potential Glu loss, although it may not directly affect endogenous sulfur content. In contrast, the enhanced pCys hydrolysis potentially facilitates efficient Cys regeneration from RA produced via GL breakdown. Taken together, OXP1 may be involved in multiple physiological processes, which are both crucial for sulfur recruitment from endogenous metabolite pools. Similarly, phytochelatin synthase is involved in both phytochelatin biosynthesis from GSH and catabolism of indolic GLs (53). Our results further highlight the close interconnection between primary and specialized metabolism that regulates endogenous sulfur contents in response to environmental conditions.

In addition to BGLUs and OXP1, characterizing the enzymes responsible for the sulfur reallocation pathway from GLs remains challenging. Regarding the conversion from RA to pCys, a similar *S*–*O* exchange reaction has been reported in the order Brassicales, which transforms a 1,3-oxazolidine-2-thione ring in GL hydrolytic products to 1,3-oxazolidin-2-one (*SI Appendix*, Fig. S16) (54). This potentially enzymatic reaction could facilitate the unraveling of the mechanism underlying sulfur release from the 1,3-thazolidine-2-thione ring of RA, which might be involved in the growth inhibitory activity of RA against plants (55). Moreover, considering the diverse reactions underlying GL breakdown (14), there may be alternative routes other than the ITC-dependent pathway validated in the present study. The nitrile pathway could release two sulfur atoms directly through the initial hydrolytic reactions (Fig. 6), and the genes involved in nitrile formation are well expressed in young seedlings and under sulfur deficiency (18, 21). Our targeted and untargeted metabolite analyses detected several unknown metabolites potentially involved in the catabolic GL processing in plant tissues (*SI Appendix*, Fig. S17 and Tables S2 and S3 and Dataset S1), although they were not adequate to further expand the reactions depicted in Fig. 6, including the relevance of the nitrile pathway and the subsequent modifications after 4MSB-NH_2_. Overall, further studies are required for a comprehensive understanding of sulfur reallocation through GL catabolism.

Specialized metabolic pathways in plants have long been considered one-directional routes to synthesize bioactive end products. The GL breakdown leading to sulfur reallocation described here demonstrates a physiological advantage of the retrograde pathways that revert such “end” products to primary metabolites for the supply of starved essential nutrients. Given the dynamic fluctuation of endogenous compound concentrations in response to environmental changes, a broad range of specialized metabolites may possess similar regulatory systems to interconnect different metabolite classes, including primary metabolites and plant hormones (6). Advancing our knowledge on catabolic pathways, in addition to biosynthetic regulation, would not only facilitate rational control of the balance between nutrient storage and defensive functions of specialized metabolites but also the engineering of the appropriate levels of compounds beneficial for humans in crop plants.

## Materials and Methods

### Plant Materials.

*A. thaliana* Columbia-0 (Col-0) accession was used as the control. The following mutant lines in the Col-0 genetic background were kind gifts: *bglu28* (SALK_067086), *bglu30* (SALK_029737), *tgg4* (SALK_070446C), *tgg5* (SALK_114084C), *pen2-3* (CS66946), and *pad2-1* (CS3804) from the *Arabidopsis* Biological Resource Center (Ohio State University, Columbus, OH); *cyp79b2 cyp79b3 myb28 myb29* from B. A. Halkier (Dept. of Plant and Environmental Sciences, University of Copenhagen, Frederiksberg C, Denmark); *tgg1 tgg2* from G. Jander (Boyce Thompson Institute, Ithaca, NY); *pyk10-1* (CS69080) and *bglu18* (SALK_075731C) from I. Hara-Nishimura (Faculty of Science and Engineering, Konan University, Kobe, Japan). The *bglu28 bglu30* and *tgg4 tgg5* double-knockout mutants were generated by crossing the corresponding single mutants. Genotypes of the transfer DNA insertion mutants were confirmed by a PCR using the primers listed in *SI Appendix*, Table S4. Phenotypes of *pen2-3* and *pad2-1*, the ethyl methanesulfonate mutants, were confirmed by endogenous indolic GL concentrations after flg22 treatment (27) and endogenous GSH concentration (39), respectively.

### Chemicals.

Chemicals used in the present study were purchased from the following suppliers and used without further purification: indol-3-ylmethyl glucosinolate and 1-methoxyindol-3-ylmethyl glucosinolate from EXTRASYNTHESE; l-cysteine from FUJIFILM Wako Chemical; PhE and (d,l)-sulforaphane from LKT Laboratories; GSH from Nacalai Tesque; 4MSB from Nagara Science; allyl glucosinolate, gamma-l-glutamyl-l-cysteine, and RA from Sigma-Aldrich; 3-methylsulfinyl-*n*-propyl glucosinolate, 4-methylsulfinyl-3-butenyl glucosinolate, and 4-methylthio-*n*-butyl glucosinolate from PhytoLab; benzyl glucosinolate and pCys from Tokyo Chemical Industry; and buthionine sulfoximine and 4-methylsulfinyl-*n*-butylamine from Toronto Research Chemicals. Isotope-labeled 4MSB (4MSB-^34^*S* and 4MSB-*d*_5_) was chemically synthesized as described in *SI Appendix*.

### Culture Conditions.

*A. thaliana* plants were grown at 22 °C in an environmentally controlled incubation room under a 16-h light/8-h dark cycle. The nutrient solutions in which MgSO_4_ is the sole sulfur source were prepared based on Murashige–Skoog medium (56). MnSO_4_, ZnSO_4_, CuSO_4_, and FeSO_4_/Na_2_EDTA (ethylenediaminetetraacetic acid) in the original recipe were replaced with the same metal concentrations of MnCl_2_, ZnCl_2_, CuCl_2_, and NaFe EDTA ⋅ 3H_2_O, respectively. The S1500 medium contained MgSO_4_ at 1,500 μM while the S0 medium contained MgCl_2_ instead of MgSO_4_ at the same concentration. Media containing different sulfate concentrations were prepared by mixing S1500 and S0 accordingly. Detailed conditions for each experiment are described below.

### Cultivation with Various Sulfur Sources.

After surface sterilization and 3-d vernalization, *A. thaliana* seeds were sown into 12-well microtiter plates (10 seeds in each well) with 2 mL nutrient solution at different SO_4_^2-^ concentrations. For cultivation with GL as the sole sulfur source, GL dissolved in sterile DW at 20 mM was added to S0 with final concentrations of 100 or 150 μM. Sample allocation in a plate was fixed, but the orientation and the position of plates in the climate chamber were always fully randomized (the same applies hereafter). Seedlings were incubated for 14 d and then frozen in a 1.5-mL tube after washing with 3 mL sterile DW three times and stored at −80 °C until use. Lyophilized whole-plant materials were weighed and subjected to the metabolite analyses. The number of experimental batches can be found in the corresponding figure legends.

### Treatment with Chemicals.

After surface sterilization and 3-d vernalization, ∼200 *A. thaliana* seeds were precultured in a 90 × ϕ20 mm Petri dish (SH90-20, IWAKI) with 25 mL liquid S1500 medium. The 7-day-old seedlings of Col-0 precultured in S1500 medium were transferred to 12-well microtiter plates (10 plants in each well) with 1 mL fresh S1500 medium to minimize differences in total plant mass in each well. On the following day, samples were treated with chemicals as follows. Each of the following experiments were repeated three times.

To treat the intact or homogenized seedlings (Fig. 3*A*), 8-d-old seedlings of Col-0 were placed in 2.0-mL tubes containing 5-mm zirconia beads with the culture media and then homogenized using a Mixer Mill MM300 (Retsch) for 3 min at 20 Hz and 4 °C. After putting the homogenates back into the 12-well microtiter plates, 4MSB was added to the wells containing intact seedlings, homogenates, or S1500 medium only with a final concentration of 100 μM. Culture media (20 μL) were collected at 0.1, 3, 9, 24, and 48 h and subjected to the metabolite analyses.

For the untargeted metabolomics (Fig. 3*B*), 8-d-old seedlings of Col-0 were treated with 200 μM 4MSB, or 4MSB-*d*_5_, or solvent control (sterile DW) for 3, 9, 24, or 48 h. After treatment, seedlings were washed followed by measurement of the fresh weights and then frozen in 2.0-mL tubes containing 5-mm zirconia beads and stored at −80 °C until use. Fresh frozen samples were subjected to the metabolite analyses.

To treat the *oxp1-1* mutant (Fig. 4), 8-d-old seedlings of Col-0 and *oxp1-1* were treated with 100 μM 4MSB, RA, pCys, or solvent control (sterile DW) for 0, 9, 24, or 48 h. After treatment, seedlings were washed, frozen, and stored at −80 °C until use. Lyophilized whole-plant materials were weighed and subjected to the metabolite analyses.

For the treatments under inhibited GSH biosynthesis (*SI Appendix*, Fig. S6), three groups (Col-0, *pad2-1*, and Col-0 with 1 mM buthionine sulfoximine) were prepared when 7-d-old seedlings were transferred to 12-well microplates. Seedlings were treated with 100 μM 4MSB for 0, 9, 24, or 48 h. After treatment, seedlings were washed, frozen, and stored at −80 °C until use. Culture media (20 μL) were also collected in 1.5-mL tubes at 0, 3, 9, 24, 33, and 48 h and stored at −30 °C until use. After weighing the lyophilized whole-plant materials, plant tissues as well as the culture media were subjected to the metabolite analyses.

### Quantitation of Thiol Metabolites.

Cys and GSH in the extract were derivatized with monobromobimane as previously reported (57, 58), followed by high-performance liquid chromatography (Shimadzu) with the Shim-pac FC-ODS column (150 × 4.6 mm, Shimadzu).

### Quantification of Selected Metabolites Using LC–MS/MS.

Metabolite analysis of the plant extracts using LC–MS/MS was performed as previously reported (47) with some modifications. Detailed conditions for sample preparation and data acquisition are described in *SI Appendix*. Retention time and *m/z* values of precursor and product ions for each metabolite are listed in *SI Appendix*, Table S5.

Peak intensities of the detected metabolites were calculated as the area under the curve using LabSolutions (Shimadzu) and exported as a numeric matrix on Excel (Microsoft). After the missing values were set to 10 (noise level), intensities of individual metabolites were divided by that of the internal standard: lidocaine (precursor: *m/z* 235, product: *m/z* 86) for positive ions; 10-camphorsulfonic acid (precursor: *m/z* 231, product: *m/z* 80) for negative ions. It should be noted that such values do not indicate their absolute contents but simply reflect sensitivity of the metabolites of interest in the analytical system.

Absolute GL concentrations in the control groups were quantified based on the standard curves of authentic 4MSB, 4MTB, PhE, and I3G at physiological concentrations (*SI Appendix*, Fig. S18), which were applied to estimate the amounts of the corresponding subclasses, as previously reported (34).

### Untargeted Metabolomics Using LC–QTOF/MS.

Metabolomics analyses of the plant extracts were performed using a high-resolution LC–QTOF/MS as previously reported (38), with some modifications. Detailed conditions for sample preparation and data acquisition are described in *SI Appendix*.

The data matrix was aligned using MakerLynx (Waters). Peak intensities recorded at less than 500 were transposed to 500 (noise level). Intensities of individual metabolites were divided by that of the internal standard: lidocaine (*m/z* 235.1805) for positive ions; 10-camphorsulfonic acid (*m/z* 231.0697) for negative ions. Chemical assignment for each record was performed based on the KNApSAcK database (http://www.knapsackfamily.com/KNApSAcK_Family/, keyword: *Arabidopsis*) (59) and references (60–62). Values of *m/z* were set for precursor ions ([M+H]^+^ or [M−H]^−^). The matching value of the reference was searched for with a tolerance of 0.01 Da. The processed data matrix can be found in Dataset S1. Deuterium-labeled metabolites were screened using R v3.6.3 (http://www.R-project.org) based on the following criteria (see also *SI Appendix*, Fig. S3):

1. The mean signal intensity at any experimental group is more than twice the noise level.
2. The record has a pair signal showing the mass difference corresponding to the incorporation of two, three, five, or 10 deuterium atoms (2.0126, 3.0189, 5.0314, or 10.0628 mass unit, respectively). The tolerance of mass difference was set at ± 20 ppm of the query *m/z* value.
3. A pair of records has the same retention time (tolerance: ± 0.05 min).
4. The “unlabeled” and “labeled” records show the highest intensities in the samples treated with 4MSB and with 4MSB-*d*_5_, respectively.

After screening, 42 and 13 pairs of records were extracted from the data in positive and negative ion modes, respectively. Pairs potentially belonging to the same metabolite (e.g., [M+H]^+^, [M−H]^−^, and [2M+H]^+^ ions) were organized into a pair based on the highest signal intensity. Finally, 26 record pairs were obtained as the candidates of labeled metabolites (*SI Appendix*, Table S2). Metabolites whose levels were influenced by both 4MSB treatments in the same manner regardless of the deuterium label were screened with criteria 1 and the following two criteria:

5. The highest or lowest intensities belong to the samples treated with 4MSB or with 4MSB-*d*_5_.
6. The Spearman’s rank correlation coefficient between samples treated with 4MSB and with 4MSB-*d*_5_ is higher than 0.4, whereas that between samples treated with DW and with 4MSB and between samples treated with DW and with 4MSB-*d*_5_ are both 0.2 or below.

The screening extracted 50 and 31 records from the data in positive and negative ion modes, respectively, which were organized into 67 records, including 18 records with metabolite annotation based on the KNApSAcK database (*SI Appendix*, Table S3). MS/MS-based structural analyses of the metabolites shown in Fig. 3*B* were performed as described in the *SI Appendix*.

### Analysis of the Authentic Standards.

Physicochemical properties of the potential intermediates inferred in the metabolomics analyses were compared with those of authentic standards, as described in *SI Appendix*.

### OPase Assay.

The OPase activity assay was performed as previously reported (44), with some modifications, as described in *SI Appendix*.

### Myrosinase Assay.

Myrosinase assays were performed as previously reported (63), using proteins transiently expressed in *N. benthamiana* (64, 65), with some modifications, as described in *SI Appendix*.

### Hydroponic Culture.

Hydroponic culture was performed as previously reported (48), with some modifications, as described in *SI Appendix*.

### Statistical Analysis.

Data were fitted with a linear model (LM) or a linear mixed model (LMM) with the batch as a fixed or a random factor, respectively, depending on the dataset. The LM analysis was performed by canonical ANOVA and post hoc Tukey's tests. The LMM analysis was performed in R using the *lmer* function in lme4 package using the following formula:

value ∼ condition – 1 + (1|batch),

where “condition” includes sulfur concentrations, time points, genotypes, or their combinations, depending on the experimental setup. We only accounted for the interaction of factors, as we were only interested in the interaction and to facilitate pairwise comparison, which was the major aim of the statistical approach. Log- or square root–transformation were applied to achieve normality, whenever necessary. The accuracy of fitting was checked based on Q-Q plots as well as residual plots, and the data transformation that provided the optimal fitting accuracy was selected in a post hoc manner. Pairwise comparison was preformed using the Student’s *t* test using the covariance matrix and the estimates provided by the *lmer* function, followed by a correction for multiple comparison using Benjamini–Hochberg’s method.

## Supplementary Material

Supplementary File

Supplementary File

## Data Availability

All raw data and corresponding scripts, as well as intermediate data are available at GitHub (https://github.com/rtnakano1984/Sugiyama_PNAS).
