# Supplementary material for: Retrograde sulfur flow from glucosinolates to cysteine in Arabidopsis thaliana
Source: Proc Natl Acad Sci U S A. 2021 May 25;118(22):e2017890118. doi: 10.1073/pnas.2017890118 (PMC8179156; doi:10.1073/pnas.2017890118)
Supplement: Supplementary File [file pnas.2017890118.sapp.pdf]

Supplementary Information for

Retrograde sulfur flow from glucosinolates to cysteine in *Arabidopsis thaliana*

Ryosuke Sugiyama*, Rui Li, Ayuko Kuwahara, Ryo Nakabayashi, Naoyuki Sotta, Tetsuya Mori, Takehiro Ito, Naoko Ohkama-Ohtsu, Toru Fujiwara, Kazuki Saito, Ryohei Thomas Nakano, Paweł Bednarek and Masami Yokota Hirai*

*To whom correspondence may be addressed. Emails: phars@nus.edu.sg (R.S.) or masami.hirai@riken.jp (M.Y.H.)

This PDF file includes:

Supplementary text
Figures S1 to S18
Schemes S1 to S2
Tables S1 to S5
NMR spectroscopic data
SI References

Other supplementary materials for this manuscript include the following:

Dataset S1

Supplementary Information Text

Synthesis of isotope-labeled 4-methylsulfinyl-*n*-butyl glucosinolate

General. Isotopically labeled glucosinolates were synthesized based on the previous reports (1, 2). All reactions were carried out under nitrogen atmosphere with dehydrated solvents under anhydrous conditions, unless otherwise noted. All solvents and reagents were obtained from commercial suppliers and used without further purification. Dimethyl sulfoxide-d_6 (99.9% D) and thiourea-^{34}S (90% ^{34}S) were purchased from Sigma-Aldrich and Icon Isotopes, respectively. Reactions were monitored by thin-layer chromatography (TLC) on silica gel plates (FUJIFILM Wako Pure Chemical, Silicagel 70 F254 TLC Plate-Wako). Column chromatography was performed on Silica gel 60 (Merck, 40-63 µm). High-resolution MS data were recorded on LC-QTOF/MS (LC, Waters Acquity UPLC System; MS, Waters Xevo G2 Q-Tof). ^{1}H and ^{13}C NMR spectra were recorded on a Bruker AVANCE III HD 600 spectrometer equipped with a 5 mm DCH CryoProbe™. In the NMR spectra, chemical shift values (in ppm) are shown relative to the solvents: δ_H 7.26, 3.31 and 0.00 ppm and δ_C 77.16, 49.00 and 0.00 ppm are used for $CDCl_3$, CD_3OD and D_2O with sodium trimethylsilylpropanesulfonate, respectively. The following abbreviations are used to explain the multiplicities: s = singlet, d = doublet, t = triplet, q = quartet, m = multiplet, br = broad.

2,3,4,6-Tetra-*O*-acetyl-β-D-glucopyranosyl thiol (S1). To a stirred solution of 1-bromo-2,3,4,6-tetra-*O*-acetyl-β-D-glucopyranose (411 mg, 1.0 mmol) in dry acetone (4 mL) was added thiourea (100 mg, 1.3 mmol). After 3 h of reflux under a nitrogen atmosphere, the mixture was cooled and concentrated in vacuo. Trituration of the syrupy residue with *n*-heptane gave the isothiouronium bromide as a colorless amorphous powder. The crude product was dissolved in CH_2Cl_2 (4 mL) and a solution of $Na_2S_2O_5$ (380 mg, 2.0 mmol) in water (4 mL) was added, and then the mixture was maintained at reflux under a nitrogen atmosphere for 1 h. After cooling, the organic layer was separated and washed with water and brine, dried over Na_2SO_4, filtered, and concentrated under reduced pressure to give a residue. Pure **S1** was obtained by flash column chromatography on silica gel eluting with *n*-heptane/EtOAc (2:1) as a white solid (255 mg, 70%). HR-ESI-MS *m/z* calcd for $C_{14}H_{21}O_9S$ $[M+Na]^+$ 387.0720, found 387.0725; ^{1}H NMR (600 MHz, $CDCl_3$) δ_H 5.19 (1H, t), 5.10 (1H, t), 4.97 (1H, t), 4.54 (1H, t), 4.25 (1H, dd), 4.13 (1H, dd), 3.72 (1H, ddd), 2.31 (1H, d), 2.09 (3H, s), 2.08 (3H, s), 2.02 (3H, s) and 2.01 (3H, s) ppm; ^{13}C NMR (150 MHz, $CDCl_3$) δ_C 170.8, 170.3, 169.8, 169.5, 78.9, 76.5, 73.72, 73.69, 68.3, 62.2, 20.90, 20.87, 20.73 and 20.71 ppm.

2,3,4,6-Tetra-*O*-acetyl-β-D-glucopyranosyl thiol-^{34}S (S1s). 1-Bromo-2,3,4,6-tetra-*O*-acetyl-β-D-glucopyranose (479 mg, 1.2 mmol) and thiourea-^{34}S (100 mg, 1.3 mmol) were subject to the procedure as described for **S1**, to yield **S1s** (262 mg, 61%). HR-ESI-MS *m/z* calcd for $C_{14}H_{21}O_9{}^{34}S$ $[M+Na]^+$ 389.0678, found 389.0677; ^{1}H NMR (600 MHz, $CDCl_3$) δ_H 5.19 (1H, t), 5.10 (1H, t), 4.97

(1H, t), 4.54 (1H, t), 4.25 (1H, dd), 4.13 (1H, dd), 3.72 (1H, ddd), 2.31 (1H, d), 2.10 (3H, s), 2.08 (3H, s), 2.03 (3H, s) and 2.01 (3H, s) ppm; ^{13}C NMR (150 MHz, $CDCl_3$) δ_C 170.8, 170.3, 169.8, 169.5, 78.9, 76.5, 73.72, 73.69, 68.3, 62.2, 20.90, 20.88, 20.73 and 20.71 ppm.

2-(4-(Methylsulfinyl)butyl)-1,3-dioxolane (S2). To NaH powder (60% dispersion in mineral oil, 450 mg) in a 15 mL test tube was added dry DMSO (4.5 mL) dropwise. The suspension was stirred at 60 °C for 3 h under nitrogen flow, followed by centrifugation at 2,000 rpm for 3 min. 3.6 mL of the supernatant as a 2.5 M solution of sodium methylsulfinylmethylide (9.0 mmol) was added dropwise into a solution of 2-(3-bromopropyl)-1,3-dioxolane (406 μL, 3.0 mmol) in dry DMSO (1 mL). After stirred at rt for 1 h, the mixture was quenched by saturated aq. NH_4Cl and extracted by $CHCl_3$ three times. The combined organic layer was washed with water and brine, dried over Na_2SO_4, filtered, and concentrated under reduced pressure to give a residue. Pure **S2** was obtained by flash column chromatography on silica gel eluting with $CHCl_3$/MeOH (25:1) as a colorless oil (546 mg, 95%). HR-ESI-MS *m/z* calcd for $C_8H_{17}O_3S$ $[M+H]^+$ 193.0893, found 193.0898; ^{1}H NMR (600 MHz, $CDCl_3$) δ_H 4.86 (1H, t), 3.97–3.83 (4H, m), 2.77–2.64 (2H, m), 2.56 (3H, s) and 1.85–1.52 (6H, m) ppm; ^{13}C NMR (150 MHz, $CDCl_3$) δ_C 104.2, 65.0, 54.7, 38.7, 33.4, 23.3 and 22.6 ppm.

2-(4-((Methyl-*d*$_3$)sulfinyl)butyl-4,4-*d*$_2$)-1,3-dioxolane (S2d). 2-(3-Bromopropyl)-1,3-dioxolane (474 μL, 3.5 mmol) and 4.2 mL of a 2.5 M solution of sodium methylsulfinylmethylide-*d*$_5$ (10.5 mmol) prepared from NaH (60% dispersion in mineral oil, 500 mg) and DMSO-*d*$_6$ (5 mL) were subject to the procedure as described for **S2**, to yield **S2d** (588 mg, 87%). HR-ESI-MS *m/z* calcd for $C_8H_{12}D_5O_3S$ $[M+H]^+$ 198.1207, found 198.1213; ^{1}H NMR (600 MHz, $CDCl_3$) δ_H 4.86 (1H, t), 3.97–3.83 (4H, m) and 1.84–1.52 (6H, m) ppm; ^{13}C NMR (150 MHz, $CDCl_3$) δ_C 104.2, 65.0, 33.4, 23.3 and 22.4 ppm.

5-(Methylsulfinyl)pentanal oxime (S3). To a stirred solution of **S2** (239 mg, 1.2 mmol) in 1 M HCl/MeOH (1:4, 5 mL) was added hydroxylamine hydrochloride (432 mg, 6.2 mmol). The mixture was stirred at rt for 12 h. The reaction mixture was concentrated under reduced pressure and then the residue was dissolved in water and extracted with $CHCl_3$ three times. The combined organic layers were washed with brine, dried over Na_2SO_4, and concentrated under reduced pressure. The oxime **S3** as a mixture of *E* and *Z* isomers was obtained by flash column chromatography eluting with $CHCl_3$/MeOH (20:1) as a colorless oil (172 mg, 85%). HR-ESI-MS *m/z* calcd for $C_6H_{14}NO_2S$ $[M+H]^+$ 164.0470, found 164.0742; ^{1}H NMR (600 MHz, $CDCl_3$) δ_H 7.92/7.61 (1H, s), 7.42/6.72 (1H, t), 2.80–2.65 (2H, m), 2.58 (3H, s), 2.44 (1H, m), 2.27 (1H, m), 1.87–1.80 (2H, m) and 1.75–1.62 (2H, m) ppm; ^{13}C NMR (150 MHz, $CDCl_3$) δ_C 151.8/151.1, 54.3/54.2, 38.7, 29.2/25.7, 25.4/24.5 and 22.4/22.2 ppm.

5-((Methyl-*d*$_3$)sulfinyl)pentanal oxime-5,5-*d*$_2$ (S3d). Compound **S2d** (224 mg, 1.1 mmol) and hydroxylamine hydrochloride (401 mg, 5.8 mmol) were subject to the procedure as described for **S3**, to yield **S3d** (115 mg, 60%). HR-ESI-MS *m/z* calcd for $C_6H_9D_5NO_2S$ $[M+H]^+$ 169.1054, found 169.1056; ^1H NMR (600 MHz, $CDCl_3$) δ_H 7.68/7.38 (1H, s), 7.43/6.72 (1H, t), 2.44 (1H, m), 2.27 (1H, m), 1.85–1.80 (2H, m) and 1.75–1.60 (2H, m) ppm; ^{13}C NMR (150 MHz, $CDCl_3$) δ_C 151.9/151.2, 29.2/25.6, 25.3/24.5 and 22.2/22.0 ppm.

***S*-(2,3,4,6-Tetra-*O*-acetyl-β-D-glucopyranosyl)-(5-(methylsulfinyl)pentyl)thiohydroximate (S4).** To a suspension of **S3** (172 mg, 1.1 mmol) in CH_2Cl_2 (2 mL) was added pyridine (93.3 μL, 1.2 mmol), then *N*-chlorosuccinimide (155 mg, 1.2 mmol). The mixture was stirred for 2 h at rt under nitrogen atmosphere, then thiol **S1** (344 mg, 0.95 mmol) dissolved in CH_2Cl_2 (1 mL) was added dropwise. The resulting mixture was treated with Et_3N (524 μL, 3.8 mmol) and stirred for 1 h at rt under nitrogen atmosphere then acidified with aqueous 2 M HCl (7 mL). After 10 min, the aqueous phase was extracted with $CHCl_3$ three times and the combined organic layers were dried over Na_2SO_4, filtered, and the filtrate was concentrated under reduced pressure. The compound **S4** was obtained by flash column chromatography eluting with $CHCl_3$/MeOH (40:1) as a colorless foam (188 mg, 38%). HR-ESI-MS *m/z* calcd for $C_{20}H_{32}NO_{11}S_2$ $[M+H]^+$ 526.1411, found 526.1413; ^1H NMR (600 MHz, $CDCl_3$) δ_H 5.30–5.02 (4H, m), 4.22–4.13 (2H, m), 3.79–3.70 (1H, m), 2.82–2.66 (2H, m), 2.65–2.51 (2H, m), 2.59 (3H, s), 2.08 (3H, s), 2.05 (3H, s), 2.04 (3H, s), 2.01 (3H, s) and 1.95–1.76 (4H, m) ppm; ^{13}C NMR (150 MHz, $CDCl_3$) δ_C 170.7, 170.3, 169.5, 169.3, 150.7, 80.1, 76.1, 73.9, 70.4, 68.3, 62.3, 54.2, 38.7, 32.4, 25.9, 22.2, 20.9, 20.8 and 20.7 (2C) ppm.

***S*-(2,3,4,6-Tetra-*O*-acetyl-β-D-glucopyranosyl)-(5-(methylsulfinyl)pentyl)thiohydroximate-^{34}*S* (S4s).** Compound **S3** (51.5 mg, 315 μmol), pyridine (27.9 μL, 347 μmol), *N*-chlorosuccinimide (46.3 mg, 315 μmol), thiol **S1s** (53.0 mg, 145 μmol) and Et_3N (80.4 μL, 580 μmol) were subject to the procedure as described for **S4**, to yield **S4s** (40.7 mg, 53%). HR-ESI-MS *m/z* calcd for $C_{20}H_{32}NO_{11}S^{34}S$ $[M+H]^+$ 528.1369, found 528.1392; ^1H NMR (600 MHz, $CDCl_3$) δ_H 5.30–5.02 (4H, m), 4.22–4.12 (2H, m), 3.79–3.74 (1H, m), 2.82–2.66 (2H, m), 2.65–2.51 (2H, m), 2.59 (3H, s), 2.08 (3H, s), 2.05 (3H, s), 2.04 (3H, s), 2.01 (3H, s) and 1.93–1.77 (4H, m) ppm; ^{13}C NMR (150 MHz, $CDCl_3$) δ_C 170.7, 170.3, 169.5, 169.3, 150.7, 80.1, 76.0, 73.9, 70.4, 68.3, 62.3, 54.2, 38.7, 32.4, 25.9, 22.2, 20.9, 20.8 and 20.7 (2C) ppm.

***S*-(2,3,4,6-Tetra-*O*-acetyl-β-D-glucopyranosyl)-(5-((methyl-*d*$_3$)sulfinyl)pentyl-5,5-*d*$_2$)thiohydroximate (S4d).** Compound **S3d** (115 mg, 0.65 mmol), pyridine (60.3 μL, 0.75 mmol), *N*-chlorosuccinimide (100 mg, 0.75 mmol), thiol **S1** (223 mg, 0.61 mmol) and Et_3N (339 μL, 2.5 mmol) were subject to the procedure as described for **S4**, to yield **S4d** (183 mg, 51%). HR-ESI-MS *m/z* calcd for $C_{20}H_{27}D_5NO_{11}S_2$ $[M+H]^+$ 531.1725, found 531.1723; ^1H NMR (600 MHz, $CDCl_3$) δ_H

5.30–5.02 (4H, m), 4.22–4.12 (2H, m), 3.79–3.74 (1H, m), 2.65–2.51 (2H, m), 2.08 (3H, s), 2.05 (3H, s), 2.04 (3H, s), 2.01 (3H, s) and 1.93–1.77 (4H, m) ppm; ^{13}C NMR (150 MHz, $CDCl_3$) δ_C 170.7, 170.3, 169.5, 169.3, 150.6, 80.1, 76.1, 73.9, 70.4, 68.3, 62.3, 32.4, 25.9, 22.2, 20.9, 20.8 and 20.7 (2C) ppm.

4-Methylsulfinyl-*n*-butyl glucosinolate tetraacetate potassium salt (S5). To a stirred solution of chlorosulfuric acid (47.5 μL, 713 μmol) in dry CH_2Cl_2 (0.5 mL) was added pyridine (3 mL) dropwise at -20 °C under nitrogen flow. After 15 min, a solution of **S4** (35.9 mg, 71.3 μmol) in dry pyridine (1 mL) was slowly added into the pyridine-SO_3 complex suspension. After stirring at rt under nitrogen atmosphere for 24 h, the reaction was quenched by 500 μL of aqueous 2 M $KHCO_3$ and stirred for 1 h, followed by concentration under reduced pressure. The residue was dissolved in water and extracted with $CHCl_3$ and then MeOH/$CHCl_3$ (1:4). The combined organic layer was dried over Na_2SO_4, filtered, and concentrated under reduced pressure. To remove excess pyridine, the mixture was co-distilled several times with toluene. The compound **S5** was obtained by flash column chromatography eluting with $CHCl_3$/MeOH (8:1) as a white powder (14.7 mg, 32%). HR-ESI-MS *m/z* calcd for $C_{20}H_{30}NO_{14}S_3$ $[M-K]^-$ 604.0834, found 604.0829; ^{1}H NMR (600 MHz, CD_3OD) δ_H 5.41–5.36 (2H, m), 5.06 (1H, t), 4.98 (1H, t), 4.25–4.15 (2H, m), 4.06–3.99 (1H, m), 2.97–2.79 (2H, m), 2.77–2.72 (2H, m), 2.65 (3H, s), 2.06 (3H, s), 2.03 (3H, s), 2.02 (3H, s), 1.98 (3H, s) and 1.95–1.81 (4H, m) ppm; ^{13}C NMR (150 MHz, CD_3OD) δ_C 172.2, 171.5, 171.2, 170.9, 158.4, 80.8, 76.7, 75.1, 71.4, 69.5, 63.3, 54.3, 38.1, 33.0, 26.8, 22.8, 20.7 and 20.5 (3C) ppm.

4-Methylsulfinyl-*n*-butyl glucosinolate-*thiohydroximate*-^{34}*S* tetraacetate potassium salt (S5s). Compound **S4s** (37.7 mg, 71.5 μmol), chlorosulfuric acid (95.2 μL, 1.4 mmol) and pyridine (4 mL) were subject to the procedure as described for **S5**, to yield **S5s** (16.1 mg, 35%). HR-ESI-MS *m/z* calcd for $C_{20}H_{30}NO_{14}S_2{}^{34}S$ $[M-K]^-$ 606.0792, found 606.0798; ^{1}H NMR (600 MHz, CD_3OD) δ_H 5.42–5.34 (2H, m), 5.06 (1H, t), 4.99 (1H, t), 4.24–4.16 (2H, m), 4.06–4.02 (1H, m), 2.97–2.81 (2H, m), 2.77–2.72 (2H, m), 2.65 (3H, s), 2.07 (3H, s), 2.03 (3H, s), 2.02 (3H, s), 1.98 (3H, s) and 1.95–1.85 (4H, m) ppm; ^{13}C NMR (150 MHz, CD_3OD) δ_C 172.2, 171.5, 171.2, 170.9, 158.4, 80.8, 76.7, 75.1, 71.4, 69.5, 63.3, 54.3, 38.1, 33.0, 26.8, 22.7, 20.7 and 20.5 (3C) ppm.

4-(Methyl-*d*$_3$)sulfinyl-*n*-butyl-4,4-*d*$_2$ glucosinolate tetraacetate potassium salt (S5d). Compound **S4d** (33.9 mg, 63.8 μmol), chlorosulfuric acid (127 μL, 1.9 mmol) and pyridine (1 mL) were subject to the procedure as described for **S5**, to yield **S5d** (41.7 mg, 51%). HR-ESI-MS *m/z* calcd for $C_{20}H_{25}D_5NO_{14}S_3$ $[M-K]^-$ 609.1148, found 609.1129; ^{1}H NMR (600 MHz, CD_3OD) δ_H 5.42–5.37 (2H, m), 5.06 (1H, t), 4.99 (1H, t), 4.24–4.16 (2H, m), 4.06–4.02 (1H, m), 2.78–2.74 (2H, m), 2.07 (3H, s), 2.03 (3H, s), 2.02 (3H, s), 1.98 (3H, s) and 1.95–1.86 (4H, m) ppm; ^{13}C NMR (150 MHz, CD_3OD) δ_C 172.2, 171.5, 171.2, 170.9, 158.7, 80.8, 76.7, 75.0, 71.4, 69.5, 63.3, 32.9, 26.8, 22.4, 20.7 and 20.5 (3C) ppm.

4-Methylsulfinyl-*n*-butyl glucosinolate potassium salt (4MSB). To a solution of **S5** (9.8 mg, 15.2 μmol) in anhydrous MeOH (1 ml) under nitrogen atmosphere was added 3.4 M solution of MeOK in dry MeOH (2.2 μL, 7.6 μmol). After stirring for 1 h at rt, the solution was neutralized by glacial acetic acid (0.5 μL) then it was concentrated under reduced pressure. The residue was subject to semi-preparative reversed-phase HPLC (COSMOSIL 5C$_{18}$-PAQ, 100% H_2O with 0.1% formic acid) to afford 4MSB as a white solid (4.4 mg, 61%). HR-ESI-MS *m/z* calcd for $C_{12}H_{22}NO_{10}S_3$ [M-K]$^-$ 436.0411, found 436.0403; ^{1}H NMR (600 MHz, D_2O) δ_H 5.04 (1H, d), 3.90 (1H, d), 3.70 (1H, dd), 3.59–3.54 (2H, m), 3.48–3.42 (2H, m), 2.99–2.90 (2H, m), 2.82–2.77 (2H, m), 2.70 (3H, s) and 1.96–1.78 (4H, m) ppm; ^{13}C NMR (150 MHz, D_2O) δ_C 166.5, 84.5, 82.9, 79.8, 74.6, 71.9, 63.4, 54.8, 39.1, 34.4, 28.1 and 23.9 ppm.

4-Methylsulfinyl-*n*-butyl glucosinolate-*thiohydroximate*-^{34}*S* potassium salt (4MSB-^{34}*S*). Compound **S5s** (12.1 mg, 18.7 μmol), and 3.4 M solution of MeOK in dry MeOH (2.8 μL, 9.4 μmol) were subject to the procedure as described for 4MSB, to yield 4MSB-^{34}*S* (7.3 mg, 82%). HR-ESI-MS *m/z* calcd for $C_{12}H_{22}NO_{10}S_2{}^{34}S$ [M-K]$^-$ 438.0369, found 438.0368; ^{1}H NMR (600 MHz, D_2O) δ_H 5.03 (1H, d), 3.89 (1H, d), 3.70 (1H, dd), 3.59–3.53 (2H, m), 3.48–3.43 (2H, m), 3.00–2.89 (2H, m), 2.82–2.77 (2H, m), 2.70 (3H, s) and 1.96–1.78 (4H, m) ppm; ^{13}C NMR (150 MHz, D_2O) δ_C 166.4, 84.5, 82.9, 79.8, 74.7, 71.9, 63.4, 54.8, 39.1, 34.4, 28.1 and 23.9 ppm.

4-(Methyl-*d*$_3$)sulfinyl-*n*-butyl-4,4-*d*$_2$ glucosinolate potassium salt (4MSB-*d*$_5$). Compound **S5d** (20.9 mg, 32.1 μmol), and 3.4 M solution of MeOK in dry MeOH (4.7 μL, 16.1 μmol) were subject to the procedure as described for 4MSB, to yield 4MSB-*d*$_5$ (8.7 mg, 56%). HR-ESI-MS *m/z* calcd for $C_{12}H_{17}D_5NO_{10}S_3$ [M-K]$^-$ 441.0725, found 441.0706; ^{1}H NMR (600 MHz, D_2O) δ_H 5.04 (1H, d), 3.89 (1H, d), 3.70 (1H, dd), 3.59–3.53 (2H, m), 3.49–3.42 (2H, m), 2.82–2.77 (2H, m) and 1.96–1.75 (4H, m) ppm; ^{13}C NMR (150 MHz, D_2O) δ_C 166.5, 84.5, 82.9, 79.8, 74.6, 71.9, 63.4, 34.4, 28.0 and 23.7 ppm.

Relative quantification of selected metabolites using LC–MS/MS

Sample preparation. Up to 8-mg dry weight (d wt) of the lyophilized plant materials was put into 2.0-mL tubes with 5-mm zirconia beads. A precooled extract solution [80% (v/v) methanol, 0.1% (v/v) formic acid, 8.4 nM lidocaine, and 210 nM 10-camphorsulfonic acid as internal standards] was added at 4 mg d wt mL^{-1} to extract metabolites using a Mixer Mill MM300 (Retsch) for 10 min at 20 Hz and 4 °C. After centrifugation for 2 min at 8,000 × *g* and 4 °C, 100 μL of the supernatants was transferred to new 1.5-mL tubes, dried, dissolved in 1000 μL DW, and filtered using MultiScreen HTS 384-Well Filter Plates (Merck Millipore) to prepare aqueous extract solutions at 0.4 mg d wt mL^{-1}. For more diluted solutions at 0.04 mg d wt mL^{-1} with similar concentrations of internal standards, 10 μL supernatant and 90 μL extract solution were used. To analyze the culture

media, 20 μL was mixed with 100 μL of extract solution, dried, dissolved in 1000 μL DW, and filtered to prepare an aqueous extract solution at 50-fold dilution. Extract solutions (2 μL) were subjected to LC–MS/MS analyses using a triple quadrupole mass spectrometer LCMS-8050 (Shimadzu) along with an LC/MS front-end ultra-high-performance liquid chromatography system, the Nexera MP System (Shimadzu).

Analytical conditions. Metabolites in the extract solution were separated using a Kinetex XB-C18 column (100 Å, 50 mm × 2.1 mm, 2.6 μm, Phenomenex). Solvent A consisted of water with 0.1% (v/v) formic acid, while solvent B consisted of acetonitrile with 0.1% (v/v) formic acid. The LC gradient program was set as follows: 0–0.75 min, 0.1% of solvent B; 1.2 min, 9% of solvent B; 2.4 min, 17% of solvent B; 5.7 min, 99.9% of solvent B; 5.7–6.3 min, 99.9% of solvent B; 6.33 min, 0.1% of solvent B; 6.33–9 min, 0.1% of solvent B; flow rate was 0.45 mL min^{-1}. The mass spectra were obtained by electrospray ionization under the following parameters: interface voltage, +4.00 kV (positive) or -3.00 kV (negative); nebulizing gas flow, 3.00 L min^{-1}; drying gas flow, 10.00 L min^{-1}; collision-induced dissociation gas pressure, 270 kPa; desolvation line temperature, 250 °C; heat block temperature, 400 °C. The mass accuracy in Q1 and Q3 was within 0.6 *m/z* units. Specific metabolites, including 20 GLs and two internal standards, were detected in the extract solution based on optimized selected reaction monitoring (SRM) conditions and LC retention time using different method sets dependent on experiments. Retention time and *m/z* values of precursor and product ions for each metabolite are listed in *SI Appendix*, Table S5.

Untargeted metabolomics using LC–QTOF/MS

Sample preparation. The fresh frozen samples were extracted with 5 μL mg^{-1} fresh weight (f wt) of extraction solution [80% (v/v) methanol, 2.5 μM lidocaine, and 10-camphorsulfonic acid as internal standards] using a Mixer Mill MM300 (Retsch) for 7 min at 18 Hz and 4 °C. After 10 min of centrifugation at 17,800 × *g*, the supernatant was filtered using an HLB μElution plate (Waters). Each extract (1 μL) was analyzed using LC–QTOF/MS (LC, Waters Acquity UPLC System; MS, Waters Xevo G2 Q-Tof).

Analytical conditions. LC: column, Acquity bridged ethyl hybrid (BEH) C18 (1.7 μm, 2.1 mm × 100 mm, Waters); solvent system, solvent A (water including 0.1% formic acid) and solvent B (acetonitrile including 0.1% formic acid); gradient program, 99.5%A/0.5%B at 0 min, 99.5%A/0.5%B at 0.1 min, 20%A/80%B at 10 min, 0.5%A/99.5%B at 10.1 min, 0.5%A/99.5%B at 12.0 min, 99.5%A/0.5%B at 12.1 min, and 99.5%A/0.5%B at 15.0 min; flow rate, 0.3 mL/min at 0 min, 0.3 mL/min at 10 min, 0.4 mL/min at 10.1 min, 0.4 mL/min at 14.4 min, and 0.3 mL/min at 14.5 min; column temperature, 40 °C; MS detection: polarity, positive/negative; capillary voltage, +3.00 kV (positive)/-2.75 kV (negative); cone voltage, 25.0 V; source temperature, 120 °C; desolvation

temperature, 450 °C; cone gas flow, 50 L/h; desolvation gas flow, 800 L/h; collision energy, 6 V; mass range, *m/z* 50–1500; scan duration, 0.1 sec; interscan delay, 0.014 sec; data acquisition, centroid mode; Lockspray (Leucine enkephalin); scan duration, 1.0 sec; interscan delay, 0.1 sec. MS/MS data were acquired in the ramp mode as per the following analytical conditions: (1) MS: polarity, positive/negative; mass range, *m/z* 50–1500; scan duration, 0.1 sec; interscan delay, 0.014 sec; data acquisition, centroid mode and (2) MS/MS: polarity, positive/negative; mass range, *m/z* 50–1500; scan duration, 0.02 sec; inter-scan delay, 0.014 sec; data acquisition, centroid mode; collision energy, ramped from 10 to 50 V. In this mode, MS/MS spectra of the top 10 ions (> 1000 counts) in an MS scan were automatically obtained. If the ion intensity was less than 1000, MS/MS data acquisition was not performed and moved to the next top 10 ions. Data acquisition was performed using MassLynx 4.1 (Waters).

Structural analysis of the potential intermediates detected by untargeted metabolomics.

General. Molecular formulas of the candidate metabolites were predicted using ChemCalc (https://www.chemcalc.org/) (3) based on MS and MS/MS ions. The record IDs (P/N_xxxx) correspond to those in *SI Appendix*, Tables S2, S3 and Dataset S1. Key MS/MS fragments described below are displayed in *SI Appendix*, Fig. S4 with chemical structures.

4-Methylsulfinyl-*n*-butylamine (4MSB-NH$_2$). Out of 26 record pairs listed in *SI Appendix*, Table S2, pair 4 containing P_0203 (*m/z* 136.0797) and P_0212 (*m/z* 141.1104) was considered to have a relatively small structure with a number of MS/MS fragments. The most plausible molecular formula of P_0203 was calculated to be $C_5H_{13}NOS$, which is consistent with that of a sulforaphane (SFN) derivative in which the isothiocyanate group is replaced by an amine (NH_2) group. The -17 Da fragment ion detected in MS/MS data (*m/z* 119.0515 calcd. for $C_5H_{11}OS^+$, 119.0525) also supported the presence of an NH_2 group, while the chemical formula of another characteristic MS/MS ion (*m/z* 72.0803) was calculated to be $C_4H_{10}N^+$ (72.0808), consistent with that of pyrrolidine. Generation of pyrrolidine during MS/MS fragmentation can be explained by an attack of the NH_2 group to a carbon bound to a methylsulfinyl (CH_3S=O) group followed by release of the CH_3S=O group, indicating that an NH_2 group and a CH_3S=O group are bound to each terminal of a linear butyl chain. Overall, the metabolite corresponding to pair 4 was inferred to be 4-methylsulfinyl-*n*-butylamine.

Raphanusamic acid (RA). Records P_0347 (*m/z* 163.9841) and N_0104 (*m/z* 161.9683) showed the highest correlation coefficient between samples treated with 4MSB and with 4MSB-d_5 among all records in each ion mode (r_S=0.958 and 0.965, respectively). The KNApSAcK database annotated these records to 2-thioxo-1,3-thiazolidine-4-carboxylic acid, also referred to as RA. Theoretical *m/z* values of RA were comparable with the detected *m/z* values. A characteristic

MS/MS fragment of P_0347 (*m/z* 117.9781 calcd. for $C_3H_4NS_2^+$, 117.9780) further supported that the KNApSAcK annotation is correct.

Conjugate of sulforaphane with glutathione (SFN-GSH). Based on the identifications of 4MSB-NH_2 and RA, we hypothesized that 4MSB was processed dependent on GSH as described in the main text, like the PEN2 immune pathway. P_1148 (*m/z* 485.1184) and P_1139 (*m/z* 490.1494) in record pair 23 were the most intense signals in positive ion mode and their *m/z* values were comparable with the theoretical *m/z* values of the SFN-GSH conjugate ($C_{16}H_{29}N_4O_7S_3^+$, 485.1193) and its D_5-labeled form ($C_{16}H_{24}D_5N_4O_7S_3^+$, 490.1507), respectively. These records had several common MS/MS fragment ions, one of which (*m/z* 308.0915 calcd. for $C_{10}H_{18}N_3O_6S^+$, 308.0911) supported the presence of GSH in the structure. On the other hand, some MS/MS fragment ions in P_1148 were labeled with five deuteriums in P_1139, such as the pair of *m/z* 136.0795 and 141.1108 corresponding to a 4MSB-amino group. Considering other common and labeled MS/MS fragments shown in *SI Appendix*, Fig. S4, the metabolite corresponding to pair 23 was inferred to be SFN-GSH.

Conjugate of sulforaphane with gamma-glutamylcysteine (SFN-γEC). P_1042 (*m/z* 428.0979) and P_1057 (*m/z* 433.1271) in record pair 20 had the second highest intensities in positive ion mode, while the mass difference between P_1042 and P_1148 (SFN-GSH) suggested a replacement of Gly residue to a hydroxyl group (57.0205 calcd. for C_2H_3NO, 57.0215). P_1042 and P_1148 had almost the same retention time, while the accumulation patterns were apparently different (Fig. 3*B*), indicating that P_1042 was not a fragment ion of SFN-GSH. When MS/MS fragment ions between P_1042 and P_1057 were compared, the presence of γEC moiety instead of GSH as a common fragment was suggested, consistent with the mass difference from SFN-GSH. In contrast, the 4MSB-amino group was also observed as unlabeled and labeled forms. Considering other common and labeled MS/MS fragments shown in *SI Appendix*, Fig. S4, the metabolite corresponding to pair 20 was inferred to be SFN-γEC.

Conjugate of sulforaphane with cysteine (SFN-Cys). Based on the findings of metabolites described above, we focused on P_0782 (*m/z* 299.0555) and P_0796 (*m/z* 304.0873) in record pair 13, whose *m/z* values were comparable with the theoretical *m/z* values of the SFN-Cys conjugate ($C_9H_{19}N_2O_3S_3^+$, 299.0552) and its D_5-labeled form ($C_9H_{14}D_5N_2O_3S_3^+$, 304.0866), respectively. Although the 4MSB-amino group as unlabeled and labeled forms was included, the MS/MS data was not sufficient to elucidate which -SH or -NH_2 in Cys was used for conjugation. When SFN was incubated with Cys for 1 h at basic pH condition, as described below, the respective metabolite found in the tissue extract was observed at similar retention time and the MS/MS spectrum was also comparable (*SI Appendix*, Fig. S5). Considering that the ITC-Cys conjugate at this condition

is stable as the dithiocarbamate form, the conjugation of SFN with Cys during 4MSB processing was elucidated to be performed using the thiol group in Cys.

Analysis of the authentic standards

To confirm the potential intermediates inferred in the metabolomics analyses, retention times and MS/MS spectra of authentic standards were compared in LC–MS/MS with those observed in the plant extracts. 4MSB-NH_2 and RA were purchased from Toronto Research Chemicals and Sigma-Aldrich, respectively, and dissolved in water at 50 μM. For SFN-GSH, SFN-γEC, and SFN-Cys, 25 μL of (D,L)-sulforaphane dissolved in acetonitrile at 560 μM was added to 225 μL aqueous $NaHCO_3$ solutions (0.1 M) containing GSH, γEC, or Cys at 2 mM. After a short vortex, reaction mixtures were incubated at room temperature for 1 h. The plant extracts (0.04 mg d wt mL^{-1}) were prepared from eight-day-old seedlings treated with 100 μM 4MSB for 24 h as described in the main text. Each solution (2 μL) was analyzed using LC–MS/MS.

5-Oxoprolinase (OPase) assay

Approximately 200 Col-0 and *oxp1-1* seeds were cultured in 90 × ϕ20 mm petri dishes with 25 mL S1500 or S15 media for 14 days. Ten to fifteen seedlings were washed with 3 mL of sterile DW three times, frozen in 2.0-mL tubes containing 5-mm zirconia beads, and stored at -80 °C until use. The fresh frozen seedlings (approximately 130 mg f wt) were homogenized using a Mixer Mill MM300 (Retsch) for 3 min at 20 Hz and 4 °C and suspended in 3 mL g^{-1} f wt of an extraction buffer [20 mM HEPES (pH 7.4) and 5 mM DTT] on ice. After centrifugation at 20,380 × *g* at 4 °C for 5 min, the supernatants were placed in Amicon Ultra-0.5 30 kDa Centrifugal Filter Units (Merck Millipore). Centrifugation at 20,380 × *g* at 4 °C for 20 min followed by dilution of the concentrated solutions to approximately 450 μL using the extraction buffer was repeated three times to remove endogenous small molecules, including Glu and Cys. Protein concentrations were determined using the Bradford assay. To the 0.2 mL tubes were added 30 μL of assay buffer premix, 18 μL of the protein solution, and 2 μL of 5-oxoproline or procysteine (50 mM) in this order. The reaction mixture finally contained 100 mM Na glycinate (pH 9.5), 5 mM ATP, 2.5 mM $MnCl_2$, 2.5 mM $MgCl_2$, 20 mM $(NH_4)_2SO_4$, 5 mM DTT, and 2 mM substrate. The control was prepared using DW instead of substrate to correct for internal Glu and Cys levels. A series of standard solutions [30 μL of assay buffer premix, 18 μL of extraction buffer, and 2 μL of Glu solution (300, 100, 30, 10, 3, and 0 μM) or Cys solution (10, 3, 1, 0.3, 0.1, and 0 μM)] was prepared to generate a calibration curve. Reaction mixtures were incubated at 30 °C for 120 min and the reaction was quenched by adding 5 μL of 1 M acetic acid followed by heating at 99 °C for 5 min. The quenched mixtures were centrifuged at 20,380 × *g* at 4 °C for 5 min and then 2 μL of the 10-fold diluted supernatant was subject to LC–MS/MS to analyze the Glu and Cys signals. Glu and Cys concentrations in the reaction mixtures were calculated based on peak areas using linear standard curves (*SI Appendix,* Fig. S18*B*). Glu

or Cys concentrations with substrate were subtracted by those without substrate to obtain the enzymatic capacity of tissue samples to hydrolyze 5-OP and pCys, respectively, as nmol min^{-1} mg^{-1} protein. Five biological replicates were tested for each experimental group.

Transient gene expression in *N. benthamiana*

Nicotiana benthamiana plants were grown at 25 °C under a 16-h light/8-h dark cycle in an environmentally controlled incubation room. For transient gene expression in *N. benthamiana*, full-length coding sequences of *AtBGLU28* (*AT2G44460.1*), *AtBGLU30* (*AT3G60140.1*), and *AtTGG1* (*AT5G26000.1*) were amplified from Col-0 cDNA by PCR and assembled using an In-Fusion® HD cloning kit (Clontech) into a binary vector pEAQ-HT (with 35S promoter and *C*-terminal His_6 tag) (4). With GFP as the negative control, the same vector with an *N*-terminal His_6 tag was used. Primers used for cloning are listed in the *SI Appendix*, Table S4. *Agrobacterium tumefaciens* strain GV3101 was used to deliver respective transgenes in *N. benthamiana* leaves as previously reported (5). Briefly, *A. tumefaciens* cells collected from overnight culture were resuspended at an OD_{600} of 0.3 in an infiltration buffer [10 mM MES-NaOH (pH 5.6), 10 mM $MgCl_2$, and 150 μM acetosyringone] and then infiltrated into the third to fifth true leaves of four- to five-week-old *N. benthamiana* plants using a needleless syringe. After two days, inoculated leaves were frozen in liquid nitrogen after measuring fresh weight and stored at -80 °C until use. Protein expression in the transfected leaves was monitored using the standard western blot technique. Briefly, the protein extracts prepared for the myrosinase assay were loaded onto a precast gel (SuperSep™ Ace 12.5%, FUJIFILM Wako Chemical). The loading volume was adjusted to 5 μg total protein based on the Bradford assay result. His_6-tagged proteins were detected using a mouse anti-His-tag monoclonal antibody (D291-3S, MBL Life Science) and a rabbit anti-mouse IgG H&L (alkaline phosphatase) (ab6729, Abcam) in 1: 1,000 and 1:10,000 dilutions, respectively, followed by staining with 1-Step™ NBT/BCIP Substrate Solution (Thermo). Total proteins were stained with Coomassie Brilliant Blue.

Myrosinase assay

Frozen leaf material of *N. benthamiana* (1,000–1,600 mg f wt) was ground in a precooled mortar and suspended in 3 mL g^{-1} f wt of extraction buffer [50 mM PIPES (pH 7.0), 150 mM NaCl, and cOmplete Protease Inhibitor Cocktail (Roche)] on ice. After centrifugation at 20,380 × *g* at 4 °C for 10 min, supernatants were placed into Amicon Ultra-4 30 kDa Centrifugal Filter Units (Merck Millipore) using a syringe equipped with a 0.45-μm membrane filter. Small aliquots of the supernatants were collected and subjected to western blotting, as described above. Centrifugation at 20,380 × *g* at 4 °C for 20 min followed by dilution of the concentrated solution to 3.5 mL using the extraction buffer was repeated three times to remove endogenous small molecules, including glucose. Protein concentrations were determined using the Bradford assay. To a 96-well microtiter

plate was added 30 µL 5 × enzyme mix of LabAssay™ Glucose Kit (FUJIFILM Wako Chemicals), 115 µL protein solution, and 5 µL GL substrate (5 mM) in that order. The final reaction mixture contained 1 × enzyme mix [mutarotase (0.13 U mL^{-1}), glucose oxidase (9.0 U mL^{-1}), peroxidase (0.65 U mL^{-1}), 4-aminoantipyrine (0.50 mmol mL^{-1}), ascorbate oxidase (2.7 U mL^{-1}), and phenol (1.1 mmol mL^{-1})] and 25 nmol of a GL substrate. The control was prepared using DW instead of GL solution to correct for internal glucose levels. A series of standard glucose solutions [30 µL 5× enzyme mix, 115 µL extraction buffer, and 5 µL glucose solution (5, 3, 2, 1, 0.5, and 0 mM)] was prepared for use in the generation of a calibration curve. All the samples were duplicated in the same plate. Glucose release was determined by measuring the absorbance of a colored product, *N*-(4-antipyryl)-*p*-benzoquinone imine at 490 nm on an iMark™ Microplate Absorbance Reader (Bio-Rad). Data were recorded every minute for 90 min at room temperature. Glucose concentrations at each time were calculated based on a linear standard curve with means of the background controls subtracted from the means of the duplicate measurements. The time ranges over which glucose concentrations increased linearly (between 10 and 60 min) was used to determine myrosinase activity. The enzymatic capacity of the tissue samples to hydrolyze GLs was expressed as nmol min^{-1} mg^{-1} protein. Five (*At*BGLU28 and *At*TGG1) or three (GFP) biological replicates were tested under each condition.

Hydroponic culture experiment

Arabidopsis thaliana plants were grown at 22 °C in an environmentally controlled incubation room under a 16-h light/8-h dark cycle. Seeds of Col-0 and *bglu28 bglu30* were germinated on rockwool cylinders moistened with DW. The rockwool cylinders with seven-day-old seedlings were then transferred to cylinder holders on the lids of plastic containers, which were filled with culture media under the indicated conditions. After three weeks, the fresh weights of the whole aerial parts were measured and then rosette leaves were frozen in 25 mL tubes and stored at -80 °C until use. The plants that had apparently withered were removed from further analyses. Lyophilized plant materials were subject to the metabolite analyses. Twelve individual plants were analyzed in each experimental group. The experiment was repeated three times.

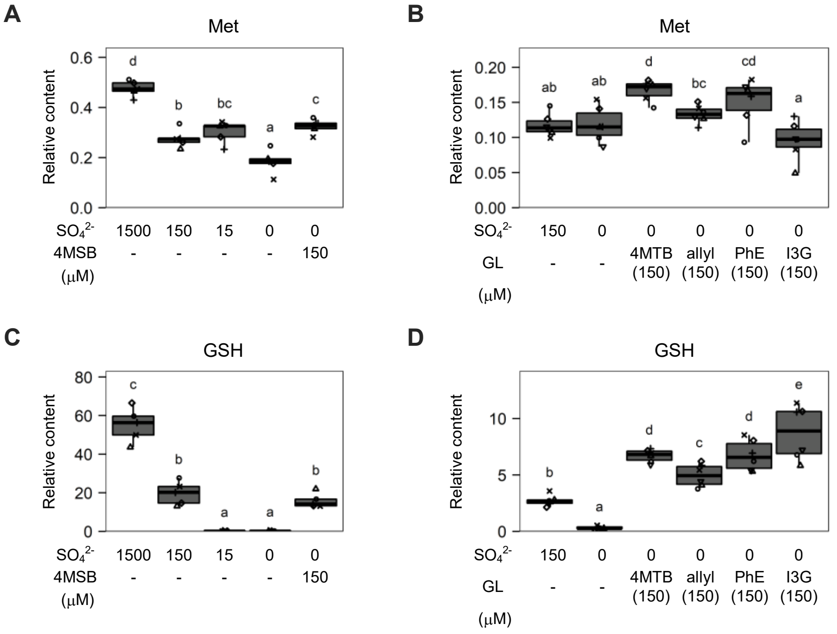

Fig. S1. Primary sulfur metabolites in *Arabidopsis thaliana* seedlings under sulfur deficiency. Relative contents of methionine (*A* and *B*) and glutathione (*C* and *D*) in plant tissues of 14-day-old Col-0 seedlings. The seedlings were cultured at different sulfur concentrations with or without 4-methylsulfinyl-*n*-butyl glucosinolate (4MSB) (*A* and *C*), or with different GL species supplied at 150 μM under no sulfate condition (*B* and *D*). N=5 in (*A* and *C*) and N=6 in (*B* and *D*). Point shapes indicate individual experimental batches. Letters indicate statistical significance corresponding to two-tailed *t*-tests based on a linear mixed model with batches as a random factor, followed by a correction for multiple comparisons controlling false discovery rate (FDR) ($P < 0.05$). Abbreviations of GLs correspond to those listed in Table 1.

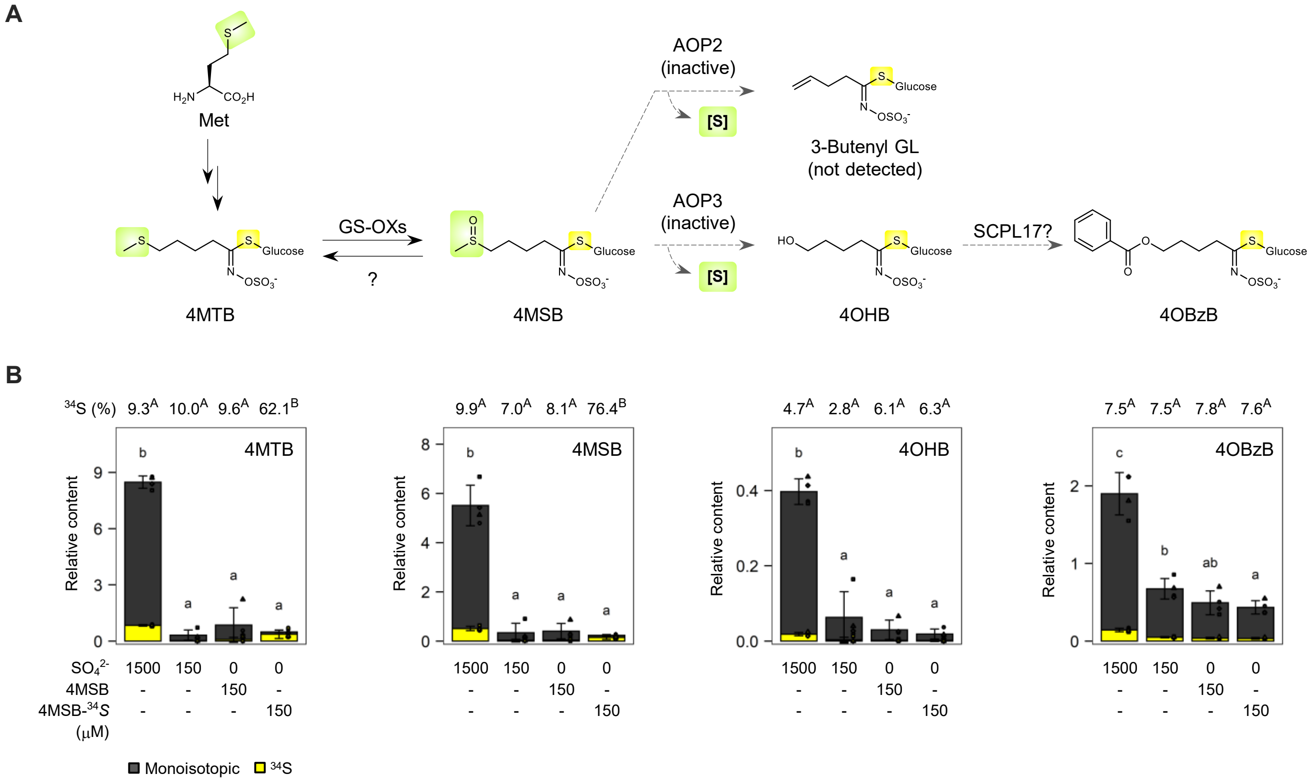

Fig. S2. Incorporation of ^{34}S from 4MSB into other glucosinolate (GL) species. (*A*) Biosynthetic pathway of 4MSB and related analogues in *A. thaliana*. Sulfur atoms derived from Met and GSH are highlighted in green and yellow, respectively. Abbreviations of GLs correspond to those listed in Table 1. 4MTB is known to be oxidized by flavin monooxygenases (GS-OXs) to produce 4MSB (6, 7), while there could be a reductive pathway from 4MSB to 4MTB. 4MSB is also suggested to be further modified by a specific monooxygenase, AOP3, to generate 4OHB (8, 9), followed by benzoylation potentially catalyzed by serine carboxypeptidase-like 17 (SCPL17) to produce 4OBzB (10). AOP2 generates GLs with unsaturated hydrocarbon side chains (8), although AOP2- and AOP3-related modifications are considered inactive in Col-0 seedlings. (*B*) Relative contents of 4MTB, 4MSB, 4OHB, and 4OBzB in 14-day-old Col-0 seedlings cultured with SO_4^{2-}, 4MSB, or 4MSB-^{34}S in the respective nutrient solution. Contents of monoisotopic compound and the ^{34}S isotope are shown in dark-gray and yellow, respectively, as a stacked bar chart. Abundance of the ^{34}S isotope relative to the total content is shown above as numbers. 3-Butenyl GL was undetectable in our LC–MS/MS system. Point shapes indicate individual experimental batches. Letters indicate statistical significance in total metabolite content (lowercase) and abundance of the ^{34}S isotope (uppercase), which correspond to two-tailed *t*-tests based on a linear mixed model with batches as a random factor, followed by a correction for multiple comparisons controlling FDR ($P < 0.05$). Error bars indicate SD.

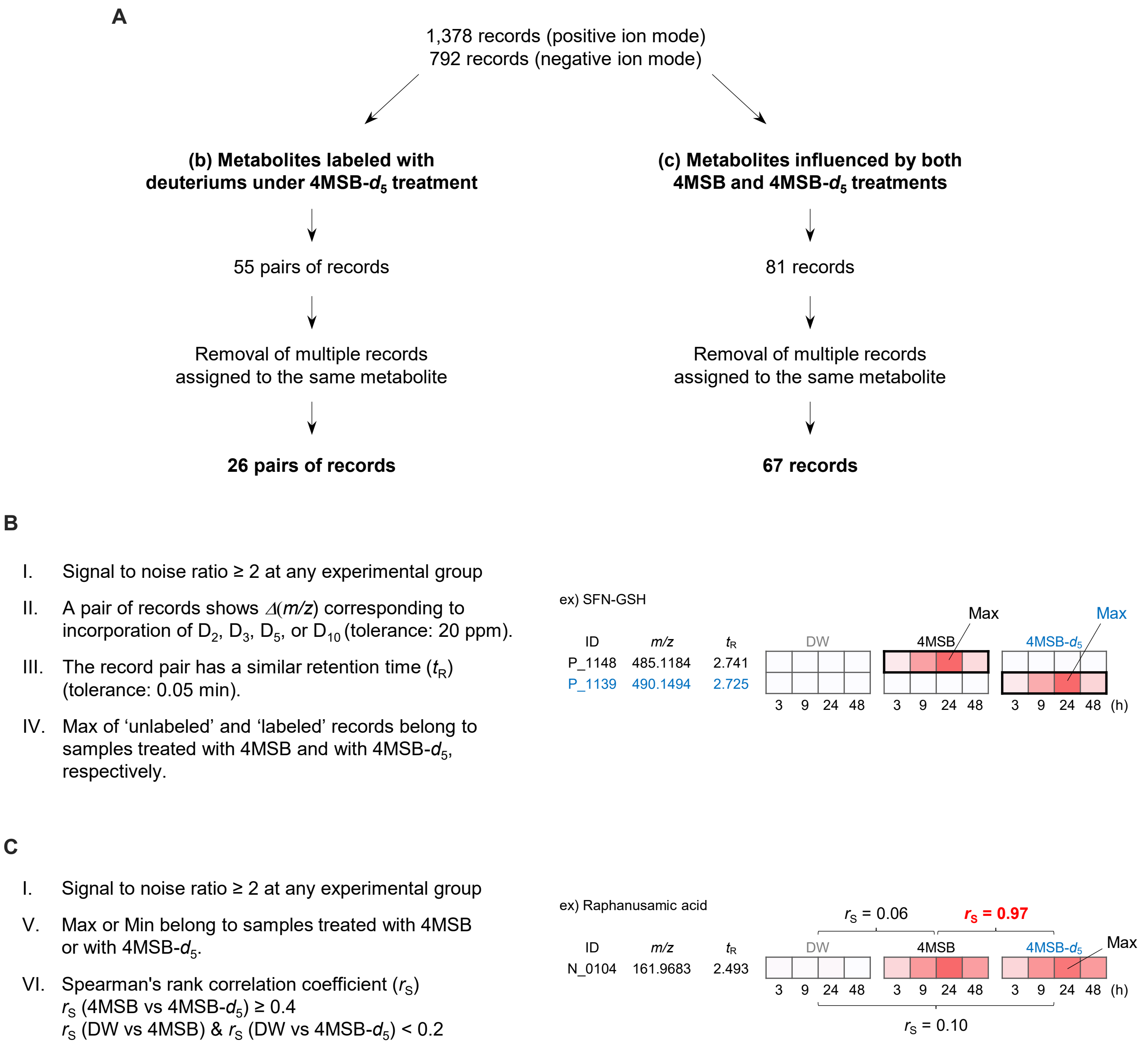

Fig. S3. Workflow to narrow down the untargeted metabolomics data. (*A*) Overview of the workflow. After each screening, records potentially assigned to the same metabolite (e.g. $[M+H]^+$, $[M-H]^-$, or $[2M+H]^+$ ions) were organized to a record showing the highest signal intensity. (*B*) Criteria of the screening for metabolites labeled with deuteriums under 4MSB-d_5 treatment. For example, records P_1148 and P_1139 inferred to be SFN-GSH show a mass difference corresponding to incorporation of five deuteriums (Δ = 5.0310 Da) at a similar retention time (Δ = 0.016 min). Mean signal intensities in each experimental group are displayed as two-color gradient (low in white; high in red), which show the maximum at 24 h after the respective 4MSB treatments. (*C*) Criteria of the screening for metabolites influenced under both 4MSB and 4MSB-d_5 treatments in a similar manner. For example, a record N_0104 inferred to be raphanusamic acid shows a similar accumulation pattern under both 4MSB treatments, resulting in a high Spearman's rank correlation coefficient (r_S = 0.97) between those samples. Records obtained from each screening are available in *SI Appendix*, Tables S2 and S3.

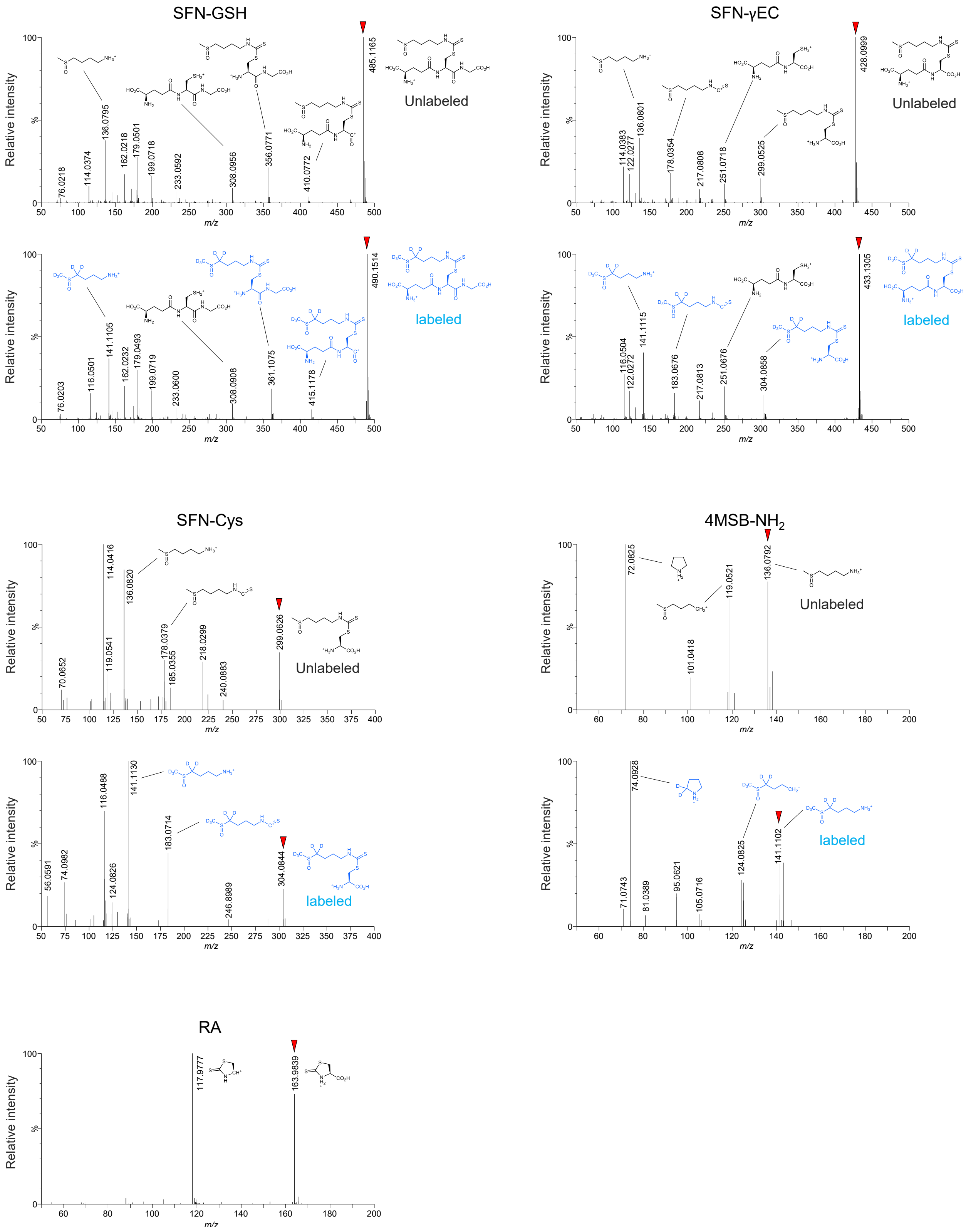

Fig. S4. Tandem mass spectrometry (MS/MS) data of the potential intermediates. High-resolution MS/MS data were recorded on LC–QTOF/MS. Red arrows indicate precursor ions. Chemical structures of precursor and characteristic fragment ions are displayed on the spectra. Deuterated precursor/fragment ions are colored in blue.

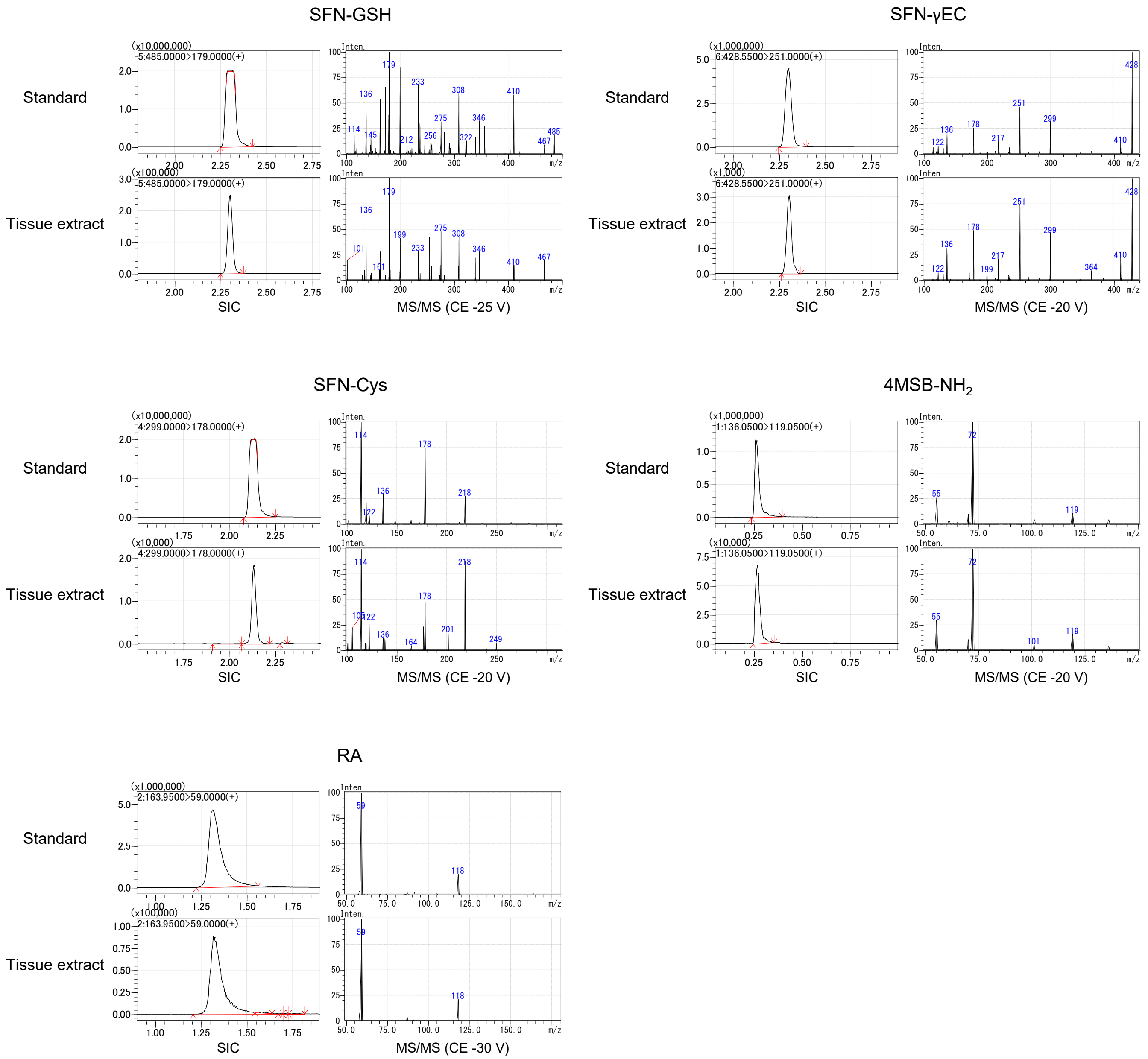

Fig. S5. Identification of the potential intermediates using authentic standards. Selected ion chromatograms (SIC) and MS/MS spectra were recorded using LC–MS/MS. Peaks detected in the tissue extract of eight-day-old Col-0 seedlings treated with 100 μM of 4MSB for 24 h were compared with those of authentic standards. CE, collision energy.

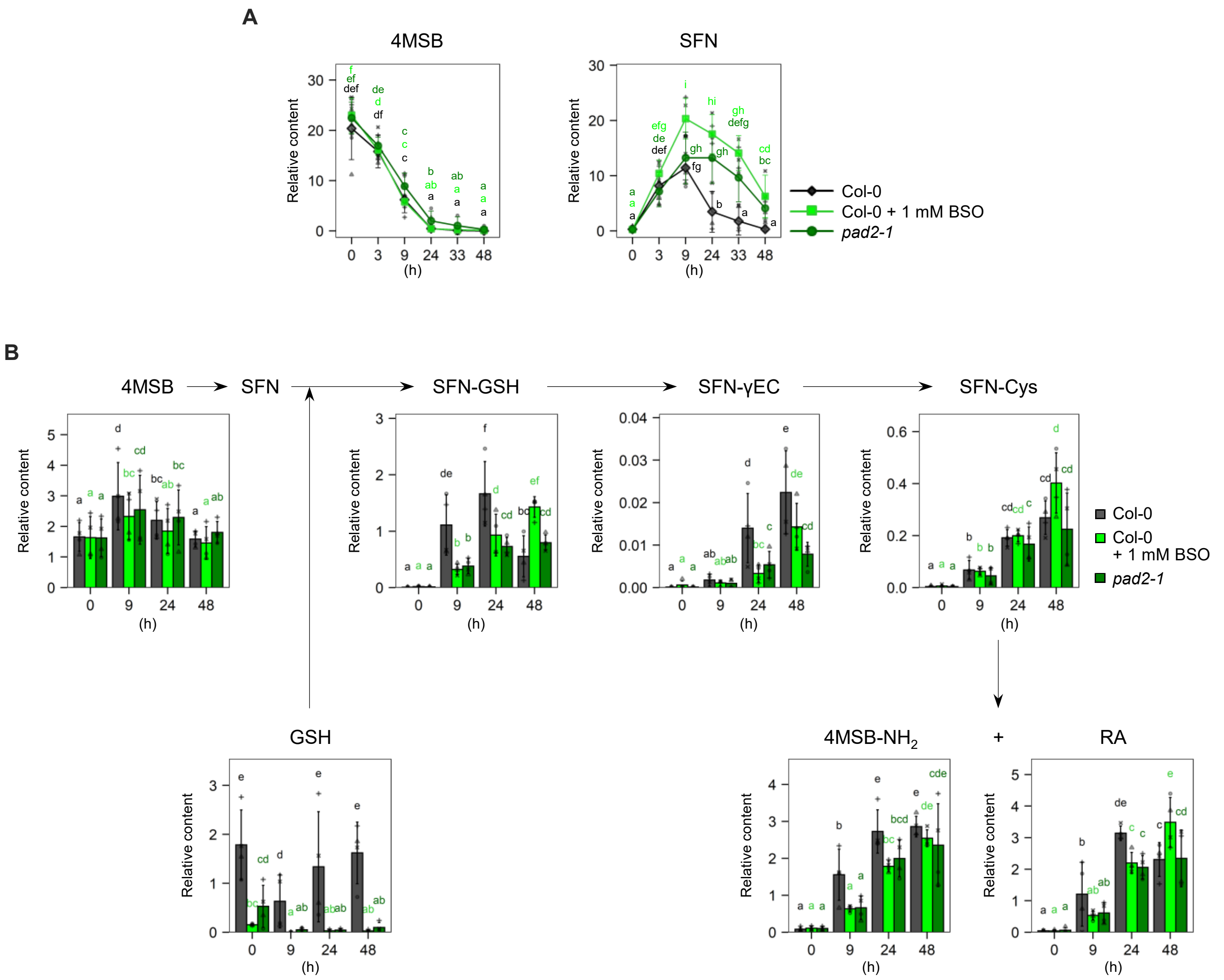

Fig. S6. Effect of inhibited glutathione biosynthesis on the glucosinolate processing. Eight-day-old seedlings of Col-0 (gray), Col-0 pre-treated for 24 h with 1 mM of buthionine sulfoximine (BSO), an inhibitor of GSH biosynthesis (light green), and the GSH-deficient *pad2-1* mutant (dark green) were treated with 100 μM of 4MSB for 48 h. Relative contents of (*A*) 4MSB and SFN in the culture media as well as (*B*) 4MSB, GSH and the intermediates in plant tissues were monitored (N=4). Point shapes indicate individual experimental batches. Letters indicate statistical significance corresponding to two-tailed t-tests based on a linear mixed model with batches as a random factor, followed by a correction for multiple comparisons controlling FDR (P < 0.05). Error bars indicate SD.

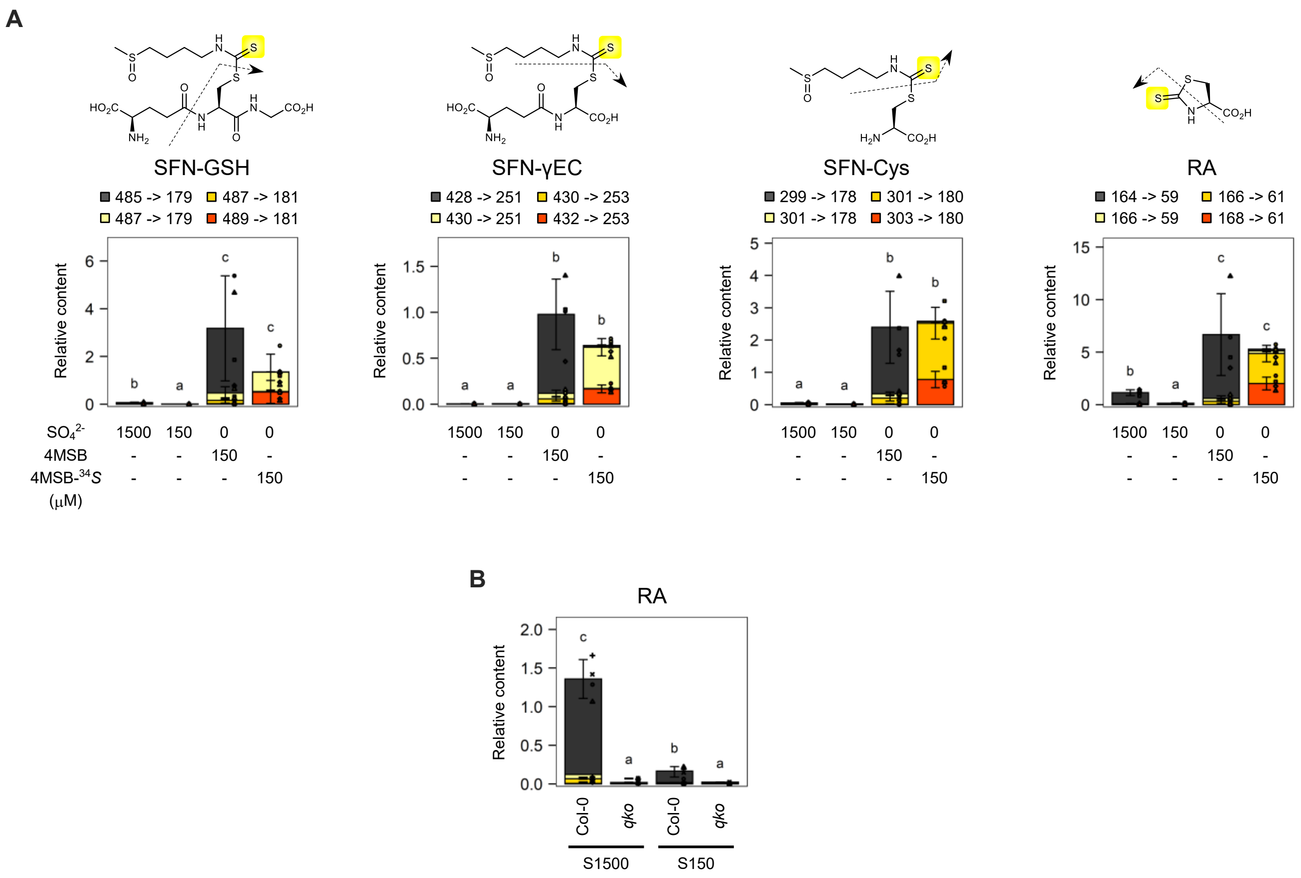

Fig. S7. Sulfur reallocation through the glutathione-dependent glucosinolate turnover. (*A*) Relative contents of the potential intermediates in 14-day-old Col-0 seedlings cultured with SO_4^{2-}, 4MSB, or 4MSB-^{34}S in the respective nutrient solution (*N*=4). The sulfur atom potentially derived from the thioglucosidic bond in 4MSB is highlighted in yellow. Dashed arrows indicate MS/MS fragment ions used for the calculation of each metabolite. Contents of monoisotopic (dark gray), +2 Da (light/dark yellow), and +4 Da (orange) compounds are shown as a stacked bar chart. (*B*) Relative content of isotopic RA in Col-0 and the *cyp79b2 cyp79b3 myb28 myb29* (*qko*) mutant at S1500 and S150 conditions (*N*=4). Point shapes indicate individual experimental batches. Letters indicate statistical significance in total metabolite content corresponding to two-tailed *t*-tests based on a linear mixed model with batches as a random factor, followed by a correction for multiple comparisons controlling FDR ($P < 0.05$). Error bars indicate SD.

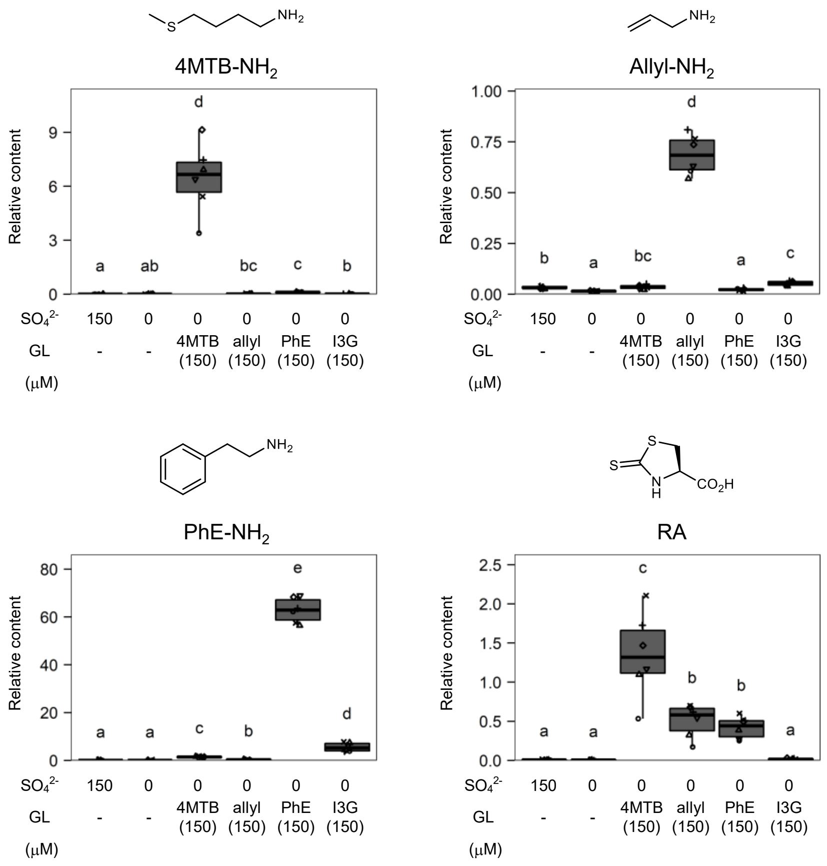

Fig. S8. Production of amines and raphanusamic acid under glucosinolate treatment. Relative contents of RA and amine compounds whose side chains correspond to those of supplied GLs in 14-day-old Col-0 seedlings (*N*=6). Point shapes indicate individual experimental batches. Letters indicate statistical significance corresponding to two-tailed *t*-tests based on a linear mixed model with batches as a random factor, followed by a correction for multiple comparisons controlling FDR ($P < 0.05$). Abbreviations of GLs correspond to those listed in Table 1.

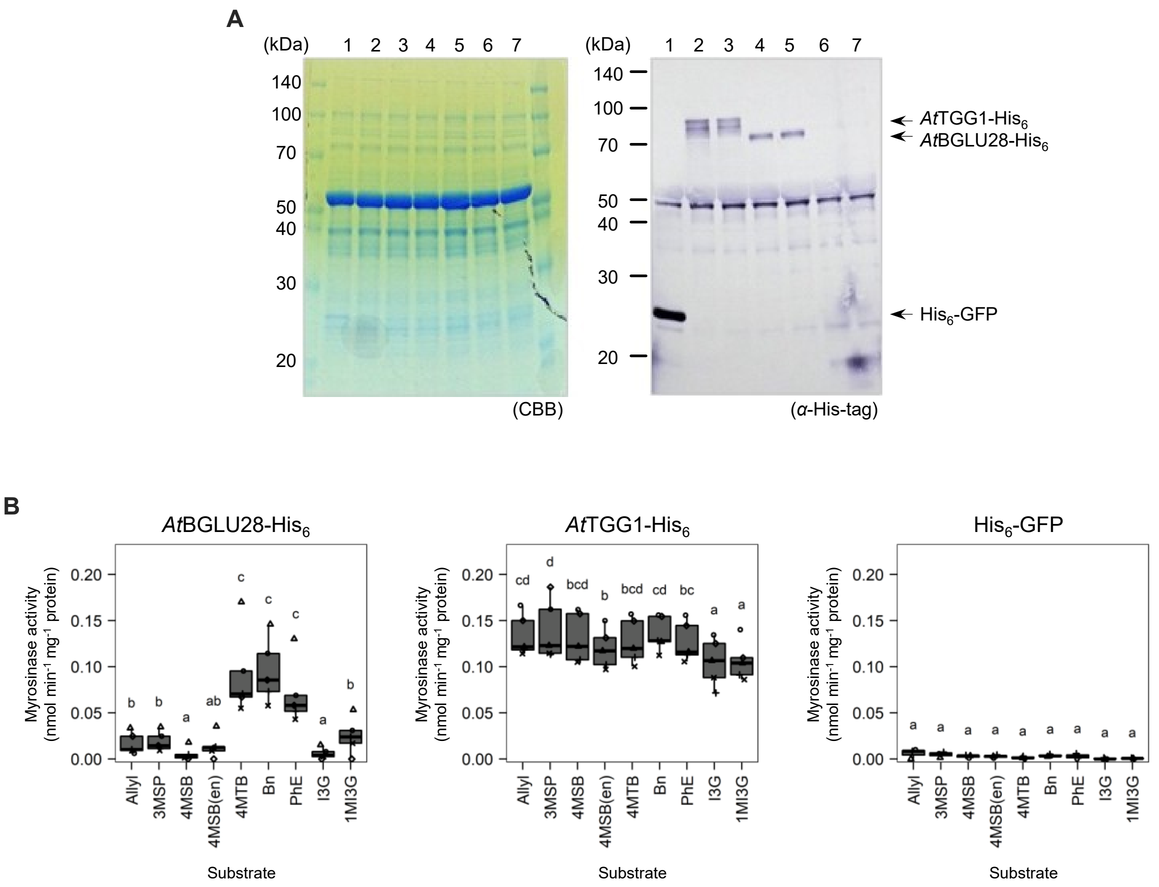

Fig. S9. Myrosinase activities of *At*BGLU28 and *At*TGG1 transiently expressed in *Nicotiana benthamiana*. (*A*) Coomassie Brilliant Blue (CBB) staining (left) and western blotting (right, anti-His-tag antibody) of the protein extracts from *N. benthamiana* leaves after 2 days of inoculation by *Agrobacterium tumefaciens* possessing a pEAQ-HT expression vector. Crude protein (5 µg) was loaded onto each lane. Lane 1, His$_6$-GFP (28.1 kDa); Lanes 2/3, *At*TGG1-His$_6$ (62.0 kDa); Lanes 4/5, *At*BGLU28-His$_6$ (68.2 kDa); Lanes 6/7, *At*BGLU30-His$_6$ (67.8 kDa). Up-shift of the TGG1 and BGLU28 bands than predicted is probably because of glycosylation (11). His$_6$-GFP showed the highest expression based on the visible band on the CBB-stained gel, while BGLU30 was not expressed in this condition. (*B*) Myrosinase activity in crude protein extracts from *N. benthamiana* leaves expressing *At*BGLU28-His$_6$, *At*TGG1-His$_6$, or His$_6$-GFP, based on the production of D-glucose in the presence of indicated GL species as a substrate (N=5). Point shapes indicate individual experimental batches. Letters indicate statistical significance corresponding to two-tailed t-tests based on a linear mixed model with batches as a random factor, followed by a correction for multiple comparisons controlling FDR ($P < 0.05$). Although the absolute rates of myrosinase activities cannot be compared between BGLU28 and TGG1 because of the different expression levels, while the activity of the extract expressing GFP was at a background level despite the highest expression based on (*A*).

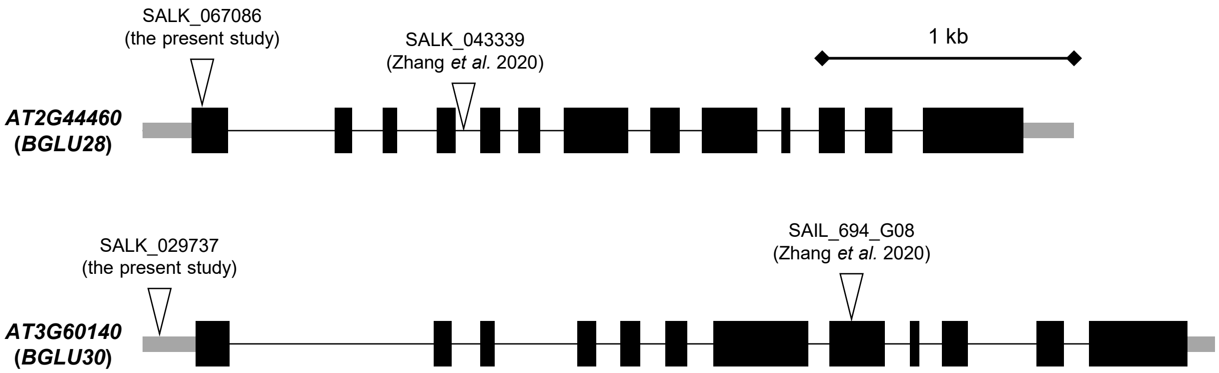

Fig. S10. Gene structures of *BGLU28* and *BGLU30*. Black boxes show exons while untranslated regions are colored in gray. White arrows indicate positions of T-DNA insertion in the knock-out mutants used in the present study and in the previous report (12).

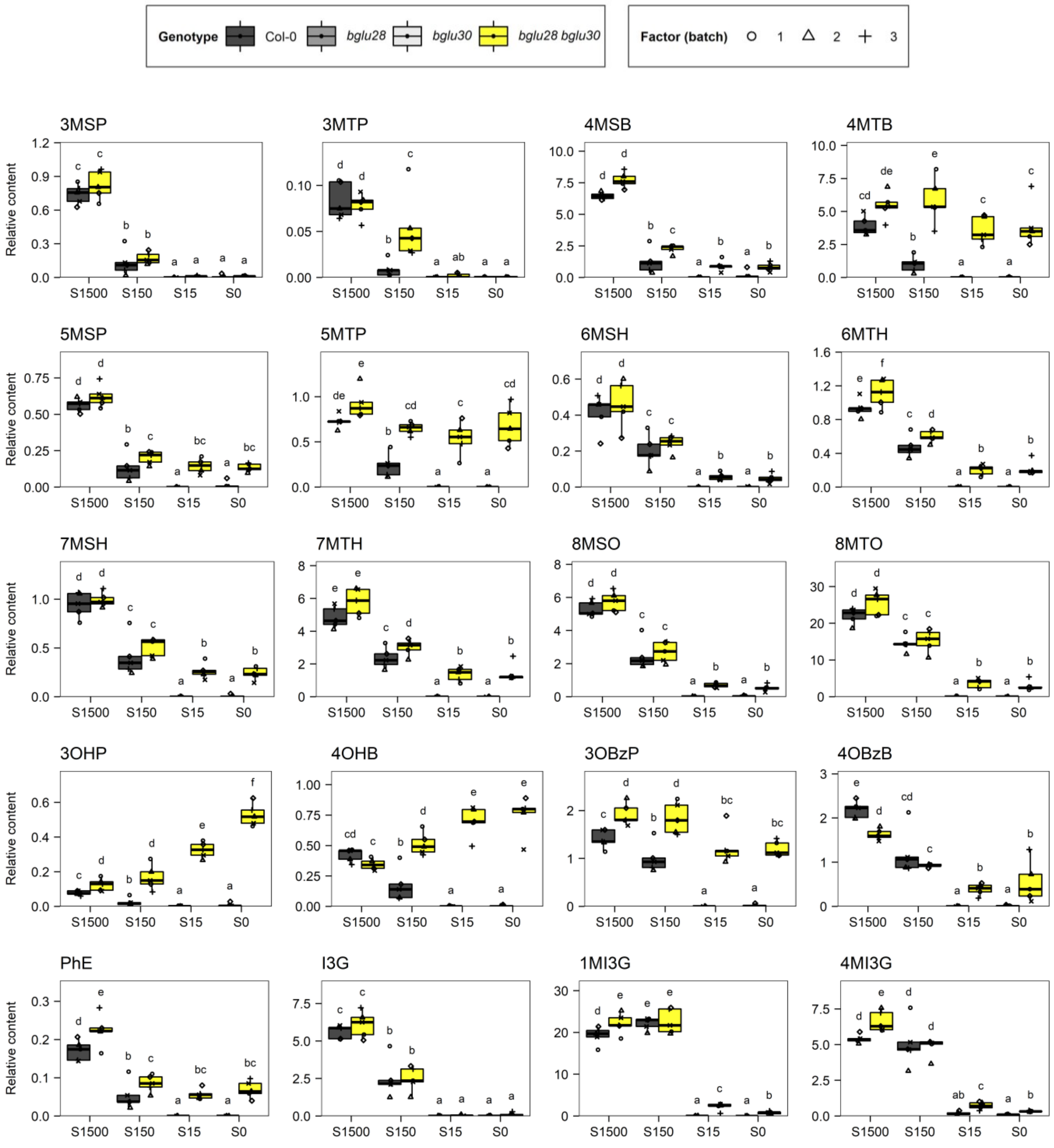

Fig. S11. Glucosinolate degradation in the *bglu28 bglu30* mutant under constitutive sulfur deficiency. Relative GL contents in plant tissues of 14-day-old seedlings (N=5). S1500, S150, S15 and S0 indicate 1500, 150, 15, and 0 μM SO_4^{2-}, respectively. Point shapes indicate individual experimental batches. Letters indicate statistical significance corresponding to two-tailed t-tests based on a linear mixed model with batches as a random factor, followed by a correction for multiple comparisons controlling FDR ($P < 0.05$). Abbreviations of GLs correspond to those listed in Table 1.

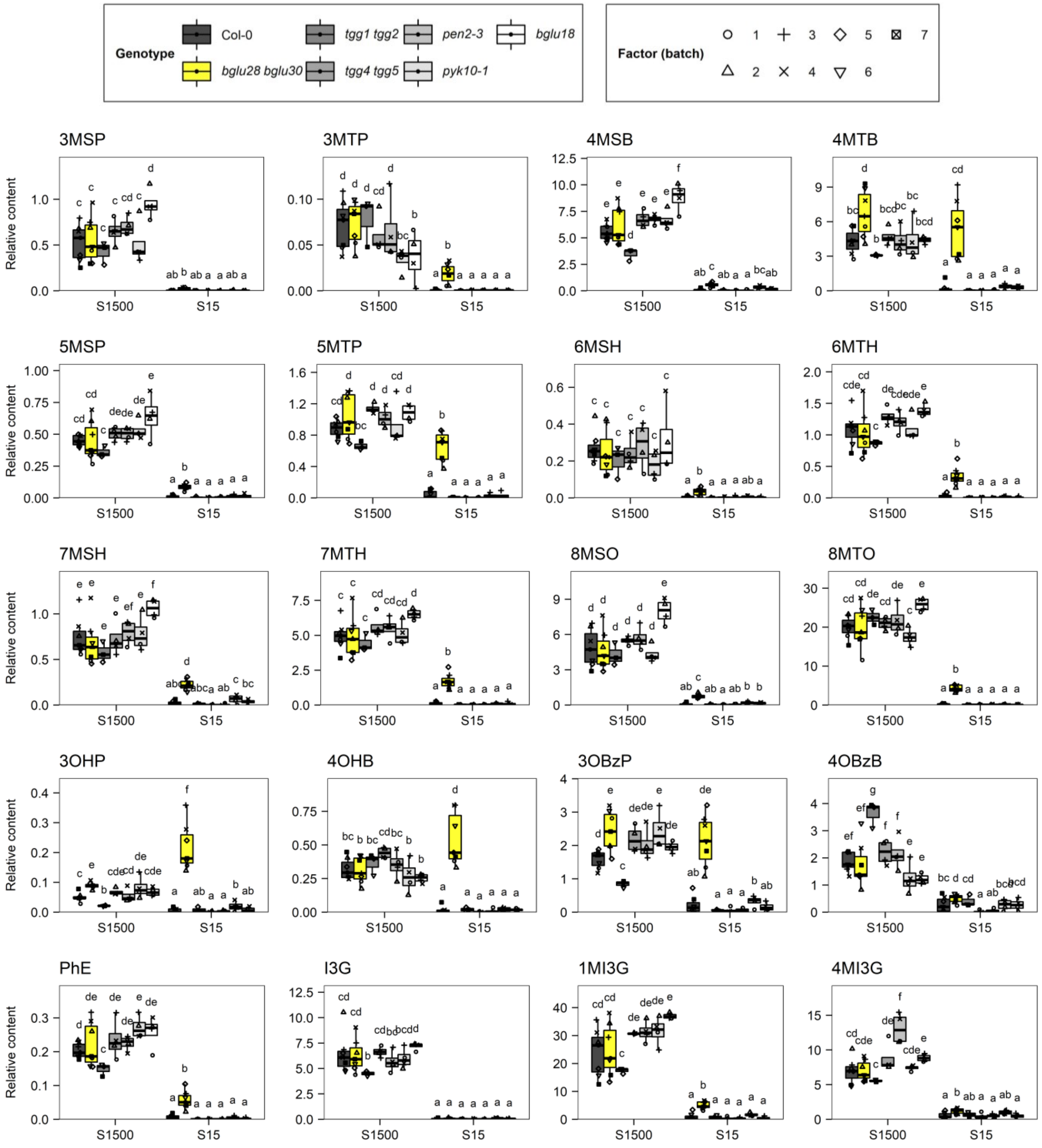

Fig. S12. Relative contents of individual glucosinolate species in the samples shown in Fig. 5*E*. Point shapes indicate individual experimental batches. Letters indicate statistical significance corresponding to two-tailed *t*-tests based on a linear mixed model with batches as a random factor, followed by a correction for multiple comparisons controlling FDR ($P < 0.05$). Abbreviations of GLs correspond to those listed in Table 1.

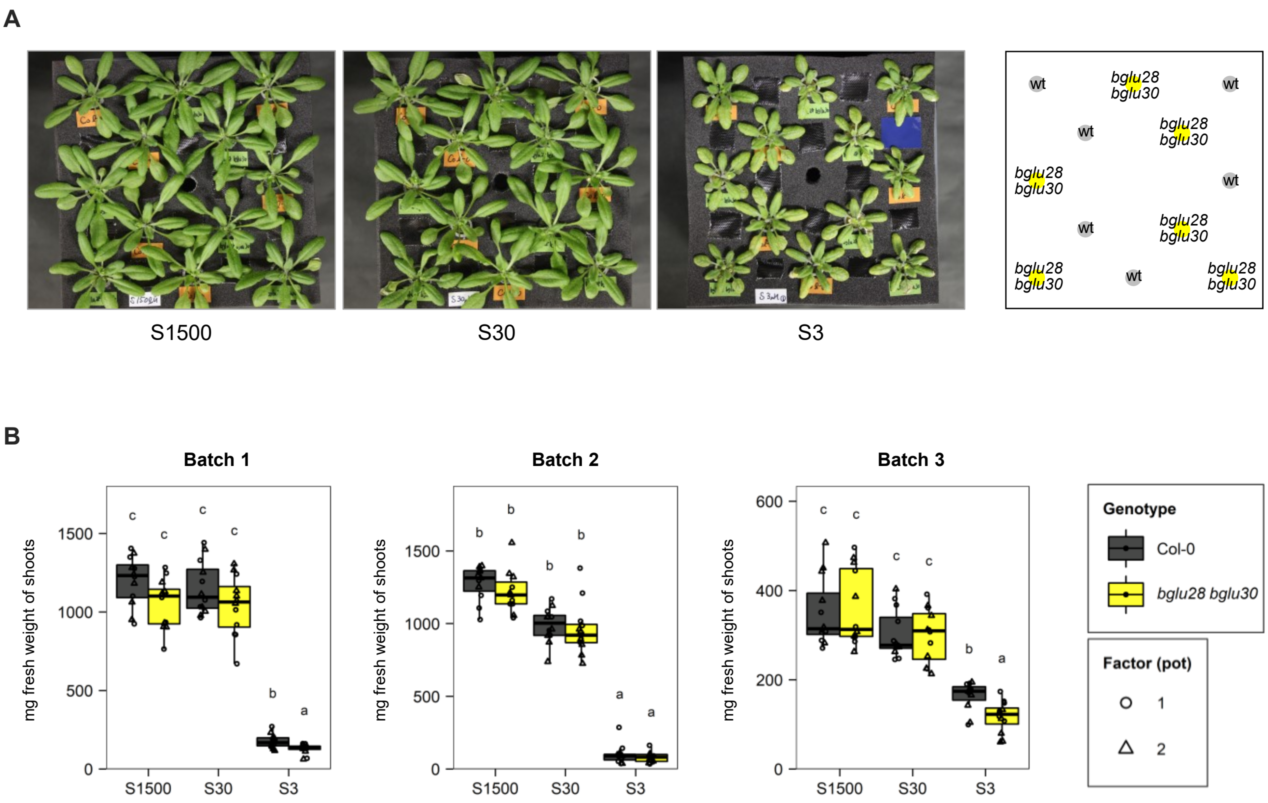

Fig. S13. Growth of the *bglu28 bglu30* mutant in hydroponic culture. (*A*) Respective images of the aerial parts of the four-week-old plants before harvesting (batch 3, pot 1). Col-0 and *bglu28 bglu30* were arranged on each pot as illustrated. (*B*) Fresh weights of the aerial parts in each independent experiment (N=12 individual plants). Letters indicate statistical significance corresponding to two-tailed t-tests based on a linear mixed model with batches as a random factor, followed by a correction for multiple comparisons controlling FDR ($P < 0.05$).

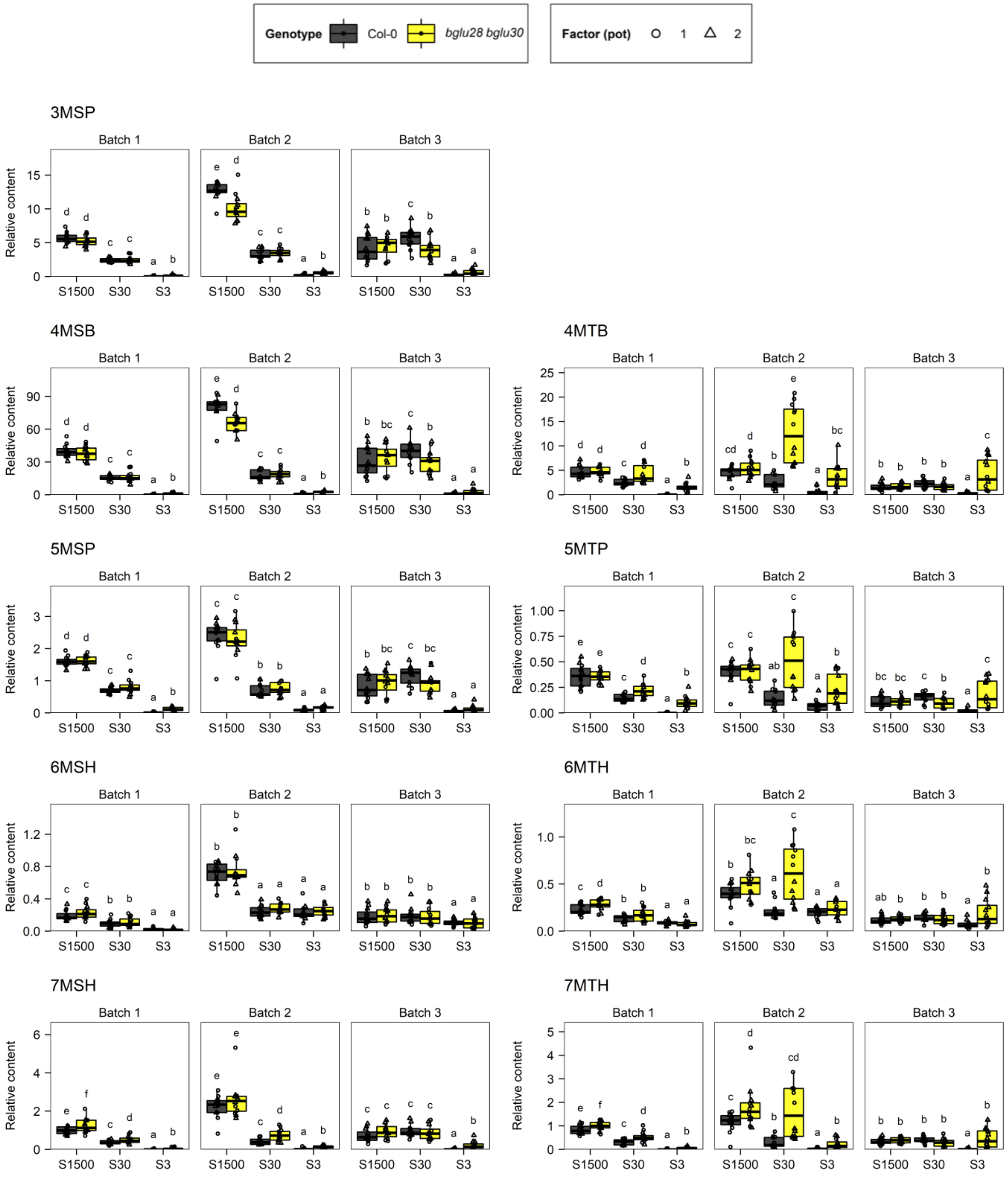

Fig. S14. Glucosinolate contents in the *bglu28 bglu30* mutant grown in hydroponic culture. Relative GL contents in plant tissues of Col-0 and the *bglu28 bglu30* mutant in each independent experiment (N=12 individual plants). Point shapes indicate individual experimental batches. Letters indicate statistical significance corresponding to two-tailed t-tests based on a linear mixed model with batches as a random factor, followed by a correction for multiple comparisons controlling FDR ($P < 0.05$). Abbreviations of GLs correspond to those listed in Table 1.

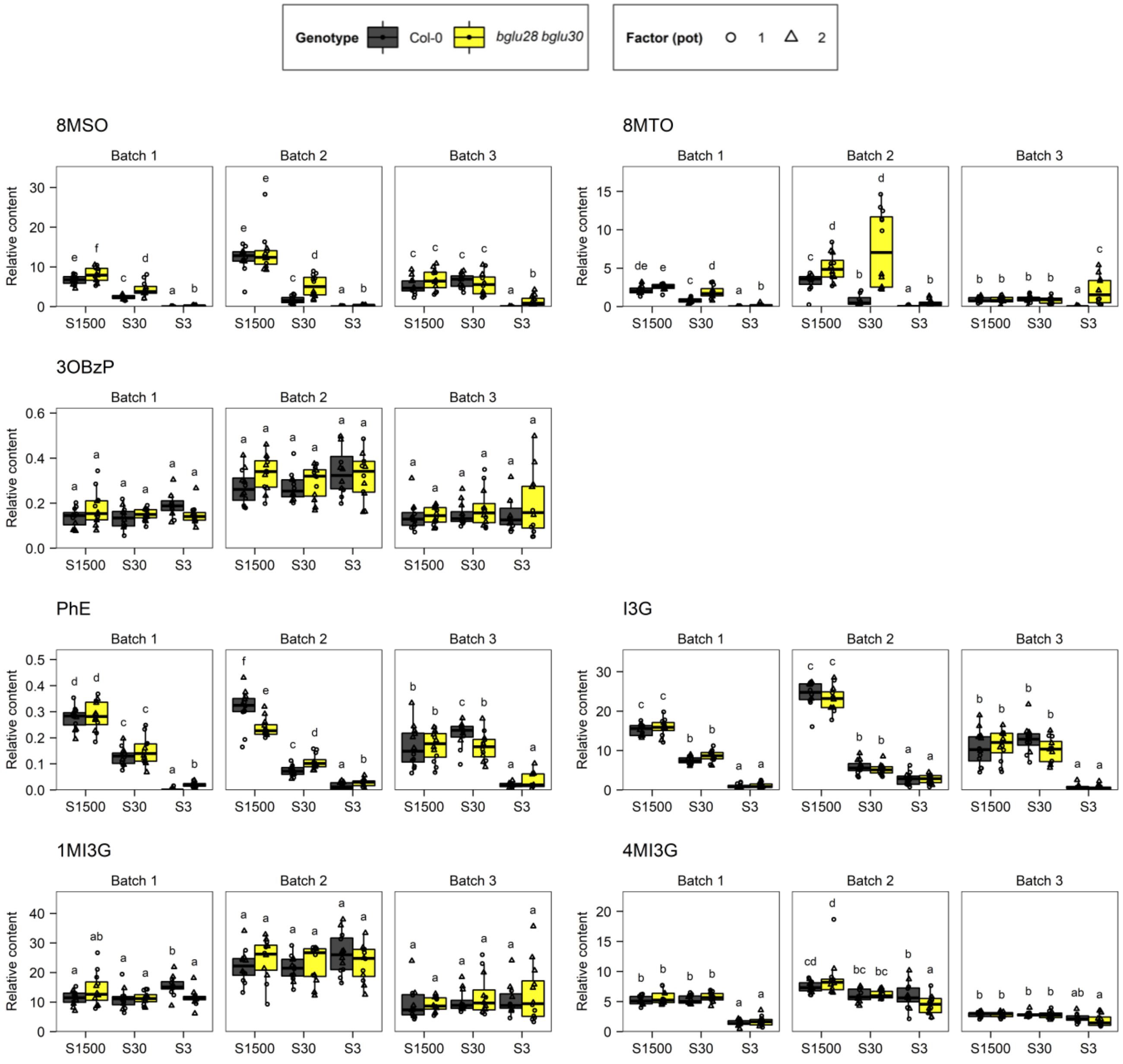

Fig. S14 (continued).

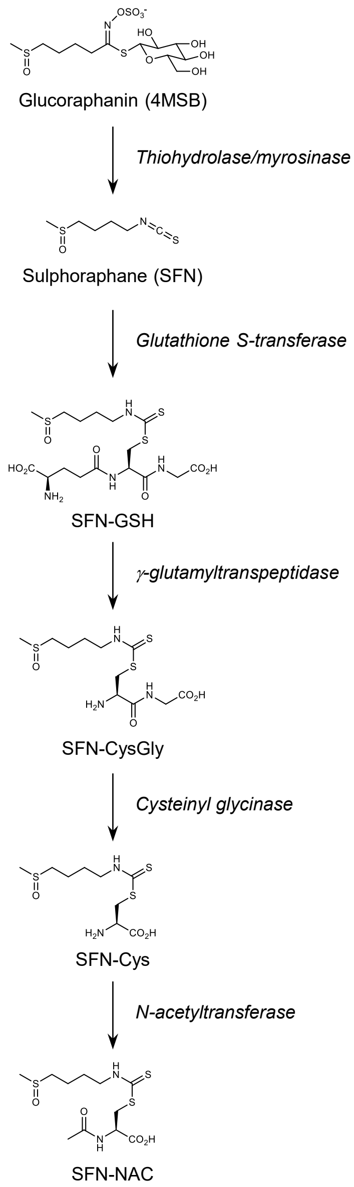

Fig. S15. Glucoraphanin processing in humans. SFN-CysGly and SFN-NAC, conjugates of SFN with cysteinylglycine and with *N*-acetylcysteine, respectively.

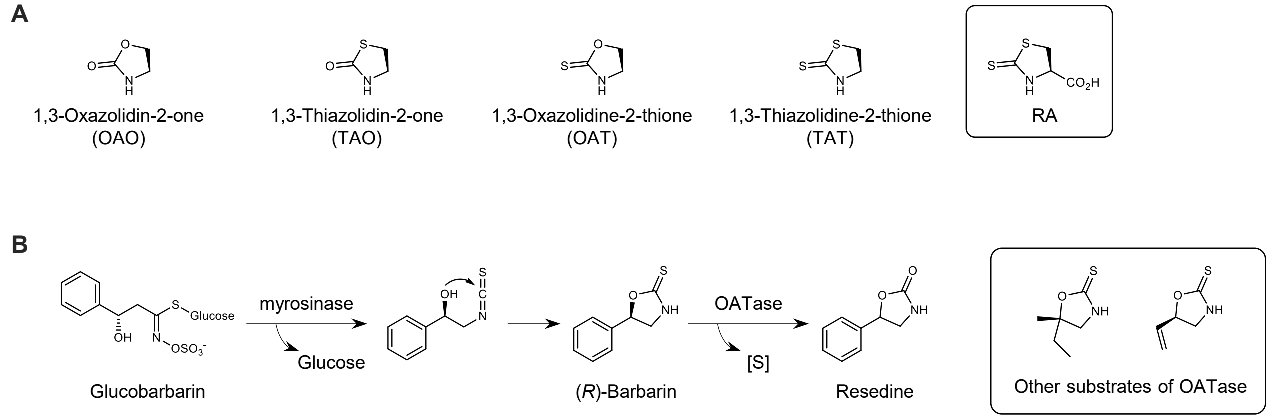

Fig. S16. Sulfur-oxygen exchange reaction potentially involved in the processing of raphanusamic acid (RA). (*A*) Structures of 1,3-oxazolidin-2-one (OAO), 1,3-thiazolidin-2-one (TAO), 1,3-oxazolidine-2-thione (OAT), and 1,3-thiazolidine-2-thione (TAT) rings. RA has a TAT ring. (*B*) Reaction of an enzyme referred to as 'oxazolidinethionase (OATase),' responsible for conversion of OAT to OAO. This reaction was originally found in *Barbarea vulgaris* and *Reseda luteola* and suggested to be involved in GL turnover. In the presence of plant protein extracts, (*R*)-barbarin as well as other OAT substrates can be converted to the corresponding OAOs. Actual enzyme(s) responsible for this reaction and its encoding gene(s) are yet to be identified.

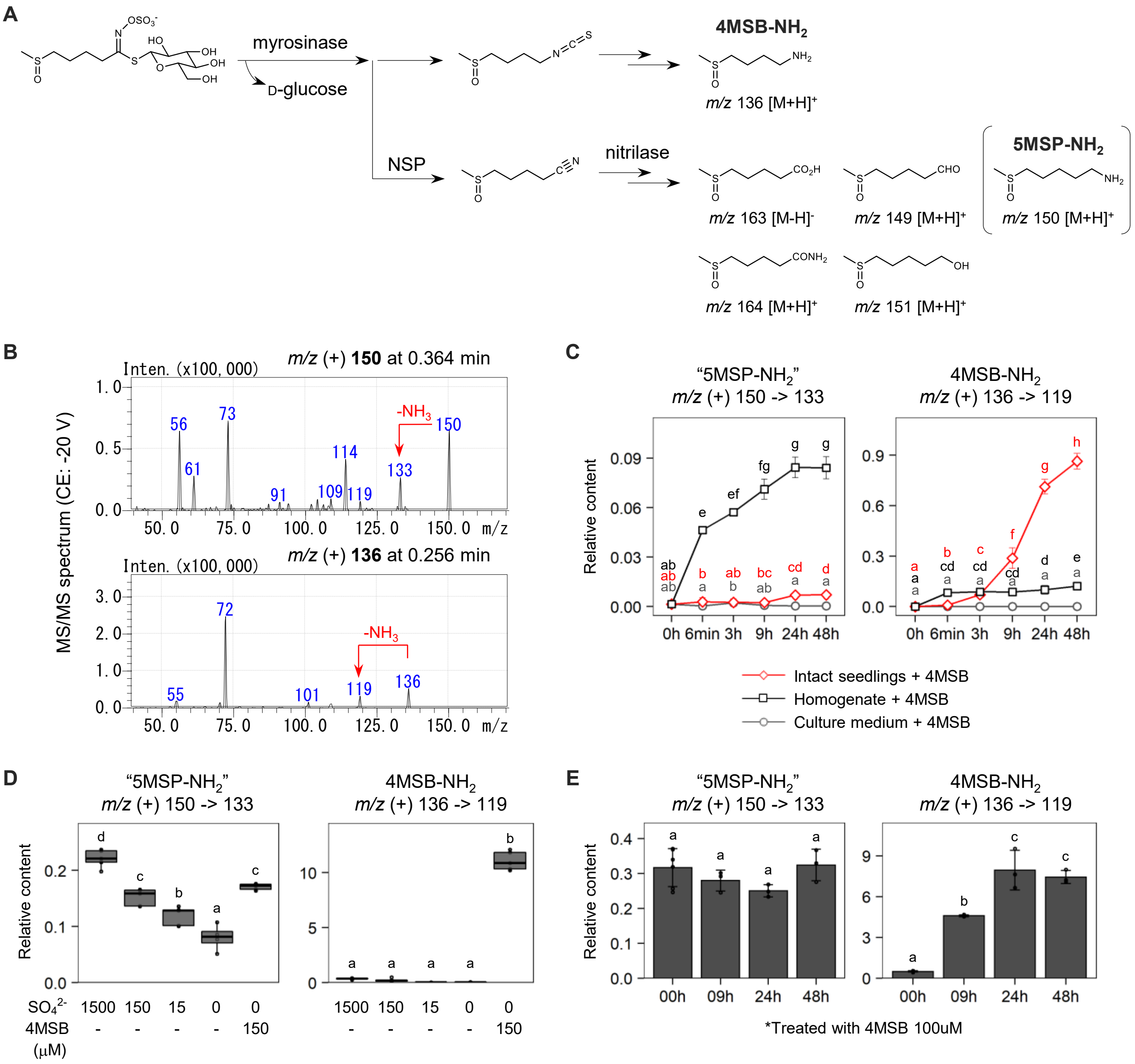

Fig. S17. Detection of glucoraphanin hydrolytic products related to the nitrile pathway. (*A*) Structures and theoretical *m/z* values of five potential nitrilase products. Only one candidate peak, the NH_2 adduct with an extra carbon (5-(methylsulfinyl)pentyl-1-amine, 5MSP-NH_2), was detected in the targeted LCMS system. (B) MS/MS spectrum of 4MSB-NH_2 and potential 5MSP-NH_2 signals. The peak showed a slightly longer retention time than that of 4MSB-NH_2 and the -17 Da fragment ion supported the presence of a primary amine group. (*C*-*E*) Detection of 4MSB-NH_2 and potential 5MSP-NH_2 signals in (*C*) media/intact/homogenate samples treated with 4MSB, in (*D*) tissue extracts from 14-day-old seedlings cultivated at low sulfur conditions, and in (*E*) those from 8- to 10-day-old seedlings after 4MSB treatment. This compound in the media eventually increased in the presence of homogenate (*C*) and its level in tissues had a good correlation with sulfur concentrations (*D*), implying that this is a catabolic product of 4MSB possibly generated via the nitrile pathway. Unlike 4MSB-NH_2, on the other hand, the level in tissue extracts was comparable between S150 and 4MSB150 conditions (*D*) and it did not increase after 4MSB feeding (*E*), suggesting a minor role of the nitrile pathway in the GL breakdown for sulfur reallocation. However, it needs to be noted that these results are solely based on theoretical *m/z* without confirmation using authentic standards. Point shapes indicate individual experimental batches. Letters indicate statistical significance corresponding to two-tailed t-tests based on a linear mixed model with batches as a random factor, followed by a correction for multiple comparisons controlling FDR ($P < 0.05$). Error bars indicate SD.

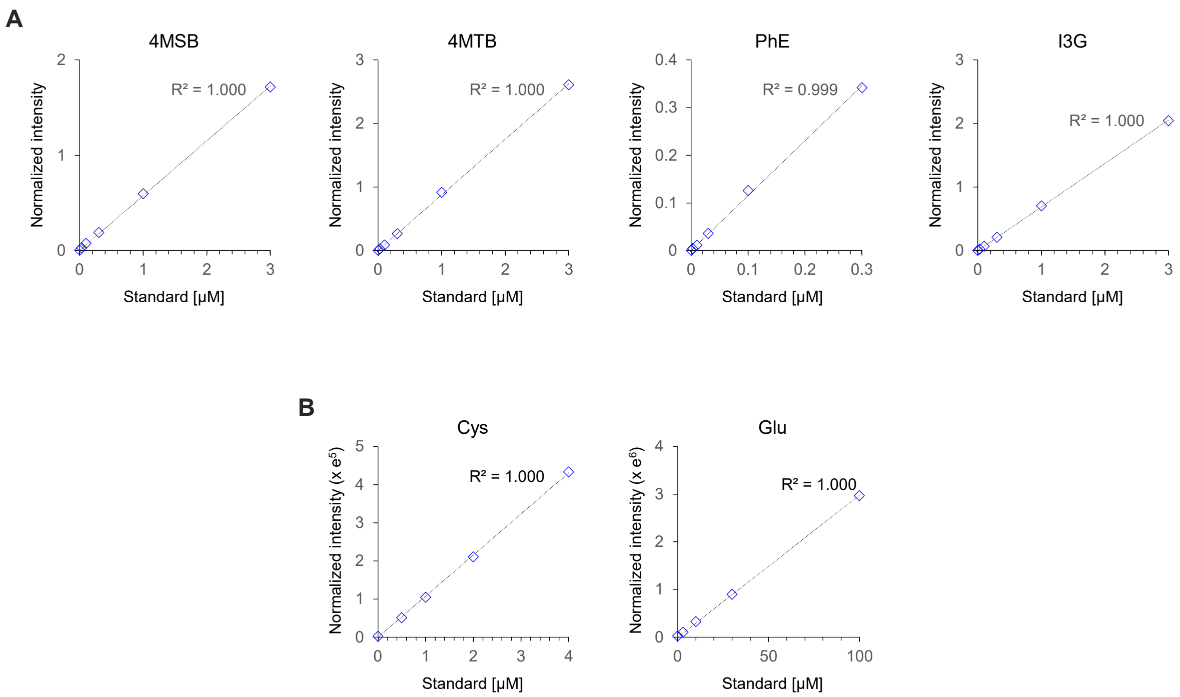

Fig. S18. Calibration curves of standard compounds on triple quadrupole LCMS. (*A*) Standards for glucosinolate quantitation and (*B*) Cys and Glu standards for 5-oxoprolinase assay. Abbreviations of glucosinolate correspond to those listed in Table 1. A series of solutions (2 μL) were injected before and after samples. Signals normalized by an internal standard are shown.

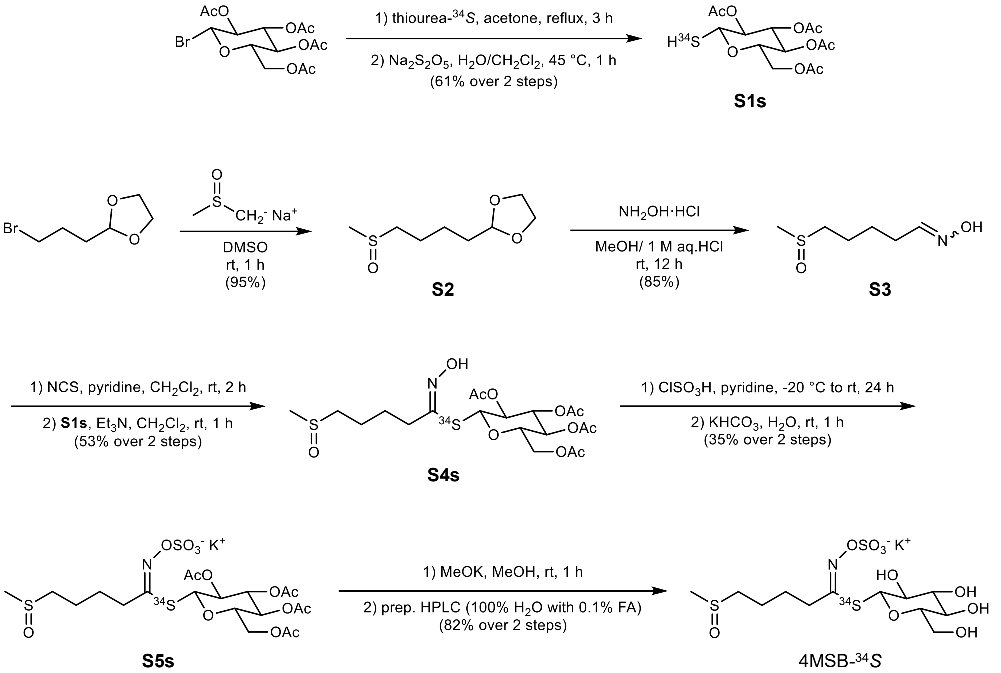

Scheme S1. Synthesis of ^{34}S-labeled glucoraphanin (4MSB-^{34}*S*).

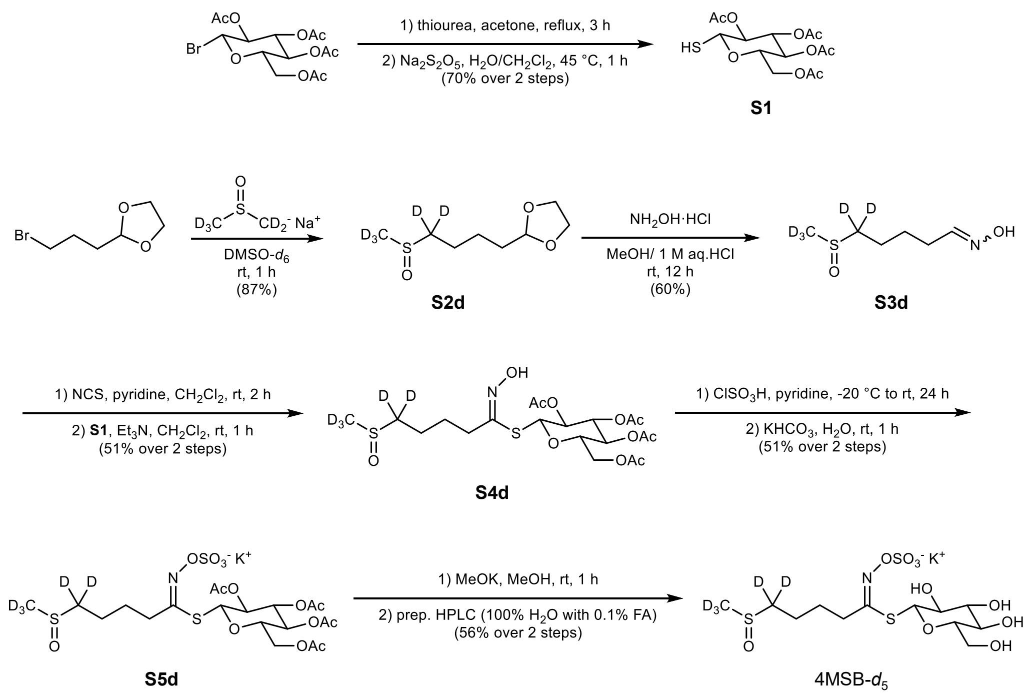

Scheme S2. Synthesis of deuterium-labeled glucoraphanin (4MSB-d_5).

Table S1. Absolute concentrations of Cys, GSH, and glucosinolates in the control groups.

Age	8 days old[a]		14 days old[b]		4 weeks old[c]
Tissue	Whole seedlings		Whole seedlings		Rosette leaves
Condition	S1500		S1500		S1500
	(nmol/mg dry weight)	(nmol/seedling)	(nmol/mg dry weight)	(nmol/seedling)	(nmol/mg dry weight)
Cys	-[d]	-[d]	0.58 ± 0.12	0.31 ± 0.06	-[d]
GSH	-[d]	-[d]	5.87 ± 1.46	3.11 ± 0.73	-[d]
3MSP[e]	0.14 ± 0.04	0.04 ± 0.01	0.19 ± 0.02	0.10 ± 0.01	1.66 ± 0.34
4MSB	2.50 ± 0.31	0.76 ± 0.04	1.29 ± 0.05	0.68 ± 0.03	10.35 ± 2.33
5MSP[e]	0.13 ± 0.01	0.04 ± 0.00	0.11 ± 0.00	0.06 ± 0.00	0.32 ± 0.07
6MSH[e]	0.09 ± 0.01	0.03 ± 0.00	0.09 ± 0.00	0.05 ± 0.00	0.08 ± 0.02
7MSH[e]	0.17 ± 0.03	0.05 ± 0.01	0.17 ± 0.02	0.09 ± 0.01	0.21 ± 0.05
8MSO[e]	0.79 ± 0.14	0.24 ± 0.05	1.03 ± 0.06	0.54 ± 0.06	1.34 ± 0.29
3MTP[f]	0.01 ± 0.01	0.00 ± 0.00	0.01 ± 0.00	0.01 ± 0.00	0.01 ± 0.00
4MTB	2.30 ± 0.48	0.70 ± 0.08	0.42 ± 0.06	0.22 ± 0.04	0.30 ± 0.07
5MTP[f]	0.26 ± 0.03	0.08 ± 0.00	0.08 ± 0.01	0.04 ± 0.01	0.02 ± 0.01
6MTH[f]	0.18 ± 0.02	0.06 ± 0.00	0.12 ± 0.01	0.06 ± 0.01	0.02 ± 0.01
7MTH[f]	1.12 ± 0.17	0.34 ± 0.04	0.58 ± 0.06	0.31 ± 0.05	0.07 ± 0.01
8MTO[f]	3.79 ± 0.50	1.16 ± 0.14	2.22 ± 0.16	1.17 ± 0.14	0.17 ± 0.04
3OHP	-[d]	-[d]	-[d]	-[d]	-[d]
4OHB	-[d]	-[d]	-[d]	-[d]	-[d]
3OBzP	-[d]	-[d]	-[d]	-[d]	-[d]
4OBzB	-[d]	-[d]	-[d]	-[d]	-[d]
PhE	0.02 ± 0.00	0.01 ± 0.00	0.02 ± 0.00	0.01 ± 0.00	0.02 ± 0.00
I3G	0.70 ± 0.06	0.22 ± 0.02	0.84 ± 0.04	0.44 ± 0.04	2.32 ± 0.60
1MI3G[g]	3.84 ± 0.35	1.18 ± 0.06	3.08 ± 0.27	1.62 ± 0.21	1.44 ± 0.97
4MI3G[g]	1.18 ± 0.14	0.36 ± 0.03	0.81 ± 0.04	0.42 ± 0.03	0.54 ± 0.06

[a-c]Mean ± SD are shown. *N* =6 (a,c) and *N* =4 (b).

[d]Not tested.

[e-g]Estimated from the calibration curves of 4MTB (e), 4MSB (f) and I3G (g). Abbreviations correspond to those listed in Table 1.

Table S2. List of possibly deuterated metabolites detected in the untargeted metabolomics.

Pair	ID[a]	t_R (min)[b]	*m/z*	Annotation[c]	Plausible molecular formula (theoretical *m/z*, Δ (ppm))[d]	MS/MS fragments (common, **potentially labelled**, and other ions)[e]
1	P_0035	2.114	103.0581		$C_5H_{11}S^+$ (103.0576, Δ = 4.9)?	**61** **61**
	P_0051	2.100	108.0895		$C_5H_6D_5S^+$ (108.0890, Δ = 4.8)?	**63** **64** 107
2	P_0071	2.351	114.0378		$C_5H_8NS^+$ (114.0372, Δ = 5.3)?	
	P_0094	2.345	116.0504		$C_5H_6D_2NS^+$ (116.0498, Δ = 6.0)?	
3	P_0109	0.956	119.0531		$C_5H_{11}OS^+$ (119.0525, Δ = 4.9)?	
	P_0113	0.948	124.0848		$C_5H_6D_5OS^+$ (124.0839, Δ = 7.3)?	123
4	P_0203	0.960	136.0797	4MSB-NH_2	$C_5H_{14}NOS^+$ (136.0791, Δ = 4.7)	72 73 **72** **119** 73 101 118
	P_0212	0.956	141.1107		$C_5H_9D_5NOS^+$ (141.1104, Δ = 1.8)	72 73 **74** **124** 75 95 99 125 126 140
5	P_0197	1.960	137.0637		$C_5H_{13}O_2S^+$ (137.0631, Δ = 4.5)?	97
	P_0235	1.965	142.0941		$C_5H_8D_5O_2S^+$ (142.0945, Δ = -2.5)?	
6	P_0241	2.252	147.0488		$C_6H_{11}O_2S^+$ (147.0474, Δ = 9.3)?	**119** 55 121 130 146
	P_0272	2.244	152.0794		$C_6H_6D_5O_2S^+$ (152.0788, Δ = 3.9)?	**124** 91 94 101 137
7	P_0322	2.946	162.0414		$C_6H_{12}NS_2^+$ (162.0406, Δ = 5.1)?	**82** 87 131 132
	P_0367	2.919	167.0724		$C_6H_7D_5NS_2^+$ (167.0720, Δ = 2.7)?	**84** 58 116 123
8	P_0391	2.745	178.0360	SFN	$C_6H_{12}NOS_2^+$ (178.0355, Δ = 2.9)	
	P_0423	2.741	183.0677		$C_6H_7D_5NOS_2^+$ (183.0669, Δ = 4.6)	83 116 117
9	P_0404	2.948	180.0518		$C_6H_{14}NOS_2^+$ (180.0511, Δ = 3.7)?	**80** **132** 87 114 124 133 137 141 152
	P_0445	2.916	185.0830		$C_6H_9D_5NOS_2^+$ (185.0825, Δ = 2.6)?	**82** **134** 52 72 92 99 102 108 114 146
10	P_0495	1.786	196.0467		$C_6H_{14}NO_2S_2^+$ (196.0460, Δ = 3.3)?	72 **99** **129** **130** **132** **132** 85 89 102 128 146
	P_0482	1.780	201.0793		$C_6H_9D_5NO_2S_2^+$ (201.0774, Δ = 9.3)?	72 **102** **132** **135** **134** **135** 79 98 114 183 187
11	P_0618	3.010	232.1008		$C_{10}H_{18}NO_3S^+$ (232.1002, Δ = 2.6)?	**61** **103** 135 150 193
	P_0653	2.982	237.1324		$C_{10}H_{13}D_5NO_3S^+$ (237.1316, Δ = 3.5)?	**64** **108** 107
12	P_0663	3.008	249.1276		$C_{10}H_{21}N_2O_3S^+$ (249.1267, Δ = 3.5)?	130 **103** **104** **105** **105** **186** **232** **233** 84 120 188
	P_0675	2.985	254.1590		$C_{10}H_{16}D_5N_2O_3S^+$ (254.1581, Δ = 3.5)?	130 **108** **107** **107** **110** **191** **237** **236** 137 140 253
13	P_0782	2.351	299.0555	SFN-Cys	$C_9H_{19}N_2O_3S_3^+$ (299.0552, Δ = 0.9)	122 **72** **114** **119** **136** **137** **138** **138** **178** 70 180
	P_0796	2.346	304.0873		$C_9H_{14}D_5N_2O_3S_3^+$ (304.0866, Δ = 2.3)	122 **74** **116** **124** **141** **142** **140** **143** **183** 91
14	P_0859	0.969	331.1357		n.d.[f]	136 152 137 314 134 109 150 314
	P_0876	0.968	336.1676		n.d.[f]	
15	P_0905	2.741	356.0769		$C_{11}H_{22}N_3O_4S_3^+$ (356.0767, Δ = 0.6)?	179 **114** **136** **137** 70 150 162 180 221 244
	P_0924	2.720	361.1091		$C_{11}H_{17}D_5N_3O_4S_3^+$ (361.1081, Δ = 2.8)?	179 **116** **141** **140** 124 143 227 236 277 289
16	P_0901	1.783	358.0995		$C_{12}H_{24}NO_7S_2^+$ (358.0989, Δ = 1.8)?	**132** **196** **198** 84 87 109 114 128 240 241
	P_0918	1.779	363.1288		$C_{12}H_{19}D_5NO_7S_2^+$ (363.1303, Δ = -4.0)?	**134** **201** **203** 91 136 166 330
17	P_0990	0.994	396.0544		n.d.[f]	
	P_1001	0.992	401.0867		n.d.[f]	
18	P_1015	2.745	410.0876		$C_{14}H_{24}N_3O_5S_3^+$ (410.0873, Δ = 0.8)?	153 154 199 233 275
	P_1035	2.726	415.1173		$C_{14}H_{19}D_5N_3O_5S_3^+$ (415.1186, Δ = -3.2)?	153 154 199 233 275 99 124

Table S2 (continued).

Pair	ID[a]	t_R (min)[b]	*m/z*	Annotation[c]	Plausible molecular formula (theoretical *m/z*, *Δ* (ppm))[d]	MS/MS fragments (common, **potentially labelled**, and other ions)[e]
19	N_0443	2.946	420.0454	4MTB	$C_{12}H_{22}NO_9S_3^-$ (420.0462, *D* = -1.9)	75 96 97 99 195 259 **178** 80 85 101
	N_0433	2.918	425.0767		$C_{12}H_{17}D_5NO_9S_3^-$ (425.0776, *Δ* = -2.1)	75 96 97 99 195 259 **183** 98 275 424
20	P_1042	2.749	428.0979	SFN-γEC	$C_{14}H_{26}N_3O_6S_3^+$ (428.0978, *Δ* = 0.2)	122 130 217 251 **114** **136** **178** **299** 138 188
	P_1057	2.745	433.1271		$C_{14}H_{21}D_5N_3O_6S_3^+$ (433.1292, *Δ* = -4.9)	122 130 217 251 **116** **141** **183** **304** 305 306
21	N_0477	1.783	436.0401	4MSB	$C_{12}H_{22}NO_{10}S_3^-$ (436.0411, *Δ* = -2.4)	75 96 97 **178** **372** **373** 99 194 275 374
	N_0465	1.780	441.0711		$C_{12}H_{17}D_5NO_{10}S_3^-$ (441.0725, *Δ* = -3.2)	75 96 97 **180** **375** **375** 115 195 259 374 440
22	P_1131	2.736	467.1067		$C_{16}H_{27}N_4O_6S_3^+$ (467.1087, *Δ* = -4.3)?	
	P_1127	2.727	472.1393		$C_{16}H_{27}N_4O_6S_3^+$ (472.1401, *Δ* = -1.7)?	
23	P_1148	2.741	485.1184	SFN-GSH	$C_{16}H_{29}N_4O_7S_3^+$ (485.1193, *Δ* = -1.9)	162 179 308 199 **114** **136** **172** **178** **356** 145
	P_1139	2.725	490.1494		$C_{16}H_{24}D_5N_4O_7S_3^+$ (490.1507, *Δ* = -2.6)	162 179 308 199 **116** **141** **174** **183** **361** 489
24	N_0652	0.995	571.9646		n.d.[f]	436 436 97 436
	N_0646	0.994	576.9961		n.d.[f]	
25	N_0641	2.741	581.0703		$C_{16}H_{29}N_4O_{11}S_4^+$ (581.0721, *Δ* = -3.1)?	97 306 237 288 324
	N_0671	2.722	586.1015		$C_{16}H_{24}D_5N_4O_{11}S_4^+$ (586.1035, *Δ* = -3.4)?	97 306 99 308 488 490
26	N_0757	2.744	910.1930		n.d.[f]	306 483 250 254 249 272 289 251
	N_0755	2.730	920.2535		n.d.[f]	

[a]Corresponds to the Record ID in *SI Appendix*, Dataset S1 (P/N_xxxx).

[b]Retention time.

[c]Abbreviations correspond to those used in the main text.

[d]Molecular formula was predicted using ChemCalc (www.chemcalc.org). *Δ* is the mass difference between experimental and theoretical *m/z* values.

[e]Fragment ions with the top 10 intensities are shown. Ions common in the pair are underlined, and those potentially labelled with D_2, D_3, or D_5 under 4MSB-d_5 treatment are bolded.

[f]Not determined due to mutiple candidates.

Table S3. List of records detected in the untargeted metabolomics, whose levels were influenced by both 4MSB and 4MSB-d_5 treatments.

ID[a]	t_R (min)[b]	m/z	r_S[c]			Annotation (KNApSAcK)[d]	MS/MS fragments[e]
			4MSB vs 4MSB-d_5	DW vs 4MSB	DW vs 4MSB-d_5		
N_0104	2.493	161.9683	0.97	0.06	0.10	C00007581: 2-Thioxo-1,3-thiazolidine-4-carboxylic acid	58, 91, 102, 118, 119, 128
P_0086	2.497	117.9785	0.94	-0.05	0.01		
P_0959	2.497	380.8796	0.90	-0.27	-0.21		172, 174, 175, 204, 206, 222, 226, 250, 252, 330
P_0408	2.244	179.9789	0.90	0.10	0.04		112, 134, 135, 136
P_0656	1.539	251.0704	0.90	0.05	0.04	C00007507: L-gamma-Glutamyl-L-cysteine	84, 122, 130, 188, 201, 236
N_0267	0.980	274.1028	0.90	0.13	0.17		
P_0838	2.495	324.9437	0.90	0.15	0.03		118, 120, 164, 166, 173, 177, 186, 250, 284
P_0849	2.497	335.8820	0.90	-0.14	-0.04		129, 146, 173, 174, 205, 251, 258
N_0262	0.863	280.9776	0.81	0.08	-0.03		97, 109, 127, 145, 146
N_0295	1.544	306.0759	0.80	-0.55	-0.52	C00001518: L-Glutathione	99, 128, 129, 143, 160, 210, 254, 255, 272, 288
P_0554	0.993	212.0600	0.78	-0.17	-0.14		
N_0303	0.846	302.8385	0.78	-0.14	0.13		131, 132, 156
N_0165	3.400	205.0501	0.76	-0.27	0.02	C00007397: 3-Indolylmethylthiohydroximate	119, 147, 151, 175, 190
P_0377	0.960	175.1187	0.73	0.15	0.10	C00001340: L-Arginine	70, 112, 115, 116, 128, 133, 157
P_0145	1.642	130.0505	0.73	-0.16	-0.24	C00007403: Pyroglutamic acid	84, 85, 102
P_0803	2.514	310.1646	0.71	0.17	0.09		144, 147, 149, 175, 251, 252
P_0862	0.904	331.1002	0.71	0.17	0.10		
N_0304	2.736	322.0927	0.70	-0.11	-0.33	C00007576: Indole-3-carboxylic acid beta-D-glucopyranosyl ester	131, 132, 159
P_0286	2.351	149.0603	0.69	0.17	-0.02	C00000170: trans-Cinnamic acid	98, 103, 131, 134
N_0348	1.545	338.0656	0.68	-0.39	-0.42		58, 67, 88, 143, 145, 181, 256, 263, 272, 274
N_0237	3.400	247.0605	0.67	-0.13	0.13	C00007449: 4-Thiazolidinecarboxylic acid	145, 189, 217, 219, 232, 233
N_0548	5.217	492.1028	0.67	-0.05	-0.25	C00007355: 8-Methylsulphinyloctyl glucosinolate	96, 97, 98, 219, 250, 275
N_0772	5.461	998.2786	0.64	-0.39	-0.52		476, 477, 478, 556, 558, 561, 955, 956, 971
P_0820	2.714	319.1367	0.64	-0.61	-0.40		229, 286
P_0619	0.843	231.9890	0.63	0.10	0.08		
N_0430	1.543	404.0423	0.63	-0.37	-0.02		97, 129, 271, 323
N_0243	1.546	272.0881	0.62	-0.15	-0.32		87, 143, 145, 154, 179, 254
P_0327	1.542	162.0224	0.60	0.13	-0.26		116, 144
P_0830	1.775	313.0130	0.59	-0.62	-0.73		
P_1029	3.906	418.1356	0.59	-0.04	0.04		135, 152
N_0206	3.400	223.0607	0.59	0.13	0.10	C00034327: (E)-Sinapic acid; C00002776: Sinapic acid	96, 147, 149, 150, 164
N_0389	3.503	385.1131	0.57	-0.22	-0.15	C00013592: 1-O-Sinapoyl-beta-D-glucose	101, 119, 164, 179, 190, 195, 205, 206, 223, 224
P_0736	1.539	291.0651	0.56	0.15	0.00		
P_0615	1.545	233.0596	0.55	0.01	-0.34		76, 86, 112, 117, 146, 170, 196
N_0180	2.498	218.1023	0.53	-0.06	-0.58	C00001550: Pantothenic acid	88, 131, 167
P_1178	0.853	519.0152	0.52	-0.15	0.09		

Table S3 (continued).

ID[a]	t_R (min)[b]	*m/z*	r_S[c]			Annotation (KNApSAcK)[d]	MS/MS fragments[e]
			4MSB vs 4MSB-d_5	DW vs 4MSB	DW vs 4MSB-d_5		
P_1073	4.294	441.1371	0.52	-0.08	-0.08		270, 279, 304, 403
N_0703	0.827	632.7256	0.52	0.10	-0.16		129, 131, 213, 297, 298, 381, 382, 465, 467, 549
P_0384	1.544	179.0490	0.51	0.15	-0.41	C00007416: L-Gluconolactone; C00007388: L-Galactono-1,4-lactone	75, 76, 116, 119, 144, 146, 162, 163
N_0228	1.544	254.0778	0.51	-0.28	-0.24		100, 167, 179
P_0058	0.845	104.1076	0.50	0.14	0.09		60
N_0148	1.063	194.0469	0.50	-0.27	0.06		80, 97, 99
P_0551	0.863	212.8456	0.50	0.13	-0.02		104, 123, 196
N_0634	4.123	557.0193	0.48	0.13	0.02		97, 169, 235, 241, 242, 259, 273, 477, 478, 480
N_0426	1.045	404.0553	0.48	-0.16	-0.13		62, 97, 153, 308
N_0537	4.822	478.0865	0.48	-0.01	-0.34	C00007352: 7-(Methylsulphinyl)heptyl glucosinolate	85, 97, 414, 415
P_0485	0.746	200.9735	0.47	-0.03	-0.06		85, 111, 139, 140, 144, 183, 185
P_0889	1.002	346.0479	0.47	0.18	-0.20		136, 217
P_1271	0.859	619.0812	0.47	-0.06	0.17		
P_0244	0.752	146.1658	0.46	-0.17	0.17	C00001431: Spermidine	68, 69, 71, 72, 87, 102, 110, 111, 112, 128
P_0136	0.746	128.0197	0.45	-0.29	0.02		60, 66, 67, 68, 69, 72, 84, 110, 112, 113
P_0188	0.746	132.9987	0.45	-0.34	-0.34		69, 115
P_0426	0.747	182.9626	0.45	-0.03	0.10		85, 150
P_0632	3.763	235.1824	0.45	-0.18	-0.10		84, 86
P_0800	0.969	311.1276	0.45	-0.39	0.19		
P_0731	7.016	274.2758	0.45	-0.41	-0.08		72, 90, 106, 216, 256
N_0086	0.926	146.0457	0.43	0.07	0.18	C00001358: L-Glutamic acid; C00007459: O-Acetyl-L-serine	127, 128, 129, 145
P_0248	2.432	146.0618	0.43	-0.47	-0.41	C00007667: 5-Methylsufinylpentyl nitrile; C00000112: Indole-3-carboxaldehyde	70, 103, 130
N_0519	0.844	470.7606	0.43	-0.12	-0.05		
P_0419	3.154	184.1156	0.43	-0.13	0.15		127, 134, 136, 138, 146
P_0511	2.498	202.1077	0.43	-0.29	0.13		
N_0700	0.831	634.7248	0.43	-0.11	-0.01		
P_0064	0.747	114.9880	0.42	-0.28	-0.33		70, 114
P_0388	2.110	178.1260	0.42	0.15	-0.34		134, 159
P_0402	2.053	180.0884	0.42	0.02	-0.14		81, 122
N_0589	0.748	502.9175	0.42	0.20	-0.06		115, 159, 160, 161, 273, 274, 275, 276, 277, 388
P_0572	0.749	216.9518	0.41	-0.19	0.17		109, 110, 111, 139, 156, 183, 184, 199, 201, 202

[a]Corresponds to the Record ID in *SI Appendix*, Dataset S1 (P/N_xxxx).

[b]Retention time.

[c]Spearman's rank correlation coefficient among samples treated with DW (deionised water, solvent control), with 4MSB, or with 4MSB-d_5.

[d]www.knapsackfamily.com/KNApSAcK_Family (keyword: Arabidopsis).

[e]Fragment ions with the top 10 intensities are shown.

Table S4. Sequence of primers used in the present study.

Genotyping	
SALK_067086-LP	CTGCATTCTGGTTCTTTCTGC
SALK_067086-RP	AAGATACGGTCTCATTTGGGG
SALK_029737-LP	AACACCTAAGTGAGAACCCGC
SALK_029737-RP	ACACATTTGAAATCGTAGCCG
SALK_070446-LP	ATCTTGGCTTCTGGGAATTGA
SALK_070446-RP	TCCTCCCTATTCACGTATCGC
SALK_114084-LP	CTCTCATAACTTCTTGGTGCGTC
SALK_114084-RP	CTGCTACGTTGCATCCATCC
CS69080-LP	AGGAAATATTCTTGGCCATG
CS69080-RP	TTGATTAATATGGCCTGGCTG
SALK_075731-LP	TTTGGCTCCAACAACTTATGG
SALK_075731-RP	ACCGGTAGATGAAAACGATCC
LBb1.3	ATTTTGCCGATTTCGGAAC
o8409	ATATTGACCATCATACTCATTGC

Transient expression in *Nicotiana benthamiana*	
GFP-Nb-F	CATCACCATCATCCCATGGTGAGCAAGGGCGAG
GFP-Nb-R	AGTTAAAGGCCTCGATTACTTGTACAGCTCGTCC
BGLU28-Nb-F	TTCTGCCCAAATTCGATGAAGATGCATTTTTTCATCC
BGLU28-Nb-R	GTGATGGTGATGCCCCCAAACGTTTTCTAAAAATCC
BGLU30-Nb-F	TTCTGCCCAAATTCGATGGCTAAGGGATCGTGG
BGLU30-Nb-R	GTGATGGTGATGCCCATAAAATGAAGATGGGTTCTC
TGG1-Nb-F	TTCTGCCCAAATTCGATGAAGCTTCTTATGCTCGC
TGG1-Nb-R	GTGATGGTGATGCCCTGCATCTGCAAGACTCTTC

Table S5. Retention time and *m/z* values of the precursor and product ions for each metabolite analyzed on a liquid chromatography–tandem mass spectrometry (LC–MS/MS).

Positive ion mode				Negative ion mode			
Metabolite name[a]	t_R (min)[b]	Precursor *m/z*	Product *m/z*	Metabolite name[a]	t_R (min)[b]	Precursor *m/z*	Product *m/z*
Cys	0.26	122	59	3MSP	0.54	422	97
Glu	0.27	148	84	4MSB	1.10	436	97
Met	0.38	150	104	5MSP	2.06	450	97
GSH	0.43	308	162	6MSH	2.22	464	97
RA	1.38	164	59	7MSH	2.38	478	97
SFN	3.19	178	114	8MSO	2.72	492	97
SFN-Cys	2.14	299	178	3MTP	2.24	406	97
SFN-γEC	2.30	429	251	4MTB	2.51	420	97
SFN-GSH	2.31	485	179	5MTP	2.95	434	97
4MSB-NH_2	0.27	136	119	6MTH	3.34	448	97
Cys-^{34}S	0.26	124	61	7MTH	3.59	462	97
Met-^{34}S	0.38	152	106	8MTO	3.79	476	97
GSH-^{34}S	0.43	310	164	3OHP	0.40	376	97
RA-^{34}S	1.38	166	59 / 61[c]	4OHB	0.63	390	97
RA-$^{34}S_2$	1.38	168	61	3OBzP	3.35	480	97
SFN-^{34}S	3.19	180	116	4OBzB	3.51	494	97
SFN-Cys-^{34}S	2.14	301	178 / 180[c]	PhE	2.97	422	97
SFN-Cys-$^{34}S_2$	2.14	303	180	I3G	2.73	447	97
SFN-γEC-^{34}S	2.30	431	251 / 253[c]	1MI3G	3.36	477	97
SFN-γEC-$^{34}S_2$	2.30	433	253	4MI3G	3.10	477	97
SFN-GSH-^{34}S	2.31	487	179 / 181[c]	Procysteine	0.84	146	103
SFN-GSH-$^{34}S_2$	2.31	489	181	5-Oxoproline	0.55	128	82
Lidocaine	2.55	235	86	4MSB-^{34}S	1.10	438	97
				4MTB-^{34}S	2.51	422	97
				4OHB-^{34}S	0.63	392	97
				4OBzB-^{34}S	3.51	496	97
				10-CA	3.06	231	80

[a]Abbreviations correspond to those used in the main text. 10-CA, 10-comphorsulfonic acid.

[b]Retention time. Several authentic glucosinolates (3MSP, 4MSB, 4MTB, PhE, I3G, and 1MI3G) were used to determine retention times. Retention times of the other glucosinolates were estimated from their exact masses, side-chain structures, and generation of a typical fragment ion, HSO_4^- (*m/z* 97).

[c]Independently recorded to distinguish the position of ^{34}S incorporation.

NMR spectroscopic data

^{1}H and ^{13}C NMR spectra of 4-methylsulfinyl-*n*-butyl glucosinolate potassium salt (4MSB) in D_2O.

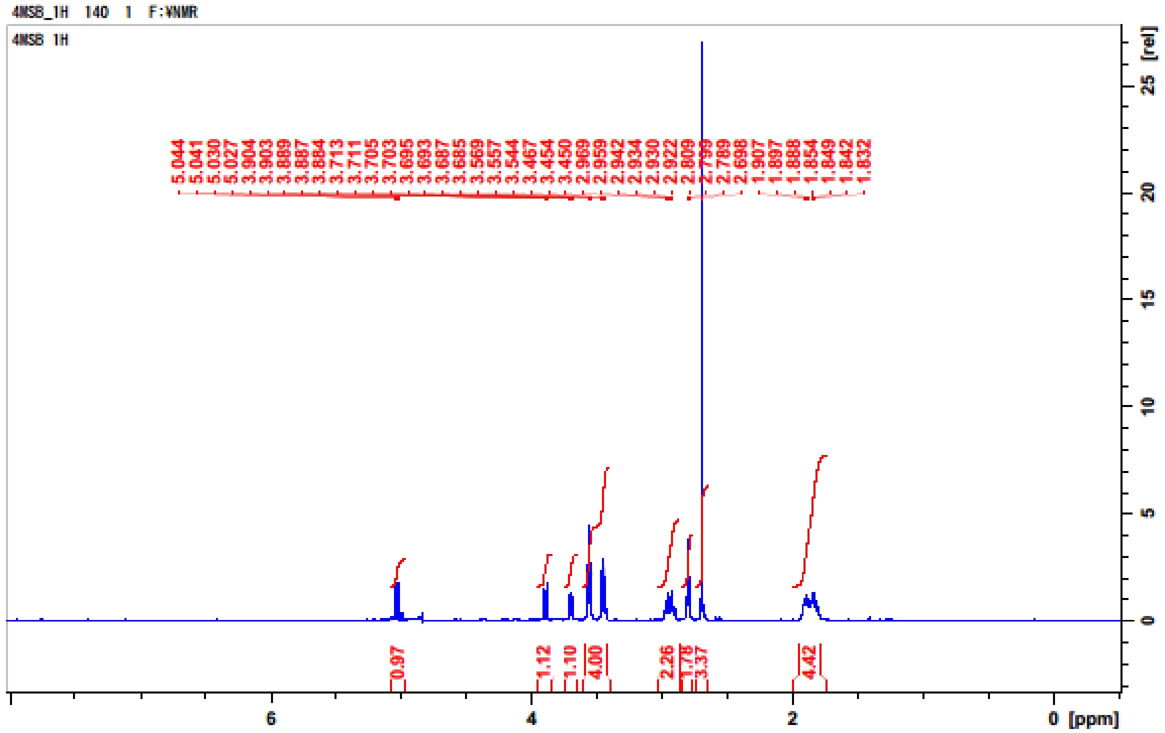

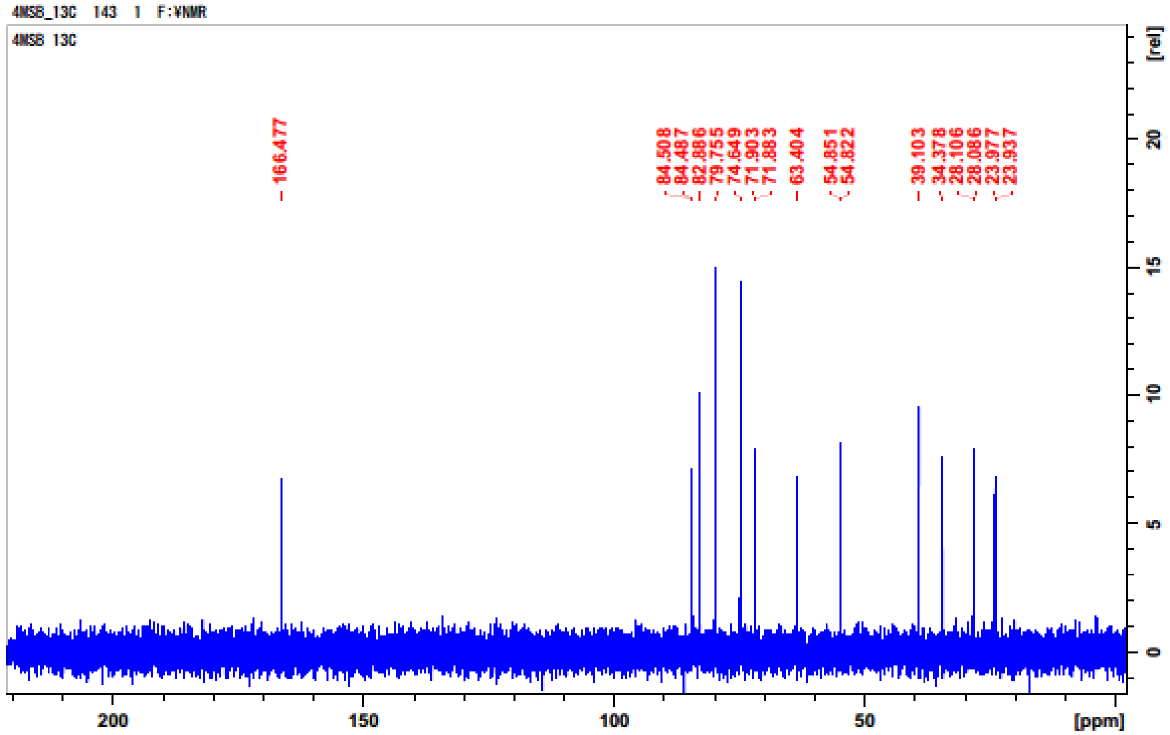

^{1}H and ^{13}C NMR spectra of 4-methylsulfinyl-*n*-butyl glucosinolate-*thiohydroximate*-^{34}S potassium salt (4MSB-^{34}S) in D_2O.

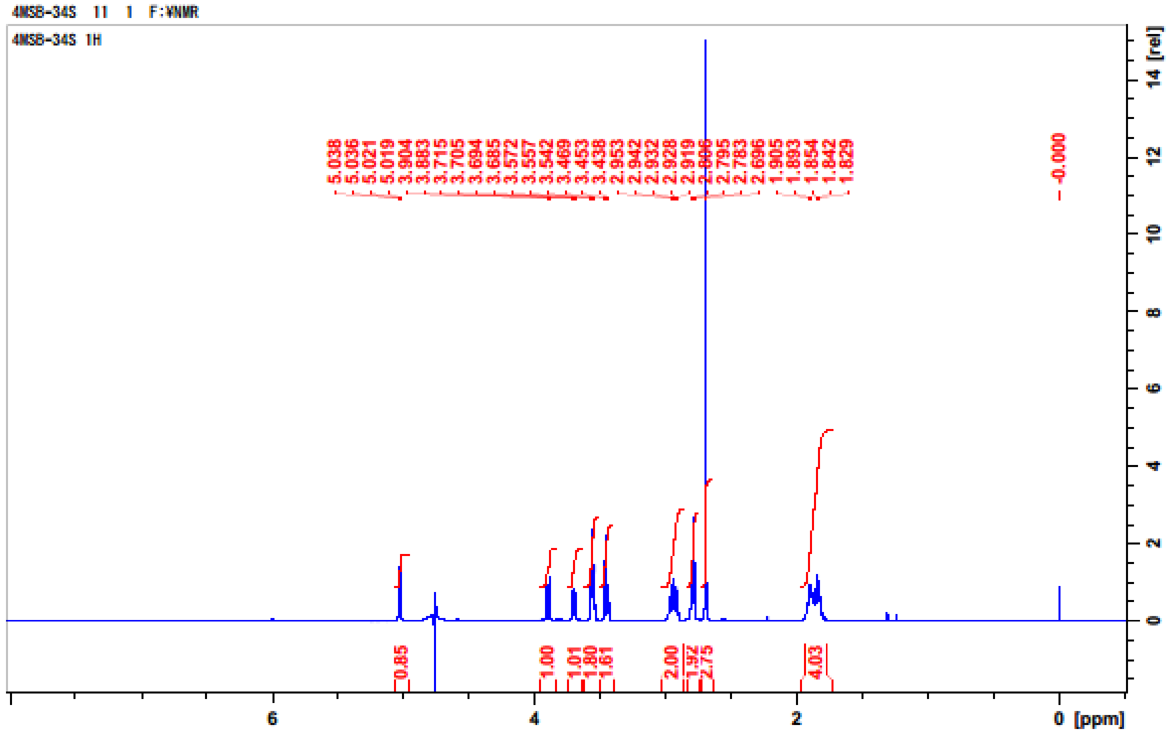

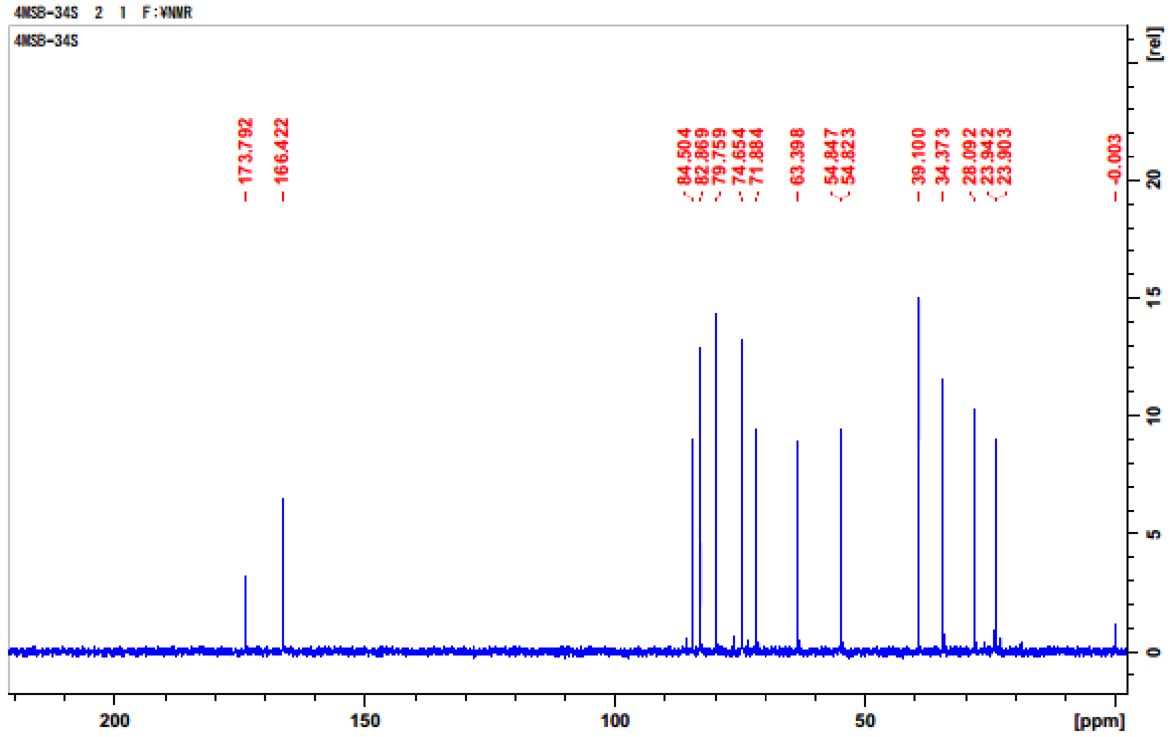

^1H and ^{13}C NMR spectra of 4-(methyl-d_3)sulfinyl-n-butyl-4,4-d_2 glucosinolate potassium salt (4MSB-d_5) in D_2O.

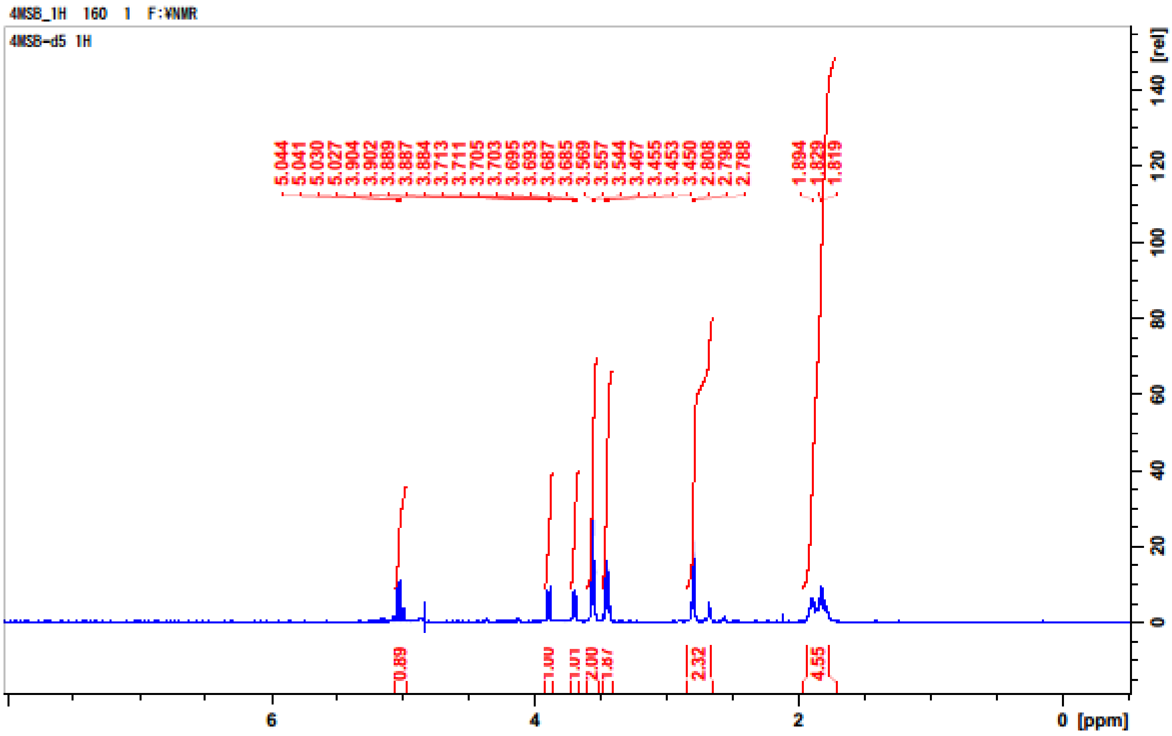

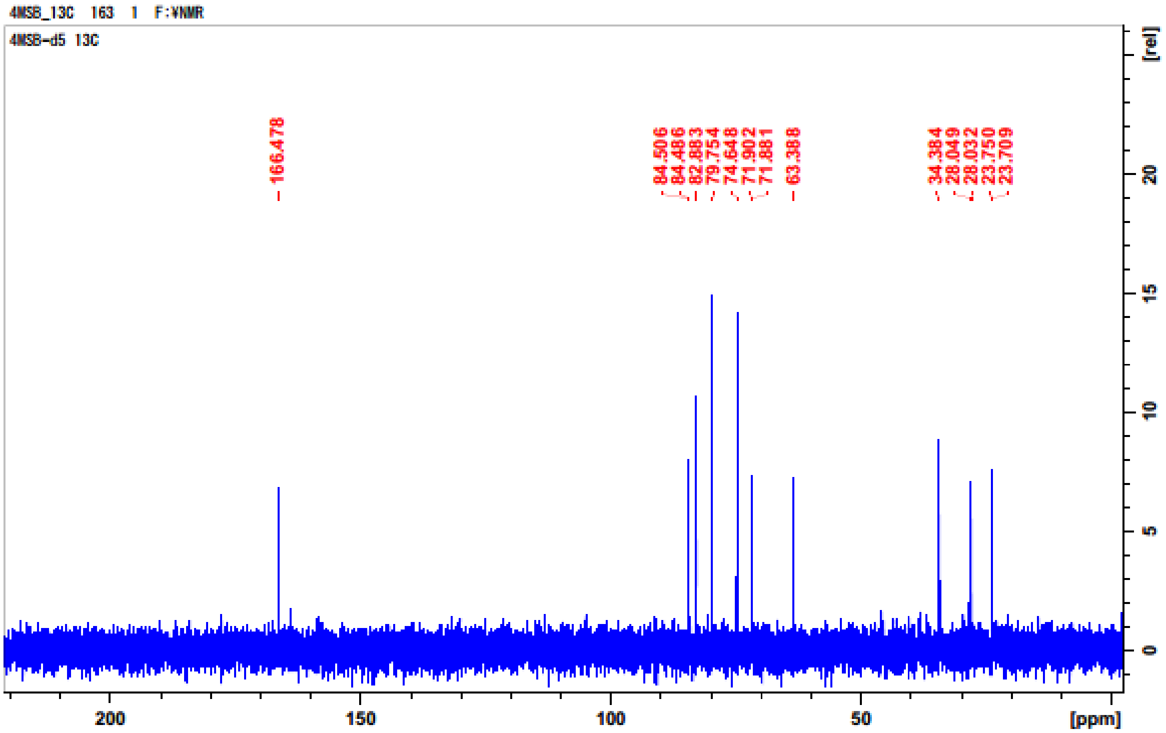

Dataset S1 (separate file). Entire dataset of the untargeted metabolomics.

SI References

1. Q. V. Vo, C. Trenerry, S. Rochfort, A. B. Hughes, A total synthesis of (*R*,*S*)$_S$-glucoraphanin. *Tetrahedron* **69**, 8731-8737 (2013).
2. N. P. Botting, A. A. B. Robertson, J. J. Morrison, The synthesis of isotopically labelled glucosinolates for analysis and metabolic studies. *J. Label Compd. Radiopharm.* **50**, 260-263 (2007).
3. L. Patiny, A. Borel, ChemCalc: A building block for tomorrow's chemical infrastructure. *J. Chem. Inf. Model.* **53**, 1223-1228 (2013).
4. F. Sainsbury, E. C. Thuenemann, G. P. Lomonossoff, pEAQ: versatile expression vectors for easy and quick transient expression of heterologous proteins in plants. *Plant Biotechnol. J.* **7,** 682–693 (2009).
5. S. Asai, K. Ohta, H. Yoshioka, MAPK signaling regulates nitric oxide and NADPH oxidase-dependent oxidative bursts in *Nicotiana benthamiana*. *Plant Cell* **20,** 1390–1406 (2008).
6. J. Li, B. G. Hansen, J. A. Ober, D. J. Kliebenstein, B. A. Halkier, Subclade of flavin-monooxygenases involved in aliphatic glucosinolate biosynthesis. *Plant Physiol.* **148**, 1721-1733 (2008).
7. B. G. Hansen, D. J. Kliebenstein, B. A. Halkier, Identification of a flavin-monooxygenase as the *S*-oxygenating enzyme in aliphatic glucosinolate biosynthesis in Arabidopsis. *Plant J.* **50**, 902-910 (2007).
8. E. K. Chan, H. C. Rowe, D. J. Kliebenstein, Understanding the evolution of defense metabolites in Arabidopsis thaliana using genome-wide association mapping. *Genetics* **185**, 991-1007 (2010).
9. D. J. Kliebenstein *et al.*, Genetic control of natural variation in Arabidopsis glucosinolate accumulation. *Plant Physiol.* **126**, 811-825 (2001).
10. S. Lee *et al.*, Benzoylation and sinapoylation of glucosinolate R-groups in Arabidopsis. *Plant J.* **72**, 411-422 (2012).
11. U. Wittstock, E. Kurzbach, A.-M. Herfurth, E. J. Stauber, "Glucosinolate breakdown" in *Advances in Botanical Research,* S. Kopriva, Ed. (Academic Press, Cambridge, MA, 2016), vol. 80, pp. 125–169.
12. L. Zhang *et al.*, Sulfur deficiency-induced glucosinolate catabolism attributed to two beta-glucosidases, BGLU28 and BGLU30, is required for plant growth maintenance under sulfur deficiency. *Plant Cell Physiol.* **61**, 803–813 (2020).
